# ﻿Systematic revision of the snorkel snail genus *Rhiostoma* Benson, 1860 (Gastropoda, Caenogastropoda, Cyclophoridae) with descriptions of new species

**DOI:** 10.3897/zookeys.1142.90097

**Published:** 2023-01-24

**Authors:** Piyoros Tongkerd, Sakboworn Tumpeesuwan, Khamla Inkhavilay, Pongpun Prasankok, Ekgachai Jeratthitikul, Somsak Panha, Chirasak Sutcharit

**Affiliations:** 1 Animal Systematics Research Unit, Department of Biology, Faculty of Science, Chulalongkorn University, Bangkok 10330, Thailand Chulalongkorn University Bangkok Thailand; 2 Present address: Department of Biology, Faculty of Science, Mahasarakham University, Kantharawichai, Maha Sarakham 44150 Thailand Mahasarakham University Maha Sarakham Thailand; 3 Department of Biology, Faculty of Natural Science, National University of Laos, P.O. Box 7322, Dongdok, Vientiane, Laos National University of Laos Vientiane Laos; 4 School of Biology, Institute of Science, Suranaree University of Technology, Nakhon Ratchasima 30000, Thailand Suranaree University of Technology Nakhon Ratchasima Thailand; 5 Animal Systematics and Molecular Ecology Laboratory, Department of Biology, Faculty of Science, Mahidol University, Bangkok 10400, Thailand Mahidol University Bangkok Thailand

**Keywords:** DNA barcoding, endemic, Indochina, limestones, operculum, Prosobranchia, taxonomy

## Abstract

The snorkel snail genus *Rhiostoma* Benson, 1860 is comprised of terrestrial cyclophorid snails with wide-ranging species diversity and radiation in Southeast Asia. The typical characters of the genus are a depressed shell, a detached and descending portion of the last whorl with a distinctive peristomal breathing device attached, and a calcareous cup-shaped operculum. Herein, we have revised the systematics of extant species based on shell morphology combined with COI barcoding. From these thirty recognised species, twelve are described as new to science: R. ? amarapuraense**sp. nov.**, *R.anceyi***sp. nov.**, *R.breviocollar***sp. nov.**, *R.ebenozostera***sp. nov.**, *R.cheliopegma***sp. nov.**, *R.furfurosum***sp. nov.**, *R.gnomus*, **sp. nov.**, *R.lannaense***sp. nov.**, *R.laoense***sp. nov.**, *R.platymorpha***sp. nov.**, *R.rhothonotaphrosa***sp. nov.**, and *R.tigrina***sp. nov.** All conchological characters are provided via illustrations of type specimens and living snails, and descriptions of the shells and radulae. Phylogenetic analysis based on partial COI gene sequences strongly supports the designated morphospecies and a monophyletic *Rhiostoma*, confirming that all pterocyclinid snails with a calcareous, cup-shaped operculum belong to the same clade. A high intra-specific divergence was observed in *R.jalorensis* and *R.housei* populations from locations in close proximity, suggesting a lower dispersal and higher level of isolation. The low inter-specific divergence found in *R.hainesi*, *R.samuiense*, *R.asiphon*, and *R.rhothonotaphrosa***sp. nov.** supports their recent diversification and local adaptation, and is congruent with their marked morphological differences. Finally, nine formerly *Rhiostoma*-placed species were reclassified into either the genus *Cyclotus* or the genus *Opisthoporus*.

## ﻿Introduction

The Cyclophoridae Gray, 1847 constitutes a prominent group of land snail distributed throughout tropical regions of Africa and Madagascar to South and Southeast Asia, southern China, Japan, New Guinea, and northern Australia (Kobelt 1902; Solem 1959; Azuma 1982; Fretter et al. 1998; Stanisic 1998). They are thought to have been consumed by prehistoric cave-dwelling humans in Vietnam (Rabett et al. 2011; Bellwood and Dizon 2013; O’Donnella et al. 2020), and today are utilised as a food resource by people in northeastern Thailand and Laos (Kongim 2008: fig. 5), and large species are also consumed in India (Sajan et al. 2020; NA Aravind and B Páll-Gergely, pers. Comm.). Many cyclophorid genera are common, widespread, and species rich, and these snails serve as ecologically significant components of tropical habitats, i.e., the karstic landscapes in Indochina (Nantarat et al. 2014b, 2019; Foon et al. 2017; Oheimb et al. 2019). Their morphology is varied and they exhibit a wide range of sizes, from micro-snails with a maximum diameter of less than 5 mm to large species with diameters exceeding 30 mm. They occupy a wide range of habitats, from the forest floor under leaf litter to entirely arboreal settings (Phung et al. 2017).

More than 30 genera or subgenera of the cycophorids sensu stricto have been reported in Southeast Asia (e.g., Kobelt 1902; Gude 1921; van Benthem Jutting 1948; Egorov 2009). One of the most remarkable shell forms is displayed by the genus *Rhiostoma* Benson, 1860, which is also among the least studied cyclophorid groups. The snails in this genus have been classified into the pterocyclinid subfamily (Kobelt and Möllendorff 1897; Kobelt 1902, 1911; Wenz 1938; Vaught 1989); however, the most recent classification of the Gastropoda recognised this as the tribe Pterocyclini within the subfamily Cyclophorinae, which has been adopted in this study. This tribe consists of two additional genera, namely *Pterocyclos* Benson, 1832 and *Spiraculum* Pearson, 1833, and they possess a depressed or disc-shaped shell, wide umbilicus, peristome with incision or a breathing tube and calcified operculum, which are the typical characters that define the Pterocyclini (Kobelt 1902; Thiele 1929; Wenz 1938; Egorov 2009).

The genus *Rhiostoma*, the so-called “snorkel snails”, was nominated as a distinct genus from *Aulopoma* Troschel, 1847 and *Pterocyclos* based on its peculiar descending last whorl which is loose (detached whorl), with incision or tubular breathing device and calcareous, cup-shaped operculum. Benson (1860) also included three nominal species, namely *R.haughtoni* Benson, 1860 from Myanmar, *R.housei* (Haines, 1855) from Siam [Thailand], and *R.tener* (Menke, 1856) from Cochinchina [Vietnam] into this new genus. This classification was agreed upon by Blanford (1864), Pfeiffer (1865) and Martens (1867), and the genus was enlarged to include six species. However, without apparent evidence, Nevill (1878) and Fischer (1885) lowered the rank of *Rhiostoma* to a subgenus of *Pterocyclos*, but this was not widely accepted. Later in the systematic revision of the Cyclophoridae, Kobelt and Möllendorff (1897) raised *Rhiostoma* again to distinct genus level and then the genus was revised in Kobelt (1902, 1911). This has remained the accepted classification, and the literature reports ten species of this genus from Indochina, peninsular Malaysia, and Myanmar. Since then, no further modern systematic work has been published. Until the end of the twentieth century, only seven species were subsequently described and added to the genus, giving the genus a total of seventeen species (Blanford 1902; Sykes 1903; Bartsch 1932; Tomlin 1932, 1938; Salisbury 1949; Habe 1965). Interestingly, in the last two decades, twelve new *Rhiostoma* s. l. species have been described from Southeast Asia (i.e., Thach 2016, 2017, 2018, 2020; Do et al. 2020b).

Shells of the *Rhiostoma* are readily distinguished from other genera by the detached and descending portion of the last whorl with a distinctive peristomal breathing device attached and calcareous operculum (Benson 1860; Kobelt 1902; Tumpeesuwan 2001; Egorov 2009). This conspicuous snail is considered endemic to Southeast Asia, where it typically occurs in limestone habitats with high abundance; it is also present in non-limestone habitats but with less frequency. So far, the genus contains ~ 30 nominal species; however, the validity of several of these is debatable and they may represent ambiguous genus assignment (MolluscaBase 2022). Presumably, this taxonomic confusion arises not only because of the convergence of diagnostic characters, which also appears in other genera such as *Cyclotus* Swainson, 1840, *Opisthoporus* Pfeiffer, 1851, *Ptychopoma* Möllendorff, 1885 and *Pterocyclos*, but also because of overlapping geographical distribution. Therefore, the traditional diagnostic traits may inadequately distinguish or recognise species or genus-level categories. The soft part anatomy, especially of the reproductive organs, has been studied in a few cyclophoroidean groups (Sarasin and Sarasin 1899; Weber 1925; Tielecke 1940; Jonges 1980; Kongim et al. 2013a; Sutcharit et al. 2014; Páll-Gergely et al. 2020b). They show only slight variation and are not desirable for systematic study, in contrast to the highly variable and taxonomically informative ‘pulmonate’ genitalia. In addition, integrative approaches have been carried out in almost none of the cyclophorid genera to date. Species demarcation is poorly understood and will require extensive revision.

After the development of molecular systematics and DNA barcoding, this technique has promoted the rapid study of biodiversity and is often used in species identification. The DNA barcoding function (Hebert et al. 2003a, b, 2004; Hebert and Gregory 2005), especially, has been widely used in species delimitation and recognition in various groups of organisms where morphological characters have proven to be indistinguishable, i.e., the cyclophorid genera *Cyathopoma* Blanford & Blanford, 1861, *Cyclophorus* Montfort, 1810, *Cyclotus* and *Japonia* Gould, 1859 (Lee et al. 2008a, b, 2012; Nantarat et al. 2014a, b, 2019; Cole 2019; Cole et al. 2019). This study presents the first comprehensive taxonomic revision of all of the nominal species attributed to the *Rhiostoma* based on conchological characters, and includes illustrations of all type specimens (when available) and descriptions of the shells and radulae. We also analysed the mitochondrial cytochrome c oxidase subunit I (COI) gene phylogeny to test the monophyly of each species and better understand the phylogenetic relationships among the *Rhiostoma*. In addition, twelve *Rhiostoma* species from Thailand and Laos are herein described as new to science. The distribution ranges for all recognised species are updated.

## ﻿Materials and methods

### ﻿Specimen sampling

Areas were surveyed throughout Thailand, Laos, peninsular Malaysia, southern Cambodia, and southern Myanmar. Eighteen currently recognised species of the genus *Rhiostoma* were hand-collected mainly from limestone karst habitats, but also in non-karstic forests in several accessible localities to cover all the *Rhiostoma* species. Living snails were photographed when actively crawling and then preserved following the two-step method suggested by the AVMA Guidelines for the Euthanasia of Animals (AVMA 2020). Ethanol (95%, v/v) was used for tissue fixing, and then foot tissues were cut and preserved in 95% (v/v) ethanol at -20 °C until used for molecular studies. The remaining specimens were transferred to 70% (v/v) ethanol for specimen storage and future anatomical examination, and to serve as vouchers.

### ﻿DNA extraction and PCR amplification

Whole genomic DNA was extracted from the foot tissues using a NucleoSpin Tissue kit (MACHEREY-NAGEL, Germany), following the manufacturer’s standard protocol. Fragments of the cytochrome c oxidase subunit I (COI) mitochondrial gene was amplified and used to estimate molecular phylogeny. Primer sets used for polymerase chain reaction (PCR) and sequencing were either LCO1490 and HCO2198 (Folmer et al. 1994) or LoboF1 and LoboR1 (Lobo et al. 2013), depending on the specimen. The PCR amplification was conducted in a final volume of 20 µl containing 1 µl of each primer (10 mM), 10 ng of the extracted genomic DNA, 10 µl of EmeraldAmp PCR Master Mix (TAKARA BIO INC., Japan) and distilled water up to 20 μL total volume. Each PCR reaction was performed using a T100 thermal cycler (BIO-RAD, USA). The thermal cycling was started at 94 °C for 3 min, followed by 35 cycles of denaturation at 94 °C for 30 s, annealing at 50 °C (LCO1490 and HCO2198) or 46 °C (LoboF1 and LoboR1) for 60 s, extension at 72 °C for 90 s, then a final 72 °C for 5 min. All PCR products were purified by using a MicroSpin purification kit (Qiagen, USA) and then sent to BIONEER, Republic of Korea, for bi-directional sequencing on an automated sequencer (ABI Prism 3730XL). Nucleotide sequences were deposited in the GenBank database under GenBank submission numbers: OP491195 to OP491284 (Table 1).

**Table 1.** Samples and COI accession numbers for all sequences in this study.

Species / specimen code	CUMZ code	Locality and geographic coordinates	COI accession number
*Rhiostomaabletti* Thach, 2016			
KPL1	10206	Hot Springs, Meuang Hiam, Houaphanh, Laos (20°05'43.2"N 103°22'19.6"E)	OP491195
MRU22	10206	Hot Springs, Meuang Hiam, Houaphanh, Laos (20°05'43.2"N 103°22'19.6"E)	OP491196
PHL1	10207	Near Ban Na Puek, Meuang Hiam, Houaphanh, Laos (20°09'11.6"N, 103°24'21.6"E)	OP491197
*Rhiostomaasiphon* Möllendorff, 1893			
MRU16_1	4767	Koh Wua Talab, Koh Samui, Surat Thani (9°38'06.0"N, 99°40'11.8"E)	OP491198
MRU16_4	4767	Koh Wua Talab, Koh Samui, Surat Thani (9°38'06.0"N, 99°40'11.8"E)	OP491199
SS2	4756	Koh Sam Sao, Koh Samui, Surat Thani (9°39'32.0"N, 99°41'01.0"E)	OP491200
*Rhiostomabreviocollar* sp. nov.			
KB10	3975	Wat Khao Smokon, Ban Mi District, Lopburi (14°54'25.9"N, 100°30'21.9"E)	OP491201
KB11	3975	Wat Khao Smokon, Ban Mi District, Lopburi (14°54'25.9"N, 100°30'21.9"E)	OP491202
*Rhiostomacambodjense* (Morelet, 1875)			
MRU11_1	4714	Tham Khao Chakan, Khao Chakan, Sa Kaeo (13°39'37.8"N, 102°05'06.7"E)	OP491203
MRU11_2	4714	Tham Khao Chakan, Khao Chakan, Sa Kaeo (13°39'37.8"N, 102°05'06.7"E)	OP491204
MRU12_1	4714	Tham Khao Chakan, Khao Chakan, Sa Kaeo (13°39'37.8"N, 102°05'06.7"E)	OP491205
MRU12_2	4714	Tham Khao Chakan, Khao Chakan, Sa Kaeo (13°39'37.8"N, 102°05'06.7"E)	OP491206
*Rhiostomacheliopegma* sp. nov.			
CA	4886	Khao Cha-Ang, Bo Thong, Chonburi (13°12'29.8"N, 101°39'06.5"E)	OP491207
CA1	4886	Khao Cha-Ang Oun, Bo Thong, Chonburi (13°12'29.8"N, 101°39'06.5"E)	OP491208
CA11	4886	Khao Cha-Ang Oun, Bo Thong, Chonburi (13°12'29.8"N, 101°39'06.5"E)	OP491209
CA13	4886	Khao Cha-Ang Oun, Bo Thong, Chonburi (13°12'29.8"N, 101°39'06.5"E)	OP491210
CA2	4886	Khao Cha-Ang Oun, Bo Thong, Chonburi (13°12'29.8"N, 101°39'06.5"E)	OP491211
CA7	4886	Khao Cha-Ang Oun, Bo Thong, Chonburi (13°12'29.8"N, 101°39'06.5"E)	OP491212
R1	4818	Tham Khao Loy, Khao Chamao, Rayong (13°03'29.0"N, 101°36'24.9"E)	OP491213
R12	4818	Tham Khao Loy, Khao Chamao, Rayong (13°03'29.0"N, 101°36'24.9"E)	OP491214
R13	4818	Tham Khao Loy, Khao Chamao, Rayong (13°03'29.0"N, 101°36'24.9"E)	OP491215
R4	4860	Tham Khao Prathun, Bo Thong, Chonburi (12°57'43.24"N, 101°01'11.44"E)	OP491216
TT4	3985/2	Tham Takein, Khao Chamao, Rayong (12°56'49.27"N, 101°42'34.01"E)	OP491217
TT5	3985/2	Tham Takein, Khao Chamao, Rayong (12°56'49.27"N, 101°42'34.01"E)	OP491218
*Rhiostomadalyi* Blanford, 1903			
AT1	3979	Hill Near Tham Air Thammachart, Long, Phrae (18°17'16.6"N, 100°00'43.8"E)	OP491219
AT2	3979	Hill Near Tham Air Thammachart, Long, Phrae (18°17'16.6"N, 100°00'43.8"E)	OP491220
AT6	3979	Hill Near Tham Air Thammachart, Long, Phrae (18°17'16.6"N, 100°00'43.8"E)	OP491221
AT7	3979	Hill Near Tham Air Thammachart, Long, Phrae (18°17'16.6"N, 100°00'43.8"E)	OP491222
AT9	3979	Hill Near Tham Air Thammachart, Long, Phrae (18°17'16.6"N, 100°00'43.8"E)	OP491223
AT15	3979	Hill Near Tham Air Thammachart, Long, Phrae (18°17'16.6"N, 100°00'43.8"E)	OP491224
AT16	3979	Tham Air Thammachart, Long, Phrae (18°17'16.6"N, 100°00'43.8"E)	OP491225
*Rhiostomafurfurosum* sp. nov.			
NP1	3901	Noen Maprang, Phitsanulok (16°41'37.9"N, 100°40'44.9"E)	OP491226
NP3	3901	Noen Maprang, Phitsanulok (16°41'37.9"N, 100°40'44.9"E)	OP491227
NP4	3901	Noen Maprang, Phitsanulok (16°41'37.9"N, 100°40'44.9"E)	OP491228
NP16	3901	Noen Maprang, Phitsanulok (16°41'37.9"N, 100°40'44.9"E)	OP491229
NP18	3901	Noen Maprang, Phitsanulok (16°41'37.9"N, 100°40'44.9"E)	OP491230
NPA2	3901	Noen Maprang, Phitsanulok (16°41'37.9"N, 100°40'44.9"E)	OP491231
NPB1	3901	Noen Maprang, Phitsanulok (16°41'37.9"N, 100°40'44.9"E)	OP491232
NPB2	3901	Noen Maprang, Phitsanulok (16°41'37.9"N, 100°40'44.9"E)	OP491233
*Rhiostomahainesi* Pfeiffer, 1862			
MRU8	4814	Makok Waterfall, Khlung, Chanthaburi (12°35'12.0"N, 102°15'21.0"E)	OP491234
SD	4457	Khao Soi Dao Breeding Centre, Pong Nam Ron, Chanthaburi (12°55'19.8"N, 102°14'39.7"E)	OP491235
*Rhiostomahaughtoni* Benson, 1860			
MRU4	10048	Dhammatat Cave, Mawlamyine, Mon, Myanmar (16°30'04.9"N, 97°49'16.6"E)	OP491236
*Rhiostomahousei* (Haines, 1855)			
KN	4352	Khao Noi, Nakorn Sawan (15°38'57"N, 100°28'35"E)	OP491237
PV	4772	Khao Patavi, Tubtan, Uthai Thani (15°28'29.5"N, 99°45'25.4"E)	OP491238
PT14	4772	Khao Patavi, Tubtan, Uthai Thani (15°28'29.5"N, 99°45'25.4"E)	OP491239
LM2	3987	Khao Lom Muak, Muang, Prachuap Khiri Khan (11°47'04.0"N, 99°48'53.9"E)	OP491240
TD2	10133	Tham Dao Khao Keaw, Muak Lek, Saraburi (14°52'31.0"N, 101°20'16.0"E)	OP491241
SL1	3982	Wat Tham Srivilai, Chaloem Phra Kiat, Saraburi (14°42'43.9"N, 100°51'58.6"E)	OP491242
SL2	3982	Wat Tham Srivilai, Chaloem Phra Kiat, Saraburi (14°42'43.9"N, 100°51'58.6"E)	OP491243
*Rhiostomajalorensis* Sykes, 1902			
MRU7	3819	Tham Sra Yoon Thong, Ao Luek, Krabi (8°23'36.6"N, 98°46'24.8"E)	OP491244
MRU13_2	10146	Tharn Bok Khorani, Ao Luek, Krabi (8°23'18.0"N, 98°44'04.0"E)	OP491245
TO	4483	Tao Thong Waterfall, Thap Put, Phang Nga (8°29'08.0"N, 98°35'07.0"E)	OP491246
MRU10	4843	Wat Tham Suwannakhuha, Takua Thung, Phang Nga (8°25'42.3"N 98°28'20.4"E)	OP491247
MRU14	3394	Wat Tham Sua, Muang, Krabi (8°07'37.2"N, 98°55'27.3"E)	OP491248
Nampud	4387	Tham Nam Pud, Muang, Phang Nga (8°27'51.1"N, 98°32'30.3"E)	OP491249
RTK2017	10143	Tham Kob, Thap Put, Phang Nga (8°31'59.3"N, 98°34'39.9"E)	OP491250
*Rhiostomalannaense* sp. nov			
MRU1	4701	Ban Ping Klong (village), Chiangdao, Chiang Mai (19°30'48.6"N, 99°03'21.1"E)	OP491251
MRU2	4701	Ban Ping Klong (village), Chiangdao, Chiang Mai (19°30'48.6"N, 99°03'21.1"E)	OP491252
MRU3_1	4702	Tham Mae Lana, Pang Mapha, Maehongsorn (19°34'13.4"N, 98°12'04.4"E)	OP491253
MRU3_2	4702	Tham Mae Lana, Pang Mapha, Maehongsorn (19°34'13.4"N, 98°12'04.4"E)	OP491254
MRU26_1	10043	Tham Mae Lana, Pang Mapha, Maehongsorn (19°34'13.4"N, 98°12'04.4"E)	OP491255
MRU26_2	10043	Tham Mae Lana, Pang Mapha, Maehongsorn (19°34'13.4"N, 98°12'04.4"E)	OP491256
*Rhiostomamorleti* Dautzenberg & Fischer, 1906			
SHL1	1002/1	Kraisorn Cave, Vieng Xai, Houaphanh, Laos (20°23'22.4"N, 104°13'41.4"E)	OP491257
UHL1	1004/2	Ban Na Wid, Vieng Xai, Houaphanh, Laos (20°26'59.5"N, 104°10'51.2"E)	OP491258
*Rhiostomaplatymorpha* sp. nov			
MO22	4763	Tham Muang On, Mae On, Chiang Mai (18°47'10.6"N, 99°14'17.1"E)	OP491259
MO23	4763	Tham Muang On, Mae On, Chiang Mai (18°47'10.6"N, 99°14'17.1"E)	OP491260
MO46	4763	Tham Muang On, Mae On, Chiang Mai (18°47'10.6"N, 99°14'17.1"E)	OP491261
MO47	4763	Tham Muang On, Mae On, Chiang Mai (18°47'10.6"N, 99°14'17.1"E)	OP491262
MO48	4763	Tham Muang On, Mae On, Chiang Mai (18°47'10.6"N, 99°14'17.1"E)	OP491263
*Rhiostomarhothonotaphrosa* sp. nov			
RST	3858	Tham Sri Thong, Klong Hat, Sa Kaeo (13°28'43.6"N, 102°16'53.8"E)	OP491264
MRU17_2	10172	Tham Phet Pho Thong, Klong Hat, Sa Kaeo (13°24'49.0"N, 102°19'31.0"E)	OP491265
MRU17_3	10172	Tham Phet Pho Thong, Klong Hat, Sa Kaeo (13°24'49.0"N, 102°19'31.0"E)	OP491266
*Rhiostomasamuiense* Tomlin, 1932			
CP	3996	Bukit Chuping, Kangar, Perlis, Malaysia (6°29'44.0"N, 100°15'56.3"E)	OP491267
HH	3840	Khao Huay Hang, Huay Yod, Trang (7°47'38.6"N, 99°38'38.5"E)	OP491268
KK	10077	Khao Pu-KhaoYa, Si Banphot, Phatthalung (7°40'37.0"N, 99°52'35.0"E)	OP491269
KT	4707	Koh Tan, Koh Samui, Surat Thani (9°22'17.0"N, 99°57'06.0"E)	OP491270
MRU6	4774	Khao Huay Hang, Huay Yod, Trang (7°47'38.6"N, 99°38'38.5"E)	OP491271
SP	3997	Sungai Jernih, Kangar, Perlis, Malaysia (6°32'49.6"N, 100°16'08.6"E)	OP491272
WT	4708	Tham Wang Thong, Kanom, Nakhon Sri Thamarat (9°12'16.0"N, 99°46'26.6"E)	OP491273
*Rhiostomasimplicilabre* Pfeiffer, 1862			
MRU18_1	4868	Sapan Waterfall, Bo Kluea, Nan (19°11'25.0"N, 101°11'55.0"E)	OP491274
MRU18_2	4868	Sapan Waterfall, Bo Kluea, Nan (19°11'25.0"N, 101°11'55.0"E)	OP491275
MRU18_3	4868	Sapan Waterfall, Bo Kluea, Nan (19°11'25.0"N, 101°11'55.0"E)	OP491276
*Rhiostomatigrina* sp. nov			
MRU31	10193	Tham Saohin Prayanak, Mae Sai, Chiang Rai (20°19'24.6"N, 99°51'51.5"E)	OP491277
MRU31_2	10193	Tham Saohin Prayanak, Mae Sai, Chiang Rai (20°19'24.6"N, 99°51'51.5"E)	OP491278
MRU34	3909	Wat Tham Pum Tham Pla, Mae Sai, Chiang Rai (20°20'53.2"N, 99°51'29.3"E)	OP491279
*Pterocyclosblandi* (Benson, 1851)			
TU	4582	Teluic Ewa, Langkawi, Kedah, Malaysia (6°25'02.0"N, 99°45'44.0"E)	OP491280
*Pterocyclosdiluvium* Sutcharit & Panha, 2014			
19PK	3812	Tam Puttha Kodome, Srinagarindra, Patthalung (7°33'36.5"N, 99°53'10.5"E)	OP491281
TS	4588	Tham Sumano, Srinagarindra, Patthalung (7°35'12.0"N, 99°52'04.0"E)	OP491282
GU	4592	Gua Cenderawasih, Kangar, Perlis, Malaysia (6°24'45.8"N, 100°11'33.7"E)	OP491283
*Ptychopomaperrieri* (Morlet, 1889)			
RMK		Makok Waterfall, Khlung, Chanthaburi (12°35'12.0"N, 102°15'21.0"E)	OP491284

### ﻿Phylogenetic analysis

Eighty-five specimens of *Rhiostoma* species were included as an ingroup in phylogenetic analysis. The ingroups were subsampled from the specimens used in the morphological studies. These samples were from eleven described species and seven recently described species of *Rhiostoma*. Collection localities and coordination details of the DNA specimens are listed in Table 1. Because the phylogenetic relationships among the Southeast Asian Cyclophoridae have been poorly explored, except in *Cyclophorus* (Nantarat et al. 2014b, 2019; Oheimb et al. 2019), three outgroup species were selected from possible closely related genera in the same subfamily Cyclophorinae. These outgroups were *Pterocyclosblandi* (Benson, 1851), *Pterocyclosdiluvium* Sutcharit & Panha, 2014, and *Ptychopomaperrieri* (Morlet, 1889).

Sequences were aligned and edited using ClustalW as implemented in MEGA 7.0.26 (Kumar et al. 2016). Phylogenetic trees were reconstructed using maximum likelihood (ML) in the program RAxML v. 8.2.10 (Stamatakis 2014) and Bayesian inference (BI) in the program MrBayes v. 3.2.6 (Ronquist et al. 2012) through the on-line CIPRES Science Gateway (Miller et al. 2010). The ML analysis was conducted using 1,000 ML bootstrap replications and GTRGAMMA as the model for all gene partitions. The BI analysis was performed with the Markov chain Monte Carlo analysis (MCMC) in two parallel runs and with four chains each. The best-fit nucleotide substitution models were set as GTR+I+G, HKY+G, and GTR+G for the first, second, and third codon positions of COI, respectively, as suggested by PartitionFinder2 v. 2.3.4 (Lanfear et al. 2016) based on the Akaike Information Criterion (AIC: Akaike 1974). The MCMC were run using random starting tree for 10 million generations and tree sampling every 1000^th^ generation. The first 25% of obtained trees were discarded as burn-in. The remaining trees were used to estimate the consensus tree topology, bipartition posterior probability (bpp), and branch length. The adequate sample size (ESS) value sampled from the MCMC analysis was greater than 1,200 in all parameters. For the clade supports, nodes with ML bootstrap values (bs) of 70% or greater and/or BI bipartition posterior probability of 0.95 or greater were regarded as sufficiently resolved (Huelsenbeck and Hillis 1993; Larget and Simon 1999).

Genetic divergence was also calculated to depict evolutionary divergence between the *Rhiostoma* species and related taxa using uncorrected p-distances as implemented in MEGA7 (Kumar et al. 2016).

### ﻿Morphological studies

**Figure 1.**
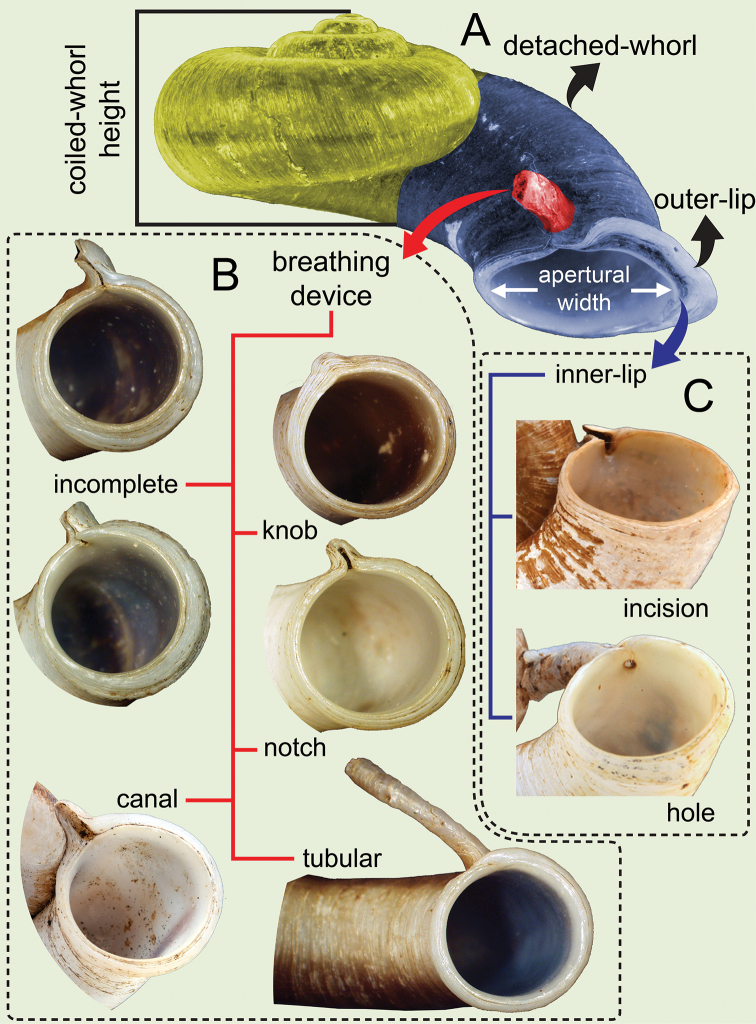
Schematic of shell morphology and terminology of the *Rhiostoma***A** apertural view with shell terminology and measurements of coiled-whorl height **B** breathing device types as recognised in this revision: knob, incomplete tube, notch, canal and tubular **C** inner peristomal lip variation as recognised in this revision: incision and hole.

**Figure 2.**
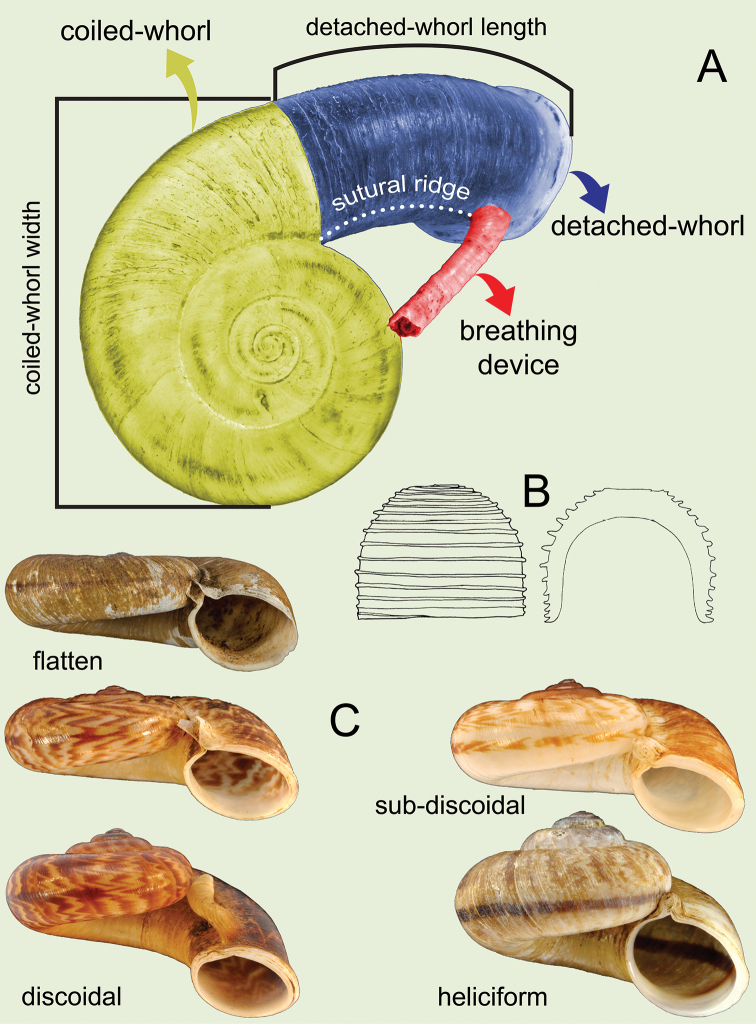
Schematic of shell morphology and terminology of the *Rhiostoma***A** apical view with shell terminology and measurements of coiled-whorl width and detached-whorl length **B** operculum showing side and cross-section views **C** apertural views showing various shell shapes as recognised in this revision: flattened, discoidal, sub-discoidal and heliciform.

The empty shells and preserved specimens were initially identified based on literature: Haines (1855), Pfeiffer (1857, 1862a), Benson (1860), Morelet (1875), Möllendorff (1893, 1899), Ancey (1898), Blanford (1902), Kobelt (1902, 1911–1914), Sykes (1903), Dautzenberg and Fischer (1906), Bavay and Dautzenberg (1909a, b), Gude (1921), and Tomlin (1932, 1938), and then confirmed by comparison with the relevant type specimens. The adult specimens were measured using an Absolute Digimatic Caliper, model No. CD-15CP, from Mitutoyo Corp., with a margin of error of 0.02 mm. Two dimensions, shell height (**H**) and shell width (**W**), were measured for the taxa considered to have no detached whorl. For the taxa that possessed a short to long detached whorl, the measurements were modified to avoid the variation and allometric effect of the detached whorl. Therefore, dimensions were measured only on the coiled-whorl portion, as demonstrated in Figs 1A, 2A: height (**cH** = maximum dimension of coiled-whorl height parallel to the columellar axis) and width (**cW** = maximum dimension of coiled-whorl width perpendicular to the columellar axis). In addition, the detached-whorl length (maximum length of detached whorl to apertural lip) and aperture width (maximum dimension of inner aperture) were also measured (Figs 1A, 2A). The detached-whorl length/aperture width ratio was calculated and used as a descriptive term for detached-whorl length: short (detached-whorl length/aperture width ratio less than 1), medium (detached-whorl length/aperture width ratio between 1 and 2), and long (detached-whorl length/aperture width ratio greater than 2).

Male and/or female reproductive organs (2–5 specimens) of recognised species were examined, and only two species, namely *R.haughtoni* Benson, 1860 (type species) and *R.housei* (Haines, 1855), have been carefully described herein. Radulae were extracted, soaked in 10% sodium hydroxide, cleaned in distilled water, and examined under a scanning electron microscope (JEOL, JSM-5410 LV); teeth formulae and morphology were also described.

### ﻿Nomenclature and description of shell characters

The measurements of shell dimensions and counts of whorls of many *Rhiostoma* species are inconsistent due to the high variation of the detached part of the last whorl. Therefore, we separate the *Rhiostoma* shell into coiled-whorl and detached-whorl portions. In addition, breathing devices, whorls and operculum shape are always different among congeners and other related genera (Figs 1, 2). Therefore, we have described these taxonomically informative characters as follows:

**Coiled whorl**: This refers to the coiling part of the shell starting from the protoconch to the onset of the detached whorl. Whorl numbers are counted only on the coiled whorl, and cH and cW are measured only on the coiled whorl to calculate shell dimensions (Figs 1A, 2A).

**Detached whorl**: There are few species of *Rhiostoma* that have regular coiling, where all whorls are attached. However, in many species the terminal part of the last whorl is free or disconnected from the preceding whorl. This detached whorl can be short to long, descending and twisted, which turns the aperture to open ventrally. Among the species examined herein, the detached whorl tends to vary greatly among populations of *R.samuiense* Tomlim, 1932 and *R.jalorensis* Sykes, 1903. The COI barcoding suggests these variations are conspecific.

The length of the detached whorl was measured from the onset of detached whorl to the apertural lip (Fig. 2A). These measurements were used to differentiate the length of detached whorl as either short (equivalent to or shorter than apertural width), medium (< 2× longer than apertural width), or long (≥ 2× longer than aperture width).

**Peristome**: The peristome shape is generally circular, and in young specimens, the peristome tends to be simple with a thin and sharpened lip and without a breathing device. The breathing device originates when the snail becomes an adult and is much thicker in older snails. The peristome is circular, continuous, and double, multi-layered (inner and/or outer peristomes multi-layered) or sometimes with the boundary hardly visible; and apertural lip is thick or thin and slightly to broadly expanded and multi-layered. At the upper junction of the peristome or on the sutural ridge, there are always various types of breathing structures, here called the “breathing device”. Gratacap (1907) and Rees (1964) reviewed the breathing devices in various cyclophorid taxa, and their descriptions are followed herein with some modification. In the *Rhiostoma*, the breathing device structure tends to develop from simple and inconspicuous to highly developed and with very distinctive traits. This character could be used as one of several diagnostic characters to recognise species (Fig. 1).

**Breathing device**: This character denotes the modification of the peristome into five types of breathing devices, some which may not be functional. The genus *Rhiostoma* tend to exhibit a unique structure of breathing device among pterocyclinid genera. The location of this breathing device is consistent for all nominal species; it is located on the upper junction of the peristome. The knob, notch, and canal shapes are situated on the peristomal lip, which is interrupted with incision or expansion. Because the incomplete tube and tubular shapes are generally located just behind the thickened lip, the apertural lip is generally continuous without incision (Fig. 1B, C). However, the early stage in the development of these two types might be similar, but it is clearly distinct in older specimens. There are five different types of breathing devices found among *Rhiostoma* species. In the specimens we examined, each species had only one type of device, with the exception of *R.hainesi* Pfeiffer, 1862, which had three. The five types of breathing devices can be recognised as:

***Knob shape***: This is the simplest type of breathing device (Fig. 1B). The outer lip (outer peristome lip) is curved or with a small protrusion located at the ridge adherent to the preceding whorl, and the inner lip (inner peristome lip) is with an indistinct incision. This protruded knob is generally without a groove.

***Notch shape***: This is similar to the knob shape (Fig. 1B) but the outer lip is always larger and taller than the inner lip, with narrow to wide grooves, and the inner lip has a distinct incision (Fig. 1C).

***Canal shape***: This type of breathing device occurs in species without a detached whorl. The upper junction of the peristome is expanded with a triangular shape, projected forward and attached to the preceding whorl (Fig. 1B). The outer lip is expanded at the base, with a wide canal or groove, and the inner lip is with shallow to deep incision or without prominent incision (inner lip continuous).

***Incomplete tube***: The incomplete tube (= channel-like or short tubular in Laidlaw (1939)) in is generally shorter than in the tubular form and situated on the peristomal lip or just behind the thickened lip (Fig. 1B). The outer lip is thickened, slightly expanded and forms a nearly closed tube. This tube always has a narrow slit along the tube length, and it is connected to the incision on the inner lip. The inner lip has a deep incision, or a small hole opening into the shell (Fig. 1C). Thus, the apertural lip is interrupted with a vertical slit continuing from the tube. At an early stage of its formation, this incomplete tube may be similar to the notch shape, and older specimens may have peristomal lip fused.

***Tubular***: This complete tube is developed only when the snail reaches the adult stage (Fig. 1B). This tube is sometimes similar to an incomplete tube at the early stage. The outer lip is slightly expanded, rolled and forms a short to a long cylindrical tube that is just behind the thickened lip, with a small hole opening into the shell. The inner lip has a small hole located inside the shell (Fig. 1C). The apertural lip is continuous and without a slit. However, there is some trace of a slit-like scar on the peristome and tube, which has been resealed together and followed by a thickening of the apertural lip. This tubular breathing device can vary from being curved upwards or backwards, and as a short tube or long tube which is long enough to touch the wall of the preceding whorl.

**Operculum**: The operculum of *Rhiostoma* is characterised by thick calcareous deposits with a low to tall cup shape (Figs 2B, 5; dome shape or dorsal side generally flat), and considerably smaller diameter and tightly fitting inside the aperture. The outer surface with anticlockwise and closely to loosely coiled multispiral with elevated lamella that results in spiral layers of calcareous lamellae. The opercula of young specimens tend to have a thin plate shape, slightly concave inside, and usually are covered with thick brownish periostracum outside. When the snail becomes adult, the operculum becomes thicker and taller, and periostracum may be eroded; in older specimens, the elevated lamellae might be cracked or eroded at the centre. The inner side of the operculum is generally concave, shallow to deep with a smooth surface, and attached to the dorsal side of the posterior foot. All of the *Rhiostoma* species recognised in this revision share common characters of the operculum (i.e., calcareous, cup-shaped, and multispiral with elevated lamella), and these characters are distinct from the other cyclophorinids, making this useful for generic recognition (Fig. 5, Table 2). Furthermore, in some cases, opercular features may be useful diagnostic characters for species recognition.

**Table 2.** Comparative shell morphology among six genera that are superficially similar to *Rhiostoma*. The characters are mainly based on the type species. The tribe classification for each genus follows Bouchet et al. (2017). References: ^1^Benson (1860) ; ^2^Möllendorff (1885), Kobelt (1906) and Yen (1939); ^3^Kobelt (1902), Thiele (1929), Wenz (1938) and Egorov (2009); ^4^Kobelt (1912), and Low and Tan (2017); ^5^Gude (1921) and Kobelt (1911).

		Pterocyclini			Cyclotini		Cyclophorini
		*Pterocyclos* Benson, 1832^3,5^	*Spiraculum* Pearson, 1833^3,5^	*Rhiostoma* Benson, 1860^1,3,5^	*Cyclotus* Swainson, 1840^3,5^	*Opisthoporus* Benson in Pfeiffer, 1851^3,4^	*Ptychopoma* Möllendorff, 1885^2,3^
**Type species**		*Pterocyclosrupestris* Benson, 1832	*Spiraculumhispidum* Pearson, 1833	*Rhiostomahaughtoni* Benson, 1860	*Cyclotusvariegatus* Pearson, 1833	*Cyclostomataylorianus* Pfeiffer, 1851^5^	*Cyclophoruschinensis* Möllendorff, 1874
**Shell shape**		discoidal	discoidal	discoidal	discoidal to turbinate	discoidal	discoidal
**Periostracum**		thick to thin corneous	thick corneous and hairy	thin corneous	thin corneous	thick corneous and hairy	thick corneous
**Detached whorl**		absent to short	absent*	absent to extremely long (curved and descending)	absent to short	absent to short	absent
**Breathing device**:	**sutural tube**	absent	present	absent	absent**	absent	absent
	**upper junction of peristome**	expanded, thickened and free or attached to preceding whorl	expanded and attached to preceding whorl	thickened, free or attached to preceding whorl	expanded and free or attached to preceding whorl	expanded and free or attached to preceding whorl	thickened and attached to preceding whorl
**Outer peristome**		widely expanded with wing or hood	expanded with wing or hood	thickened with knob, notch or tube	expanded	expanded with tube located close to lip	inconspicuous
**Inner peristome**		shallow to deep incision	shallow to deep incision	shallow to deep incision	shallow incision	shallow incision	inconspicuous
**Operculum**		calcareous (thin), concave to low cup-shaped with elevated lamella, lateral fringe straight	calcareous (thin), concave to low cup-shaped with elevated lamella, lateral fringe straight	calcareous (thick), tall to low cup-shaped with elevated lamella, lateral fringe straight	calcareous, concave without elevated lamella, lateral fringe with deep groove	calcareous, concave without elevated lamella, lateral fringe with deep groove	corneous, plate-shaped with low lamella
**Distribution**		South Asia to southern China and Indochina	South Asia to Indochina	principally in Indochina	Asia to Australia	principally in Indochina	Southern China to Japan

* *Spiraculummastersi* Blanford, 1877 from India have a short, detached whorl (see Sutcharit et al. 2019: fig. 9a). ** *Cyclotusbirostris* (Pfeiffer, 1855) from Borneo have sutural tube projecting anteriorly (see Sutcharit et al. 2019: fig. 2j, k).

### ﻿Anatomical and measurement abbreviations

The following anatomical terms are adapted from Schneider (1922), Weber (1925) and Sutcharit et al. (2014):

**an** anus

**bc** bursa copulatrix

**cm** columellar muscle

**ct** cephalic tentacle

**dg** digestive gland

**es** eyespot

**f** foot

**fc** faeces

**go** genital opening

**h** heart

**int** intestine

**kd** kidney

**lc** lung cavity

**mc** mantle collar

**od** oviduct

**op** operculum

**ov** ovary

**p** external penis

**pg** prostate gland

**re** rectum

**sg** sperm groove

**sn** snout

**sr** seminal receptacle

**sto** stomach

**te** testis

**ut** uterus

**v** vein

**vd** vas deferens

**vg** vaginal groove.

For further detail of the following measurement terms see Morphological studies (above):

**cH** coiled-whorl height

**cW** coiled-whorl width

**dL** detached-whorl length

**H** shell height

**W** shell width

### ﻿Institutional abbreviations

The type materials and examined specimens from museum collections are as follows:

**AMNH** American Museum of Natural History, New York

**BOR/MOL** The BORNEENSIS collections of Universiti Malaysia Sabah, Sabah

**CUMZ** Chulalongkorn University, Museum of Zoology, Bangkok

**FMNH** The Field Museum of Natural History, Chicago

**MNHN** Muséum National ďHistoire Naturelle, Paris

**NHMUK** The Natural History Museum, London

**NHMW** Naturhistorishes Museum, Wein, Austria

**NMNH** National Museum of Natural History, Smithsonian Institute, Washington D.C.

**NMW** National Museum of Well, Cardiff

**NSMT** National Science Museum and Technology, Tokyo

**RBINS** Royal Belgian Institute of Natural Sciences, Brussels

**SMF** Forschungsinstitut und Naturmuseum Senckenberg, Frankfurt, a.m.

**UMZC** University Museum of Zoology Cambridge, Cambridge

**UO** University of Oregon, Museum of Natural and Cultural History, Oregon

**ZMA** Zoological Museum, Amsterdam

**ZMUC** Zoological Museum, University of Copenhagen, København

### ﻿Photograph credits

Photographs of the type specimens and specimens from the museum collections are credited to each respective museum. One exception is the photographs of the type specimens from the MNHN-IM collection, which are credited to the museum taken under project E-RECOLNAT: ANR-11-INBS-0004.

### ﻿Taxon names

Descriptions of the new species are here attributed to different authors. Tongkerd and Panha are responsible for R. ? amarapuraense sp. nov., *R.ebenozostera* sp. nov., *R.furfurosum* sp. nov., *R.gnomus* sp. nov., and *R.rhothonotaphrosa* sp. nov. Tongkerd and Tumpeesuwan are responsible for *R.breviocollar* sp. nov., *R.cheliopegma* sp. nov., *R.lannaense* sp. nov., *R.platymorpha* sp. nov., and *R.tigrina* sp. nov. Tongkerd and Inkhavilay are responsible for *R.anceyi* sp. nov., and *R.laosense* sp. nov. Thus, complete citations of the authors are, respectively, Tongkerd and Panha in Tongkerd et al., Tongkerd and Tumpeesuwan in Tongkerd et al., and Tongkerd and Inkhavilay in Tongkerd et al.

## ﻿Results

### ﻿Phylogenetic analysis

**Figure 3.**
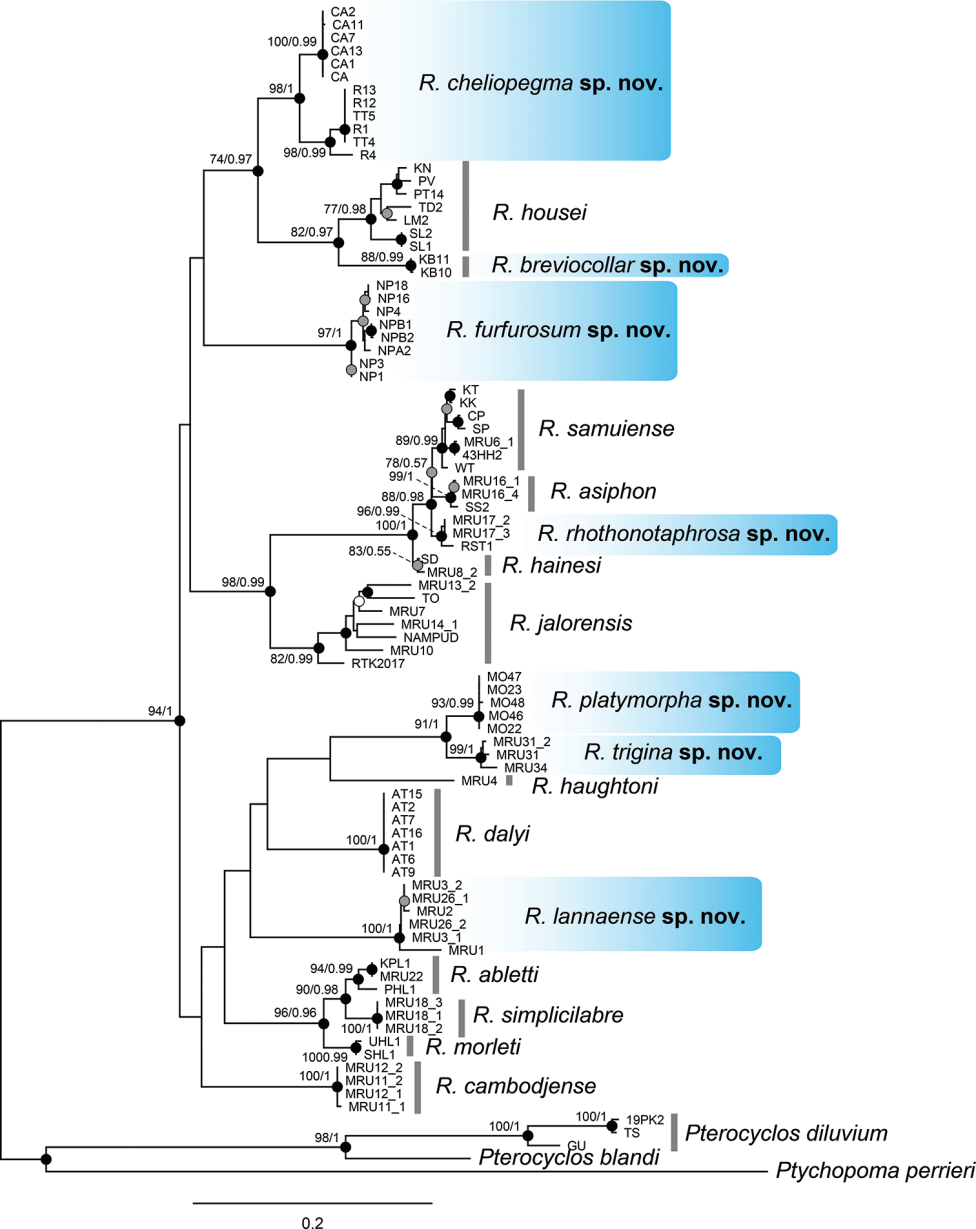
Maximum likelihood (ML) tree showing the relationships among *Rhiostoma* species based on 660 bp of the mitochondrial COI gene sequence. The major nodes are indicated by numbers as the Maximum likelihood (ML) bootstrap values and Bayesian posterior probabilities of Bayesian inference (BI), respectively. Nodes with sufficient support by both ML and BI are indicated by black circles, and nodes with partial support by only ML are indicated by grey circles, and only BI by white circles. Newly described species in this study are highlighted in blue.

The DNA phylogeny is implemented here to help determine monophyly and delimit species diversity within the genus *Rhiostoma*. The partial mitochondrial COI gene sequence data were obtained from 85 specimens of *Rhiostoma*, and five additional sequences from *Pterocyclos* and *Ptychopoma* species were included as outgroups. The alignment of the COI gene fragments of ingroups and outgroups had a length of 660 base pairs. A total of 289 (43.79%) variable sites were identified, of which 262 (39.70%) were parsimony informative. The monophyly of the *Rhiostoma* is strongly supported in all analyses (bpp = 1, bs = 94%; Fig. 3), confirming that all pterocyclinid snails with calcareous, cup-shaped operculum belong to the same clade.

Within the ingroups, phylogenetic analyses based on the COI gene data recovered eighteen monophyletic groups with strong support from both analyses, except for *R.hainesi*, which was supported only by ML (bpp = 0.55, bs = 83%; Fig. 3). These eighteen clades were then interpreted as distinct at species level and corresponded to eleven previously recognised species, whereas the remaining seven were considered undescribed species.

Although all of the recognised *Rhiostoma* species were retrieved as monophyletic separately, “*R.chupingense* Tomlin, 1938” (specimens CP, HH, KK, MRU6, SP, and WT in Fig. 3) was nested within the *R.samuiense* clade. This rendered a non-monophyletic relationship in both species, and in fact, *R.chupingense* was recovered as paraphyletic (Fig. 3). This was discordant with the previous morphologically based classification, since the shell morphology of the two species are obviously different. However, the species are distributed in close proximity to each other. The former report for *R.samuiense* is only from Samui Island, Gulf of Thailand, and reports for “*R.chupingense*” are from many localities on the Malay Peninsula. Morphological variation between them may be a result of local adaptation to different environments (Samui Island is a granitic island, whereas the mainland populations are from limestone hills). Therefore, in this study, we treat “*R.chupingense*” as a junior synonym of *R.samuiense* (see more details in the taxonomic section below).

Despite the poorly resolved groupings in the deeper nodes, some interesting evolutionary relationships can still be seen. These were a cluster of *R.simplicilabre*, *R.abletti*, and *R.morleti*; a cluster of *R.cheliopegma* sp. nov., *R.housei*, and *R.breviocollar* sp. nov.; and a sister relationship between *R.platymorpha* sp. nov. and *R.trigina* sp. nov. Interestingly, *R.samuiense* (including “*R.chupingense*”) was recovered as a sister lineage to a clade containing *R.asiphon*, *R hainesi* and *R.rhothonotaphrosa* sp. nov. However, the phylogenetic relationships among these taxa were poorly resolved and with shallow genetic divergence.

**Figure 4.**
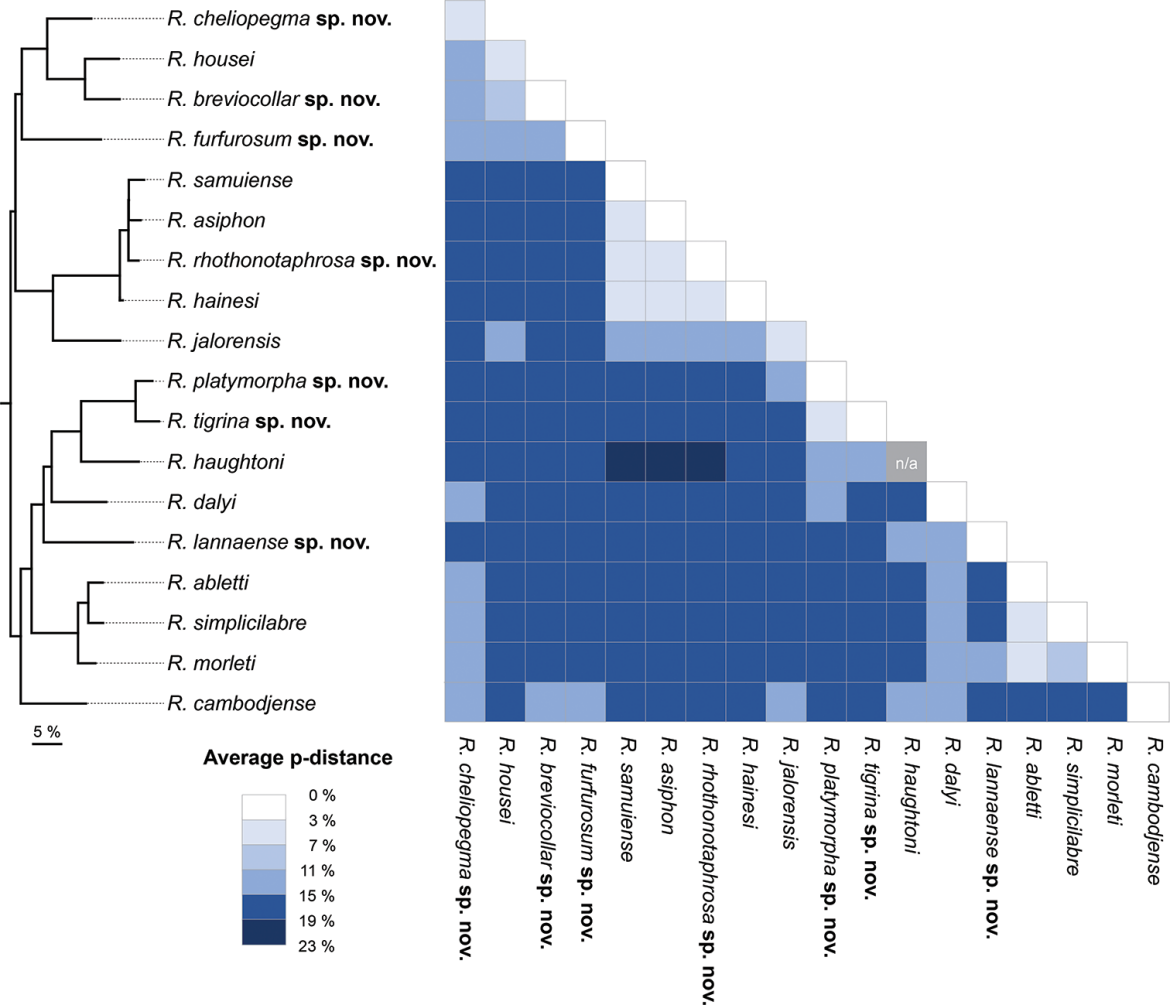
A heat map of average interspecific pairwise p-distance based on 660-bp COI gene fragment sequences between *Rhiostoma* species. The simplified phylogenetic tree was generated from the Maximum likelihood tree.

Intra/interspecific genetic distances based on uncorrected p-distance of the COI gene among the *Rhiostoma* species and outgroups are summarised in Fig. 4 and Appendix 1: Table A1. Among the eighteen *Rhiostoma* lineages, the average uncorrected p-distance of the COI gene ranged from 3.30% to 19.32% (average = 15.34%). The intraspecific genetic distances within each *Rhiostoma* lineage ranged from 0% to 6.69% (average = 1.43%). Two species showed relatively high intraspecific distance over the lowest interspecific distance (3.30%), namely *R.jalorensis* (6.69%) and *R.housei* (3.67%). In addition, the average genetic distance between *Rhiostoma* and *Pterocyclos* was 21.47%, between *Rhiostoma* and *Ptychopoma* was 21.48%, and between *Pterocyclos* and *Ptychopoma* was 22.14%.

### ﻿Systematics

#### Family Cyclophoridae Gray, 1847

**Subfamily Cyclophorinae Gray, 1847**

**Tribe Pterocyclini Kobelt & Möllendorff, 1897**

##### ﻿Genus Rhiostoma

Taxon classificationAnimaliaArchitaenioglossaCyclophoridae

Benson, 1860

1CD07730-1DDB-5B59-AE59-12B4EAD8F384

Rhiostoma Benson, 1860: 96. Pfeiffer 1862b: 45, 46. Blanford 1864: 451. Pfeiffer 1865: 38. Martens 1867: 63. Kobelt and Möllendorff 1897: 115. Kobelt 1902: 176. Kobelt 1911: 754, 755. Gude 1921: 127, 128. Thiele 1929: 100. Wenz 1938: 462. Egorov 2009: 36, 37. Preece et al. 2022: 247.
Pterocyclus [sic] (Rhiostoma)—Nevill 1878: 262. Fischer 1885: 745.

###### Type species.

*Rhiostomahaughtoni* Benson, 1860 by original designation in Benson (1860: 96).

###### Diagnosis.

**Figure 5.**
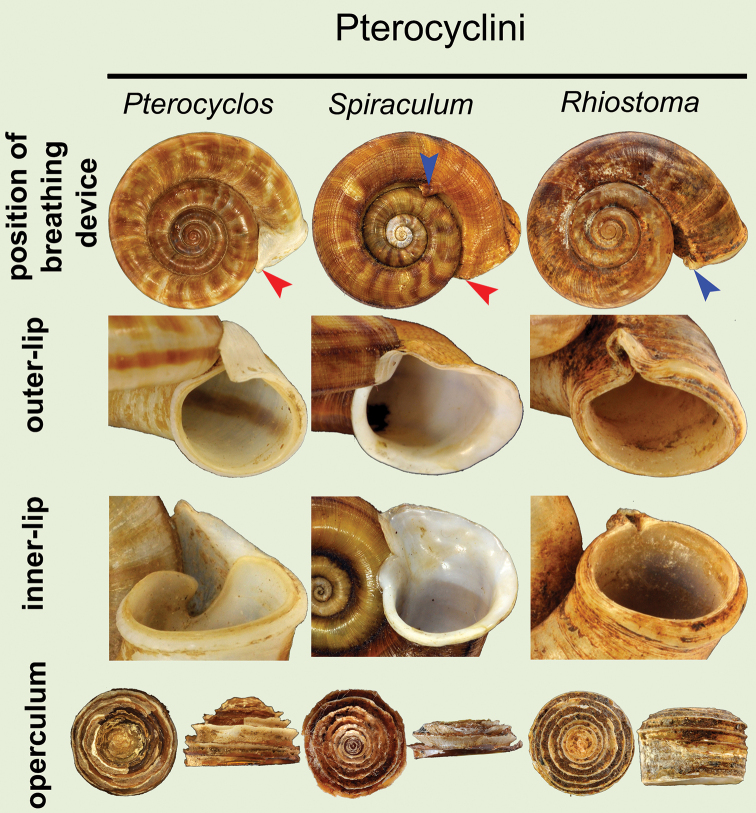
Comparison of breathing device and operculum characteristics among the five genera that closely resemble *Rhiostoma*. The specimens and opercula represented herein are the type specimen or topotype of the respective type species of each genus; ***Pterocyclos***: *P.rupestris* Benson, 1832 (lectotype UMZC I.102070, no locality data), ***Spiraculum***: *S.hispidum* Pearson, 1833 (specimen NHMUK 1906.4.4.72, from Khasi, Teria Ghat), ***Rhiostoma***: *R.haughtoni* Benson, 1860 (syntype UMZC I.103445, from Moulmein), ***Cyclotus***: *C.variegatus* Pearson, 1833 (specimen NHMUK 1896.6.7.34–35, from Palawan), ***Opisthoporus***: *Cyclostomataylorianum* Pfeiffer, 1851 = junior synonym of *Pterocyclosbiciliatum* Mousson, 1849 (specimen NHMUK 20210200 for shell, from Basan, Sarawak) and *Pterocyclostener* Menke, 1856 (NHMUK ex. Cuming collection for operculum, from Cochin China), and ***Ptychopoma***: *Cyclophoruschinensis* Möllendorff, 1875 (lectotype SMF 39415, from Lushan bei Kiukiang, China). Red arrows indicate the expansion of outer lip at upper junction of the peristome. Blue arrows indicate breathing tubes (apertural tube in *Rhiostoma* and sutural tubes in *Spiraculum* and *Opisthoporus*).

Shell small to large, and heliciform to depressed. Detached whorl absent or with short to long detached whorl, curved and descending. Breathing device prominently present with various types (Fig. 1B). Peristome double; lip thickened and expanded. Shell colour varying from uniform colour to zigzag pattern. Operculum calcareous, cup-shaped, and anticlockwise multispiral with elevated lamella (Figs 2B, 5).

###### Description.

***Shell*.** Shell flattened to heliciform, thick to thin, and widely umbilicate, showing all preceding whorls (Figs 1, 2). Detached whorl absent or present and short to long and/or curved and/or descending. Periostracum thick or thin corneous to brownish and transparent. Shell colour uniformly brownish to purplish and/or with brownish variegated streaks or zigzag patterns. Peristome continuous, double, multi-layered, or sometimes with inconspicuous boundary, and circular; lip thickened and/or slightly expanded. Breathing device located at upper junction of peristome and with form varying by species, i.e., knob, notch, canal, incomplete tube, complete tube, or tubular. Operculum calcareous, cup-shaped, thickened, concave, and inside, anticlockwise multispiral with elevated lamella, lateral fringe straight, and diameter considerably smaller than aperture width.

***Radula*.** Typical taenioglossate teeth arranged in inverted V-shaped row, each transverse row containing 7 teeth (2–1–1–1–2). Central tooth large, symmetrical, and triangular, and with 2–4 well-developed cusps on each side. Lateral and marginal teeth slightly slender, inclining toward central tooth, and with three or four cusps. Shape of teeth and number of cusps on each tooth vary by species.

**Figure 6.**
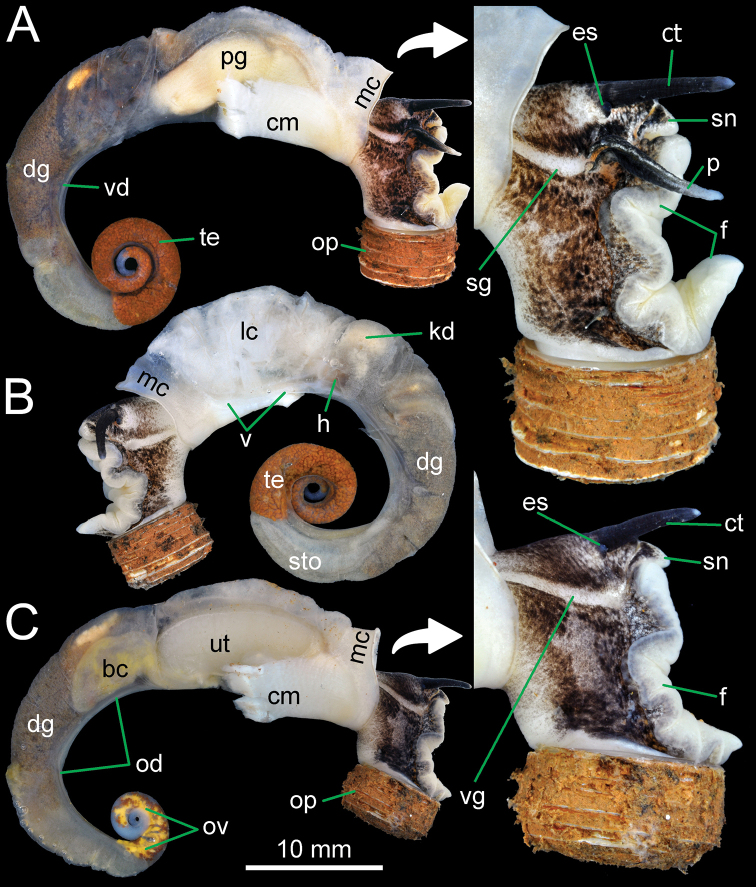
General anatomy of *Rhiostomahaughtoni* specimen CUMZ 10048/2 from Dhammatat Cave, Myanmar **A** right side of male testis and external penis and with enlargement of cephalic area **B** left side of male shows lung cavity and stomach **C** right side of female shows ovary and vaginal groove, and with enlargement of cephalic area. Abbreviations: **bc**, bursa copulatrix; **cm**, columellar muscle; **ct**, cephalic tentacle; **dg**, digestive gland; **es**, eyespot; **f**, foot; **h**, heart; **kd**, kidney; **lc**, lung cavity; **mc**, mantle collar; **od**, oviduct; **op**, operculum; **ov**, ovary; **p**, external penis; **pg**, prostate gland; **sg**, sperm groove; **sn**, snout; **te**, testis; **ut**, uterus; **vd**, vas deferens; **vg**, vaginal groove.

**Figure 7.**
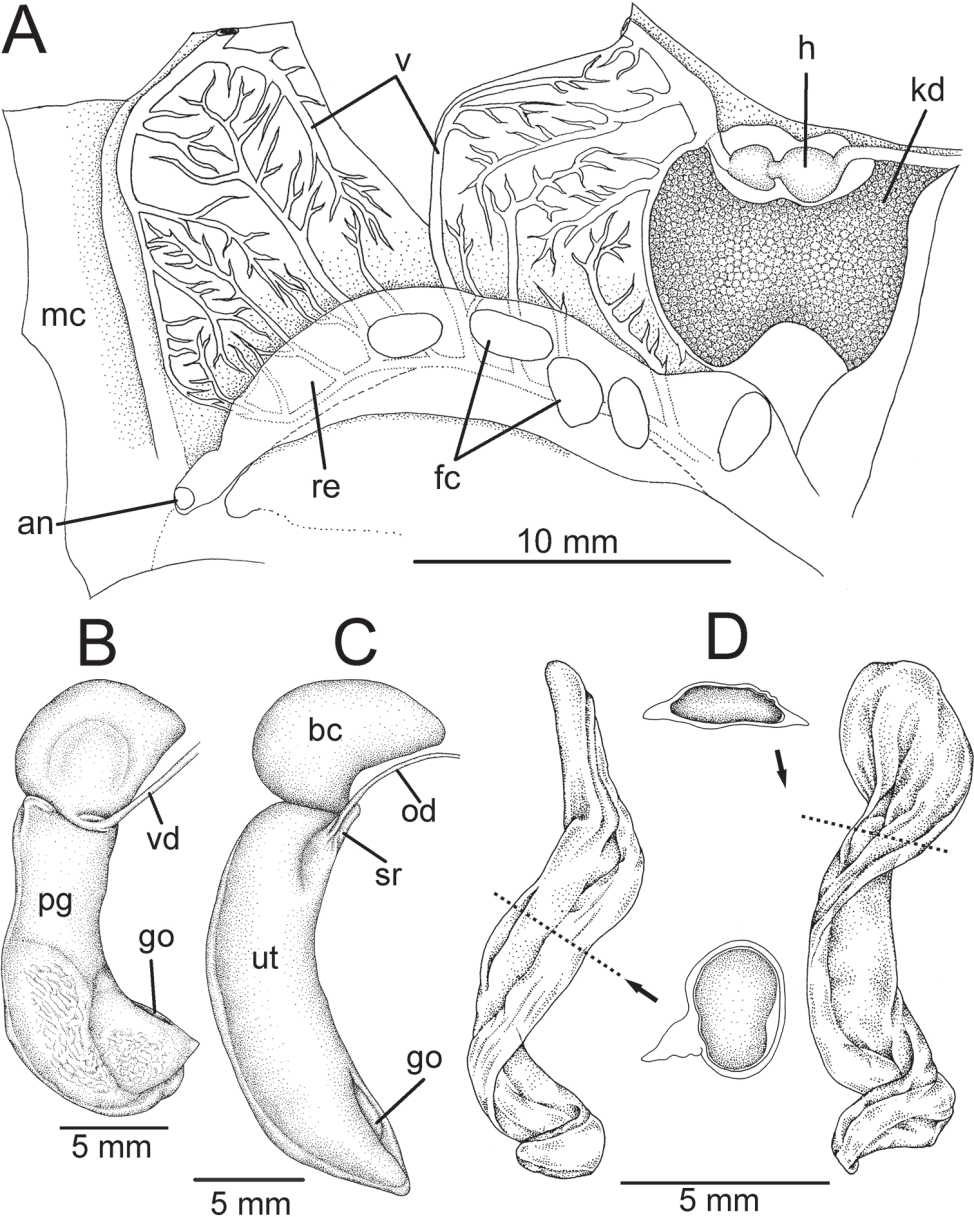
**A–C** Internal anatomy of *Rhiostomahousei* specimen CUMZ 3982 from Tham Sriwilai, Saraburi, **A** inside view of lung cavity shows an arrangement of the kidney, circulatory system and the terminal part of the digestive tract **B** male genitalia and **C** female genitalia **D** spermatophore of *Rhiostomasamuiense* specimen CUMZ 4713 from Kanchanadit, Surat Thani with its cross sections showing spermatophore shape in each position. Abbreviations: **an**, anus; **bc**, bursa copulatrix; **fc**, faeces; **go**, genital opening; **h**, heart; **kd**, kidney; **mc**, mantle collar; **od**, oviduct; **pg**, prostate gland; **re**, rectum; **sr**, seminal receptacle; **ut**, uterus; **v**, vein; **vd**, vas deferens.

***External features*.** Examination of many living snails indicates that the body colour varies from blackish to whitish, with or without brown to grey mottled spots spread over the body. This vast variation was present both within and between populations and depended on the age of snails. The internal reproductive organs and the external penis appeared very similar and provided no informative characters for distinguishing species. Therefore, the type species *R.haughtoni* (Figs 6, 7) and *R.housei* (Fig. 20) were selected as representative species for detailing the external and internal anatomical characters of the genus.

Animal exhibits dark brown to blackish patches and/or mottles scattered on blackish, brownish, or greyish body, faded near mantle cavity (Figs 6, 20). Head with a pair of long cephalic tentacles (ct), each one with a dark eyespot (es) at the outer base; snout (sn) broad and furrowed. The anterior body is short, and on the right side has a genital groove running downwards from the anterior end of the pallial cavity. Posterior body long; foot broad, with longitudinal pedal groove, and operculum (op) attached dorsally to posterior body. Animals are dioecious, the female possessing only a vaginal groove (vg) on the right side (Figs 6C, 20A); the male has both a long conical external penis (p) on the right side below cephalic tentacle and a sperm groove (sg) passing to tip of the external penis (Figs 6A, 20B).

***Internal features*.** Kidney (kd) brownish, triangular (Figs 6B, 7A). Heart (h) located on the left side of the kidney (on the right in the figure), pericardium thin, atrium slightly larger than ventricle. Lung cavity (lc) with large vein (v) and reticulated vessels. Rectum (re) large, bonded with genital apparatus (male prostate gland or female uterus), containing elliptical-shaped faeces (fc) and anteriorly tapering to the anus (an), which opens near mantle collar edge. Ctenidium and osphradium absent. Mantle collar (mc) smooth and slightly thickened. Columellar muscle (cm) broad and thick (Fig. 6A, C).

***Reproductive organs*.** Testis (te) with branched tubules, bright orange, and occupying ~ 2–3 whorls from the apex (Fig. 6A). Vas deferens (vd) a thin, straight tube attached to prostate gland at ~ 2/3 of its length proximal to genital opening. Prostate gland (pg) large, long, curved, pale yellowish in colour, and anterior end with a genital opening (go) located close to mantle collar (Fig. 7B). Sperm groove (sg) narrow, distinct, and connecting to genital opening on right side of snail with tip of penis. External penis (p) with long cylindrical shape but pointed tip and situated posteriorly below cephalic tentacles (Fig. 6A).

Ovary (ov) bright orange with multilobulated glands embedded within brownish digestive glands (Fig. 6C). Oviduct (od) a thin tube connecting ovary and uterus near base of seminal receptacle. Bursa copulatrix (bc) peanut-shaped, creamy to whitish in colour, and ~ 1/2 the length of uterus. Seminal receptacle (sr) small, pale orange, and located at the posterior end of the uterus. Uterus (ut) large, long, curved; posterior end rounded, and anterior end tapered, with a genital opening (Fig. 7C).

Spermatophores of two species, namely *R.samuiense* and *R.furfurosum* sp. nov., were examined. They appeared whitish, with a coiled cylindrical shape, and ~ 10 mm long (Fig. 7D). Anterior part slightly flat with compressed sperm sac, and bearing keels on both sides (see cross-section). The middle part has a swollen sperm sac (rounded in cross-section) and one prominent keel. Posterior part tapered, with a pointed end, and tiny sperm sac.

###### Distribution.

The genus *Rhiostoma* seems to have a limited distribution, occurring only in mainland Southeast Asia, including Cambodia, Laos, Myanmar, Thailand, Vietnam, peninsular Malaysia, and southern China (Benson 1860; Möllendorff 1894; Ancey 1898; Blanford 1902; Kobelt 1902, 1911–1914; Bavay and Dautzenberg 1909a, b; Gude 1921; Tomlin 1932, 1938; Salisbury 1949; Solem 1966). A few shells from Xishuangbanna, Yunnan Province, China, were collected and examined in this study. Within the overall limits of its total geographical distribution (Table 2), *Rhiostoma* occupies a more restricted area than other cyclophorids such as *Pterocyclos* and *Cyclotus*. The boundary of the genus is demarcated in the west with the endemic genera *Spiraculum* and *Theobaldius* Nevill, 1878 of South Asia; in the north with *Ptychopoma* and *Scabrina* Blandford, 1863 of China (Yen 1939); and in the south, with the dominant and closely related *Pterocyclos* in Greater Sunda Islands and the Philippines.

###### Remarks.

The reproductive anatomy of the Cyclophoroidea is little known and reports are scattered (i.e., Sarasin and Sarasin 1899; Weber 1925; Tielecke 1940; Jonges 1980; Kongim et al. 2013a; Sutcharit et al. 2014; Páll-Gergely et al. 2020b). A relatively comprehensive review of genitalic morphology of various cyclophorid genera was published by Tielecke (1940), and includes a brief note (without figures) of only one pterocyclinid species. Tumpeesuwan (2001) examined the reproductive anatomy of various *Rhiostoma* species and reported that it is relatively conserved, and that the only distinction observed was in the shape of bursa copulatrix in females. The length of the male genital opening of the *Rhiostoma* tended to be longer than the *Pterocyclos*. In addition, a well-developed seminal vesicle in male *Cyclophorus* seems to be the main character distinguishing it from the *Rhiostoma* and *Pterocyclos*, which do not have this character (see Weber 1925; Tumpeesuwan 2001). However, the taxonomic importance of the reproductive anatomical characters needs to be verified by further observations from various taxa. Nevertheless, the dissection of cyclophoroidian reproductive organs is difficult due to the indistinct organ boundary and watery or soft texture present in fresh specimens, whereas the ethanol-preserved specimens are far more fragile (S. Panha pers. obs.; Páll-Gergely et al. 2020b).

**Figure 8.**
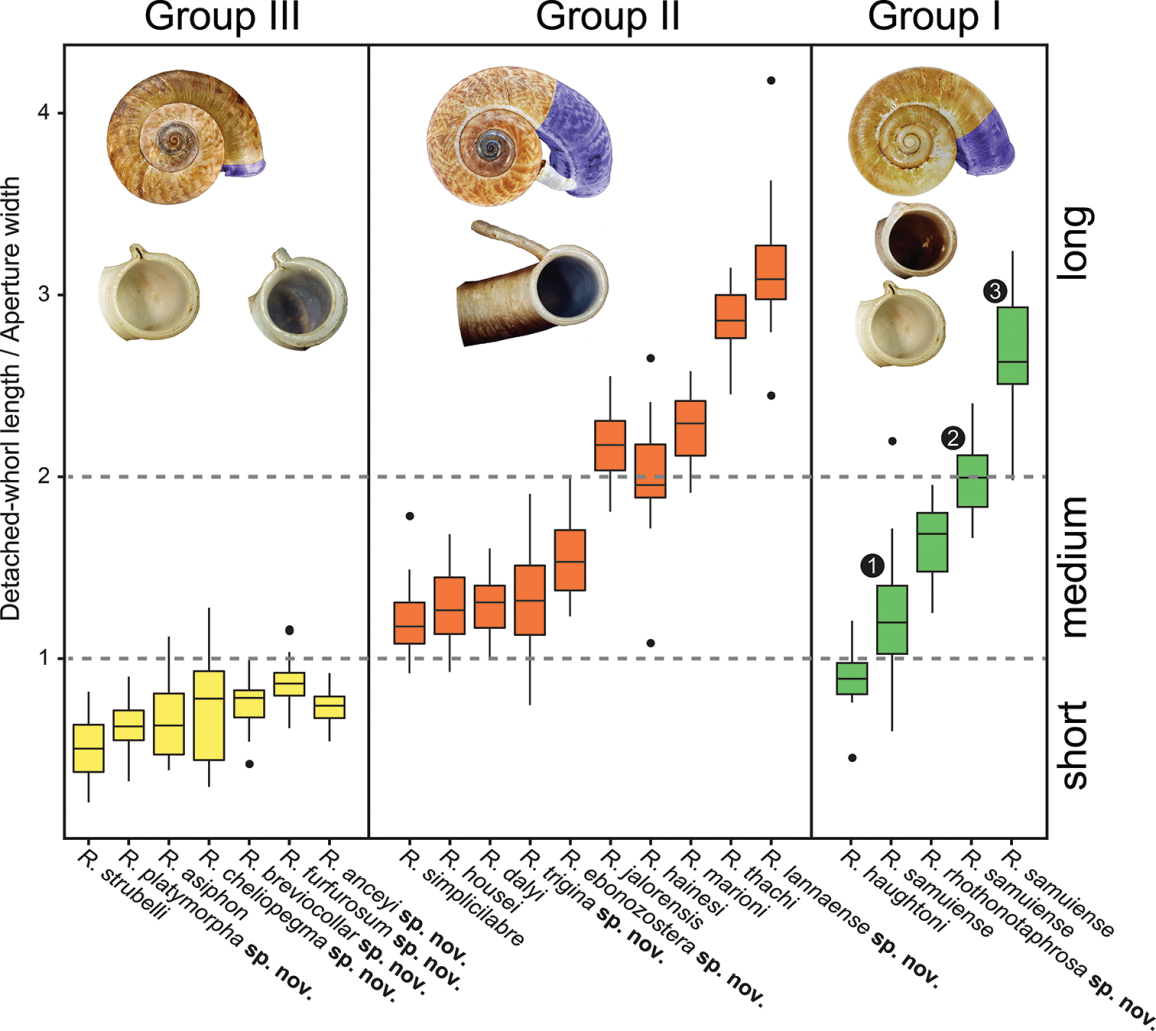
Box-and-whisker plots showing variation in the ratio of detached-whorl width to aperture width in some *Rhiostoma* species. Boxes show the median, 25^th^ and 75^th^ percentiles, whiskers show minimum and maximum observations, and circles show outliers. See text for the explanation of species grouping. 1 = topotype of *Rhiostomasamuiense* s. s. from Samui Island. 2 = topotype of “*Rhiostomachupingense*” from Bukit Chuping, Malaysia. 3 = specimens of “proboscis” morphotype from Surat Thani, Thailand.

The following species groupings are mainly based on the similarity in length of their detached whorl and in the shape of their breathing devices (Fig. 8). This informal subdivision is composed of four groups, and may assist in species identifications. No formal names or descriptions are provided, only the general characteristics of each group are provided.

**Group I**: *Rhiostomahaughtoni* group. This group can be recognised by a short to long detached whorl, and knob- or notch-shaped breathing device (Fig. 1B). This group is composed of three species: *R.haughtoni* Benson, 1860; *R.samuiense* Tomlin, 1932; and *R.rhothonotaphrosa* Tongkerd & Panha, sp. nov.

**Group II**: *Rhiostomahousei* group. Usually having a medium to long detached whorl, and breathing device as an incomplete tube or tubular (Fig. 1B). This group contains ten species: *R.housei* (Haines, 1855); *R.hainesi* Pfeiffer, 1862; *R.simplicilabre* Pfeiffer, 1862; *R.marioni* (Ancey, 1898); *R.dalyi* Blanford, 1902; *R.jalorensis* Sykes, 1903; *R.thachi* Huber in Thach, 2018; *R.ebenozostera* Tongkerd & Panha, sp. nov.; *R.lannaense* Tongkerd & Tumpeesuwan, sp. nov.; and *R.tigrina* Tongkerd & Tumpeesuwan, sp. nov.

**Group III**: *Rhiostomaasiphon* group. This group is characterised by a short detached whorl, and breathing device as an incomplete tube or notch (Fig. 1B). There are seven species belonging to this group: *R.asiphon* Möllendorff, 1893; *R.strubelli* Möllendorff, 1899; *R.abletti* Thach, 2016; *R.anceyi* Tongkerd & Inkhavilay, sp. nov.; *R.breviocollar* Tongkerd & Tumpeesuwan, sp. nov.; *R.furfurosum* Tongkerd & Panha, sp. nov.; and *R.platymorpha* Tongkerd & Tumpeesuwan, sp. nov.

**Group IV**: *Rhiostomamorleti* group. The heliciform or flattened shell is generally without a detached whorl (rarely very short) and with a notch- or canal-shaped breathing device. However, some species have a wide canal-shaped breathing device, in which the columellar side is completely attached to the preceding whorl, causing the whorl to appear attached. There are seven species belonging to this group: *R.cochinchinensis* (Pfeiffer, 1857); *R.cambodjense* (Morelet, 1875); *R.morleti* Dautzenberg & Fischer, 1906; *R.prestoni* Bavay & Dautzenberg, 1909; *R.cheliopegma* Tongkerd & Tumpeesuwan, sp. nov.; *R.gnomus* Tongkerd & Panha, sp. nov.; and *R.laosense* Tongkerd & Inkhavilay, sp. nov.

### ﻿Group I: *Rhiostomahaughtoni* group. Species with short to long detached whorl and knob-shaped or notch-shaped breathing device

#### ﻿1. Rhiostoma haughtoni

Taxon classificationAnimaliaArchitaenioglossaCyclophoridae

Benson, 1860

0B7E01ED-D09D-5DE0-B58D-40609E34EC14

**Figure 9.**
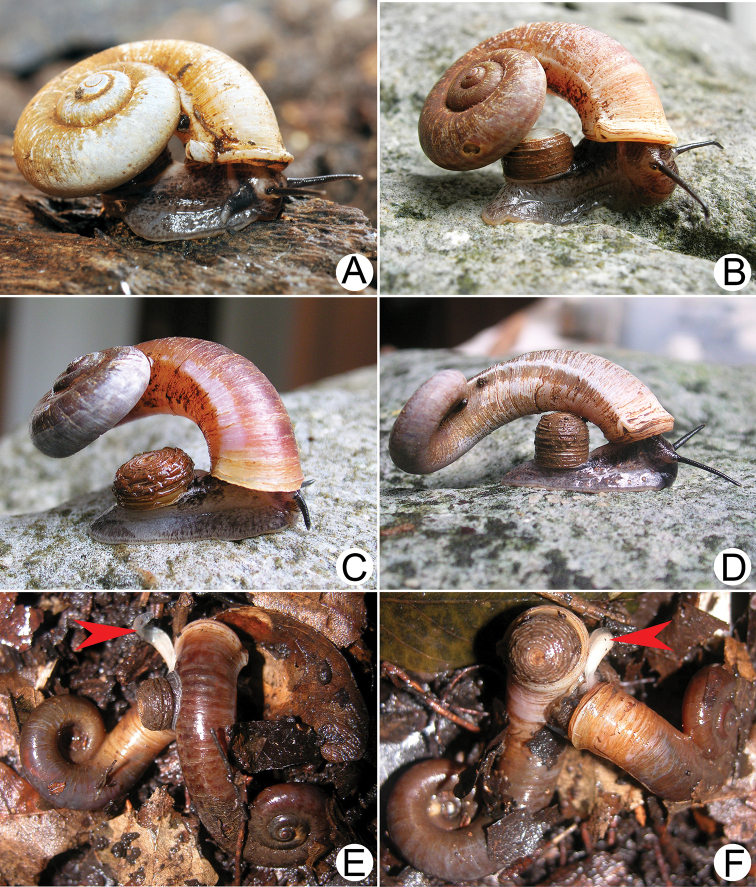
Live snails of the species group I **A***Rhiostomahaughtoni* specimen CUMZ 10048/2 from Dhammathat Cave, Myanmar **B–D***Rhiostomasamuiense***B** specimen CUMZ 3989 from Samui Island, Surat Thani **C** specimen CUMZ 3996 from Perlis, Malaysia (topotype of *Rhiostomachupingense*) **D** specimen CUMZ 4713 of “proboscis” morphotype from Surat Thani **E, F** copulation pair of *Rhiostomasamuiense* specimen CUMZ 4713 from Surat Thani shows unsuccessfully transferred spermatophore (female on the right in **E** and on the left in **F**), and the red arrows indicate a spermatophore illustrated in Fig. 7D. All figures are not to scale.

**Figure 10.**
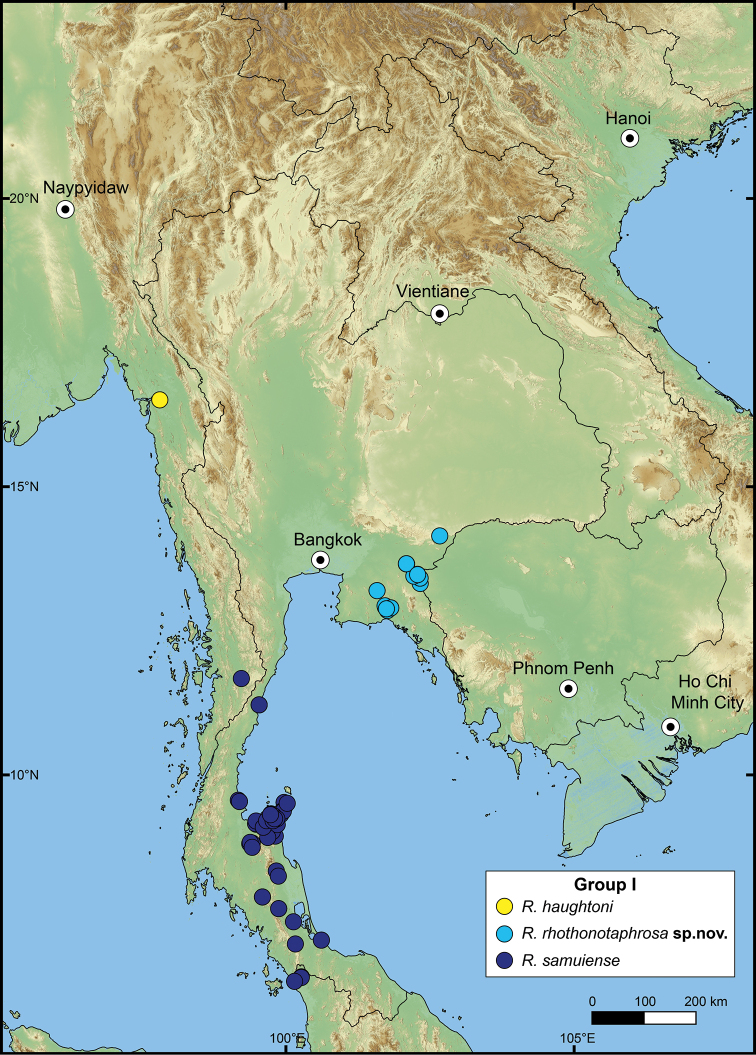
Distribution map of *Rhiostomahaughtoni*–species group.

**Figure 11.**
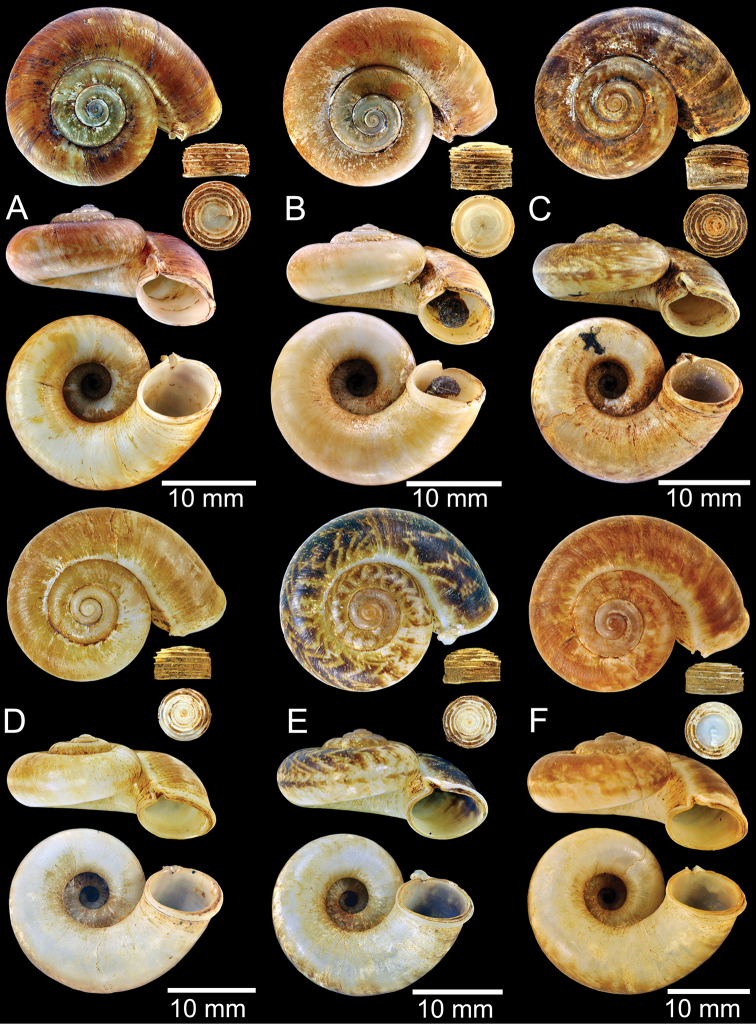
Shell of *Rhiostomahaughtoni***A** lectotype UMZC I.103445.A from Moulmein **B** paralectotype UMZC I.103445.B **C** specimen NHMUK 88.12.4.1985–1986 figured in Gude (1921, fig. 22) from Damaltha, Burma **D–F** specimens CUMZ 10048 from Dhammatat Cave, Mon, Myanmar.

Figs 9A , 10 , 11 , 16A

Rhiostoma haughtoni Benson, 1860: 96, 97. Type locality: ad cavernam Damatha, non procul ab urbe Moulmein [Dhammathat Cave, Mawlamyine, Mon State, Myanmar]. Pfeiffer 1865: 39, 40. Stoliczka 1871: 150. Kobelt and Möllendorff 1897: 115. Kobelt 1902: 177, 178, fig. 38. Kobelt 1911: 759, 760, pl. 110, fig. 14; pl. 111, figs 14–16. Gude 1921: 128, 129, fig. 22. Wenz 1938: 462, fig. 1166. Zilch 1956: 174. Rees 1964: 67, pl. 4, fig. 14. Preece et al. 2022: 77, 78, fig. 29e, f.
Pterocyclos (Rhiostoma) haughtoni —Hanley and Theobald 1870: 3, pl. 5, fig. 10.
Pterocyclus [sig] (Rhiostoma) haughtoni—Nevill 1878: 262.
Pterocyclos haughtoni —Reeve 1863: Pterocyclos pl. 5, species 30.

##### Type material.

The W.H. Benson collections are mainly held in UMZC, Cambridge, and most are considered the primary type specimens. However, Benson’s type specimens were not very explicit, and difficult to specify due to mislaid labels and subsequent substitution of the original labels (see Naggs 1997 and Preece et al. 2022 for more detail). However, in the original description of *R.haughtoni*, Benson stated that he had received several specimens, and one was alive. The W.H. Benson ex. R. MacAndrews collection contains one lot of two specimens. One specimen with a mummified soft body remaining inside the shell corresponds with Benson’s statement (Benson 1860: 96). This specimen lot was designated as the ***lectotype***UMZC I.103445.A (Fig. 11A) by Preece et al. (2022: 78), and another shell, UMZC I.103445.B (Fig. 11 B), becomes the paralectotype.

##### Other material examined.

**Myanmar**: Damaltha, Burma: NHMUK 88.12.4.1985–1986 (specimen with “x” was figured in Gude 1921, fig. 22; Fig. 11C), SMF 130508. Moulmein, Tenasserim, Burma: NHMUK 75.06.5.5 (4 shells), NHMUK 1903.7.1.1566 (2 shells), NHMUK ex. E.R. Sykes coll. Acc. 1825 (1 shell), NHMUK ex. Cuming coll. (1 shell), NHMUK ex. Oldham coll. (1 shell). Damatha, Burma: NHMUK ex. McAndrew coll. (4 shells). Dhammatat Cave, Mawlamyine District, Mon State: CUMZ 10048 (Figs 9A, 11D–F, 16A), CUMZ 10049.

##### Diagnosis.

Shell thick and depressed. Detached whorl short, curved and slightly descending. Breathing device notch-shaped. Shell colour uniformly brownish or with dark zigzag pattern.

##### Differential diagnosis.

In addition, the shell morphology of *R.haughtoni* differs from *R.housei* in having a notch-shaped breathing device, and a short detached whorl, while *R.housei* bears a tubular breathing device and the detached whorl is medium in length.

##### Description.

***Shell*.** Shell small to medium, cW 16.9–20.4 mm, cH 8.7–10.9 mm, thickened, and sub-discoidal to discoidal shape; detached-whorl length 3.0–8.0 mm. Apex acute; spire slightly elevated. Whorls 5 to 6, convex, increasing regularly; suture wide and deep; last whorl rounded. Shell surface with fine growth lines. Periostracum thick or thin, corneous, and transparent. Shell colour uniformly brownish, sometimes with dark brown zigzag pattern; ventral shell surface paler in colour; with or without a dark brown spiral band on periphery. Detached whorl shorter than apertural width, curved and descending. Peristome circular and double; lip thickened, slightly expanded and multi-layered. Aperture opened sub-laterally. Breathing device notch-shaped; outer lip protruding, with narrow groove; inner lip with shallow to deep incision. Umbilicus widely open and deep. Operculum calcareous, tall cup-shaped, and multispiral (Fig. 11).

***Radula*.** Taenioglossate radula arranged in inverted V-shaped rows. Central tooth with well-developed central cusp and two lateral cusps on each side; central cusp large with pointed tip; lateral cusps triangular and tapering in size. Lateral teeth consisting of four cusps; central cusp large, elongate, and flanked by pointed tip of two small inner cusps and one small outer cusp. Inner and outer marginal teeth each composed of three cusps; central cusp large and with dull tip, and flanked by pointed tip of one inner cusp and one outer cusp (Fig. 16A).

##### Distribution.

This species is currently known only from the type locality in Mon State, southern Myanmar (Fig. 10).

##### Remarks.

*Rhiostomahaughtoni* is often regarded as a junior synonym of *R.housei* from central Thailand (Habe 1965; Abbott 1989). However, *R.housei* exhibits significant intra-specific variation in shell morphology across its range. Furthermore, recent allozyme studies have suggested that *R.housei* is likely to represent a complex of cryptic species (Prasankok et al. 2011). In this study, the topotypic specimens of *R.haughtoni* clearly show a significant genetic distance from *R.housei*, proving it to be clearly distinct from *R.housei*.

The shell of *R.haughtoni* can vary from monochrome brownish or whitish without a peripheral band (Fig. 11D) to brownish zigzag patterns or blotches and thin peripheral band (Fig. 11E, F). The length of the detached whorl and shape of breathing device are relatively conserved, at least in the topotype population.

#### ﻿2. Rhiostoma samuiense

Taxon classificationAnimaliaArchitaenioglossaCyclophoridae

Tomlin, 1932

6176E53D-2C8F-53FC-8236-40C7A0A04095

**Figure 12.**
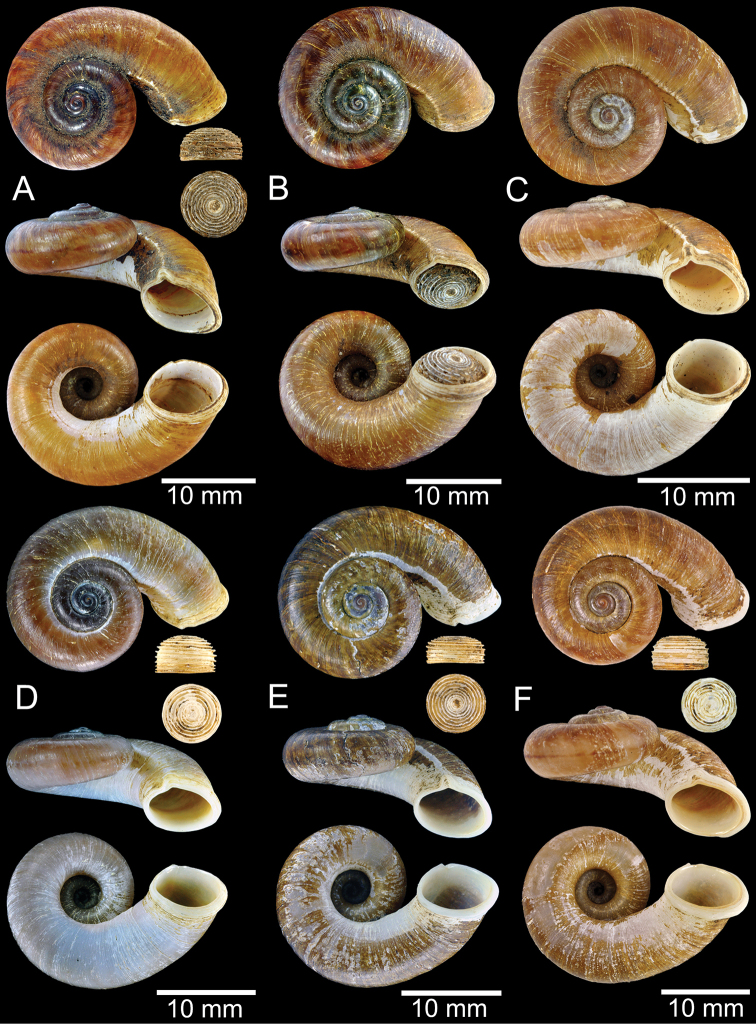
Shell of *Rhiostomasamuiense***A** syntype NMW 1955.158.1104 from Kaw Samui, Siam **B** syntype NMW 1955.158.1105 form type locality **C** specimen NMW 2.1981.118.20704 Kaw Samui **D** specimen CUMZ 3989 from Koh Samui, Surat Thani **E, F** specimen from Koh Tan, Surat Thani **E** specimen CUMZ 4791 and **F** specimen CUMZ 4707.

**Figure 13.**
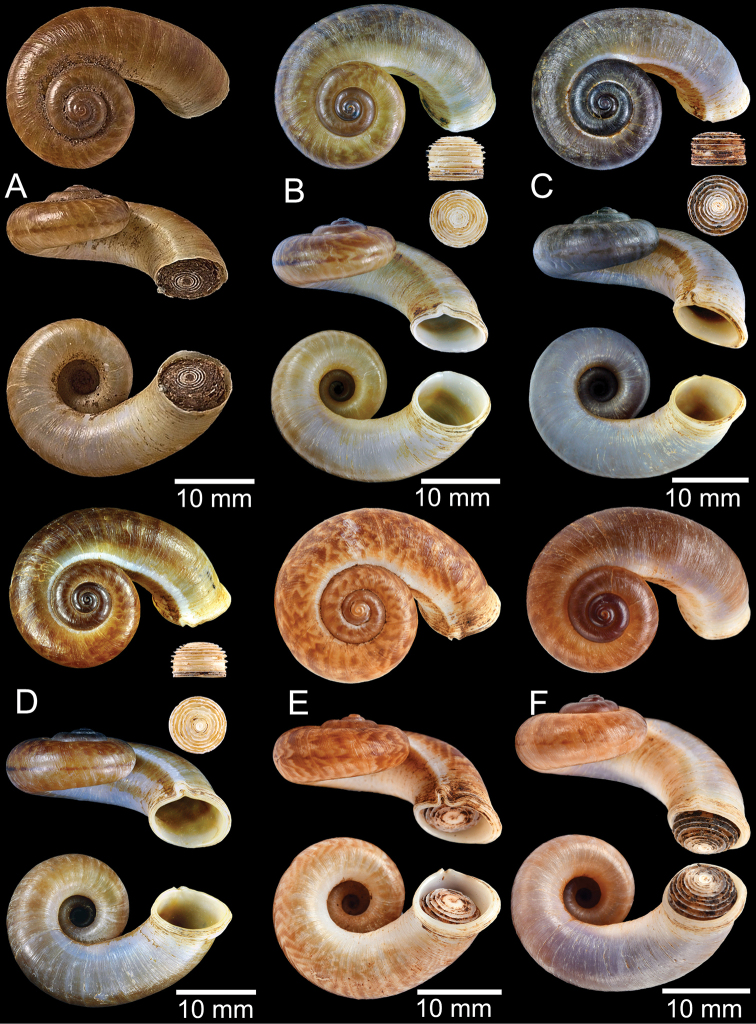
Shell of *Rhiostomasamuiense***A** lectotype of *R.chupingense*NHMUK 1938.10.25.1 from Bukit Chuping, Perlis, Malay State **B** specimen CUMZ 3996 from Bukit Chuping, Perlis, Malaysia (topotype) **C** specimen CUMZ 3986 from Bang Saphan, Prachuap Khiri Khan **D** specimen CUMZ 4708 from Khanom, Nakhon Si Thammarat **E** specimen CUMZ 3990 from Tha Chana, Surat Thani **F** specimen CUMZ 3997 from Sungai Jernih, Perlis, Malaysia.

**Figure 14.**
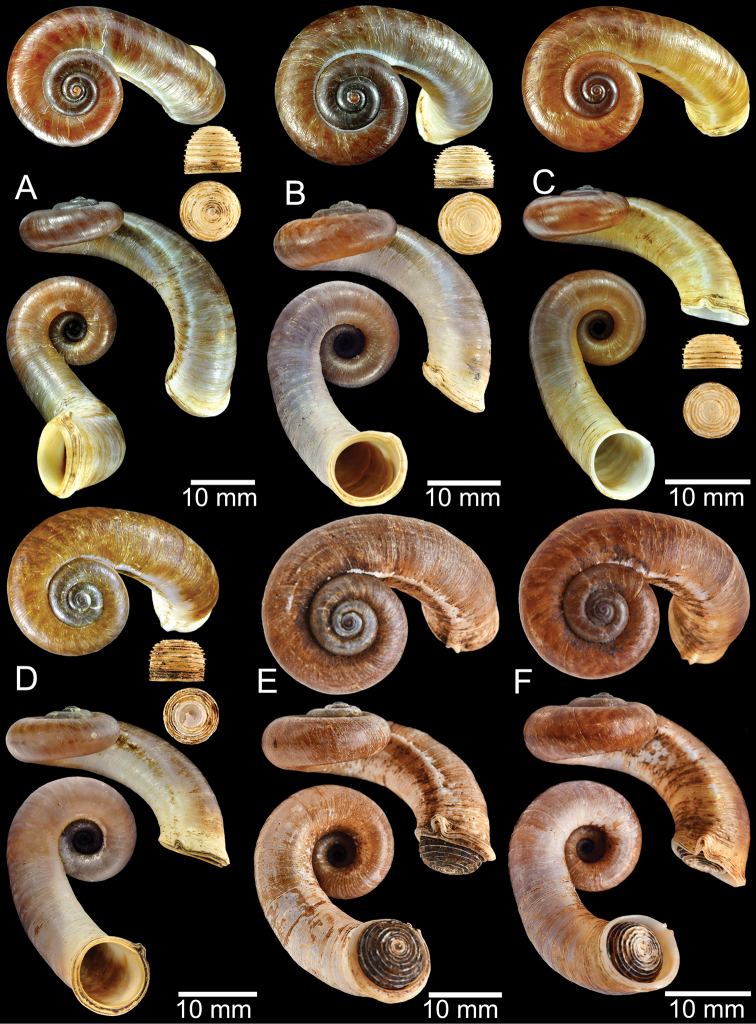
Shell of *Rhiostomasamuiense* of “proboscis” morphotype **A** specimen CUMZ 4712 from Kanchanadit, Surat Thani **B–D** specimens CUMZ 4713 from Kanchanadit, Surat Thani **E** specimen CUMZ 4790 from Kanchanadit, Surat Thani **F** specimen CUMZ 4786 from Sichon, Nakhon Si Thammarat.

Figs 9B–F , 10 , 12 , 13 , 14 , 16B, C

**Figure 15.**
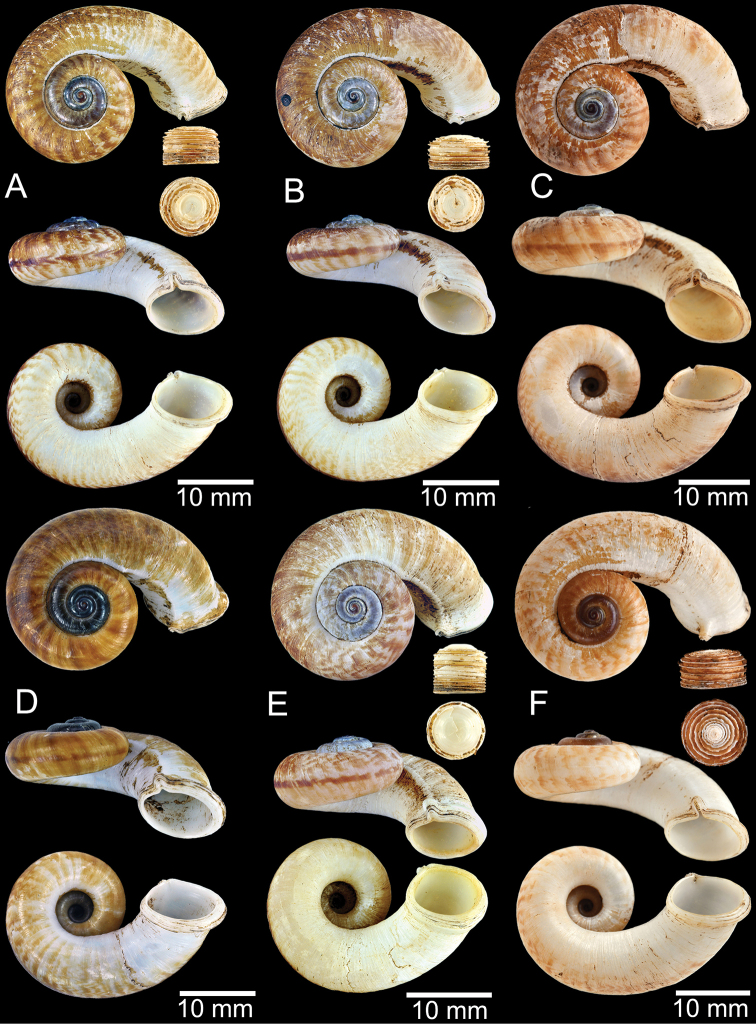
Shell of *Rhiostomarhothonotaphrosa* sp. nov. **A** holotype CUMZ 4709 from Klong Had, Srakeo **B, C** paratypes CUMZ 4710 from type locality **D, E** specimens CUMZ 4711 from Khao Chamao, Rayong **F** specimen CUMZ 4895 from Khao Chakan (north site), Srakeo.

**Figure 16.**
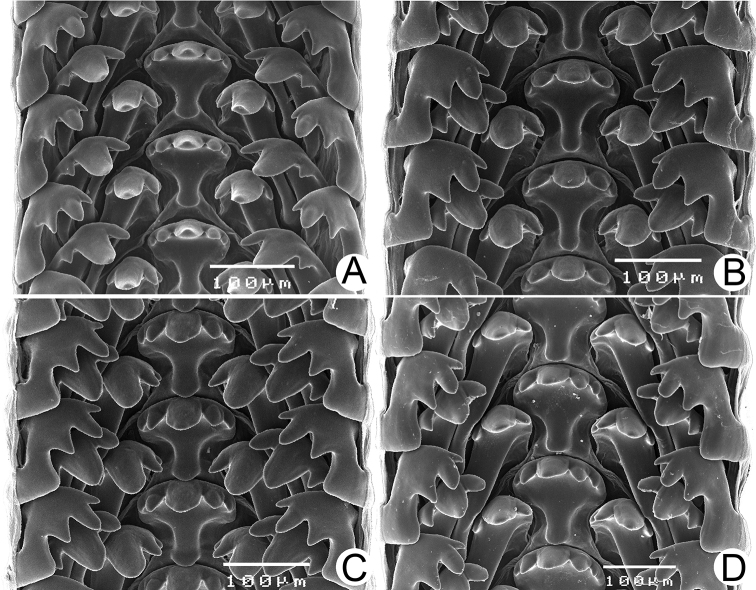
Radula morphology **A***Rhiostomahaughtoni* specimen CUMZ 10048/2 from Dhammatat Cave, Mon, Myanmar **B, C***Rhiostomasamuiense***B** specimen CUMZ 3989 from Samui Island, Surat Thani and **C** specimen CUMZ 3996 from Bukit Chuping, Perlis, Malaysia **D***Rhiostomarhothonotaphrosa* sp. nov. paratype CUMZ 4710 from Klong Had, Srakeo.

Rhiostoma samuiense Tomlin, 1932: 227, 228, pl. 26, with 2 figures. Type locality: Kaw Samui Island in the Gulf of Thailand [Samui Island, Surat Thani, Thailand]. Hemmen et al. 1999: 15, fig. 17. Patamakanthin 2001: 222, 223, fig. d1–d3. Tumpeesuwan 2001: 53–58, figs 4.16–4.18. Kongim et al. 2013b: 16, fig. 2i.
Rhiostoma chupingense Tomlin, 1938: 73, p1. 2, figs 1, 2. Type locality: Bukit Chuping, Perlis, Malaysia. Berry 1963: pl. 4, fig. 28. Hemmen et al. 1999: 26, figs 18.1–18.3. Patamakanthin 2001: 222, 223, figs b1, b2, c1–c3. Tumpeesuwan 2001: 29–34, figs 4.4–4.6. Tarruella and Doménech 2010: 189, fig. 1c. Kongim et al. 2013b: 16, fig. 2d. Sutcharit et al. 2019: 19, fig. 4c. New synonym
Rhiostoma “spec. 1”—Hemmen et al. 1999: 12, fig. 12.
Rhiostoma “sp. 2”—Hemmen et al. 1999: 13, 14, figs 13–16.
Rhiostoma sp.—Patamakanthin 2001: 222, 223, figs a1–a4.
Rhiostoma sp. 5—Tumpeesuwan 2001: 82–84, figs 4.31, 4.32.

##### Type material.

Tomlin (1932) had obtained several living specimens, and the original description included an illustration and one set of shell measurements. There are two lots from the Melvill-Tomlin collection, which are considered the ***syntypes***NMW 1955.158.01104 (1 shell; Fig. 12A) and NMW 1955.158.01105 (6 shells; Fig. 12B). ***Lectotype*** (designation in Sutcharit et al. (2019)) NHMUK 1938.10.25.1 (Fig. 13A) of *Rhiostomachupingense* Tomlin, 1938, from Bukit Chuping, Perlis, Malaysia; ***paralectotypes***NMW 1955.158.01101 (3 shells), NMW 2.1981.118.02703 (1 shell).

##### Other material examined.

**Malaysia**: Bukit Chuping, Kangar, Perlis: CUMZ 3844, 3996 (Figs 9C, 13B), 4746, 4768. Sungai Jernih Kangar, Kuala Perlis, Perlis: CUMZ 3850, 3997 (Fig. 13F), 4832. **Myanmar**: Lampane Cave, Ngawun, Tanintharyi: CUMZ 10214. **Thailand**: Kaw Samui: NMW 2.1981.118.20704 ex. T. Pain coll. (Fig. 12C). Khao Marong, Bang Sapan, Prachuap Khiri Khan: CUMZ 3865, 3874, 3986 (Fig. 13C), 4477, 4739, 4854, 4861, 4900, 10079, 10086, 10089. Ban Na Sarn, Surat Thani: CUMZ 4305, 4395. Khao Chongchang, Ban Na Sarn, Surat Thani: CUMZ 4306. Tham Kha Min, Ban Na Sarn, Surat Thani: CUMZ 4310. Wat Na Sarn, Ban Na Sarn, Surat Thani: CUMZ 3842. Donsak, Surat Thani: CUMZ 4308, 4309. Khao Chang, Donsak, Surat Thani: CUMZ 3866. Khao Pra, Donsak, Surat Thani: CUMZ 4777. Khao Sai, Donsak, Surat Thani: CUMZ 3992, 4759. Km8+500m, Donsak, Surat Thani: CUMZ 10094. Tham Khiriwong, Donsak, Surat Thani: CUMZ 3811, 10078. Wat Tham Po Ngam, Donsak, Surat Thani: CUMZ 3873. Kra Dae, Kanchanadit, Surat Thani: CUMZ 3869, 4790 (Fig. 14E). Tham Khuha, Kanchanadit, Surat Thani: CUMZ 4424, 4481. Wat Tham Petpanomwang, Kanchanadit, Surat Thani: CUMZ 3833, 3835, 3895, 3991, 4361, 4362, 4392, 4396, 4398, 4471, 4472, 4473, 4474, 4475, 4712 (Fig. 14A), 4713 (Figs 7C, 9D–F, 14B–D), 4721, 4741, 4782, 4785, 4793, 4802, 4812, 4875, 10065, 10066, 10067, 10069. Hin Lad Waterfall, Koh Samui, Surat Thani: CUMZ 3864, 4373, 4413, 4834, 4893. Koh Samui, Surat Thani: CUMZ 4307, 4332, 4333, 4334, 4335, 4397, 10073. Koh Tan, Koh Samui, Surat Thani: CUMZ 3863, 3890, 4382, 4415, 4432, 4707 (Fig. 12F), 4791 (Fig. 12E), 10070. Koh Wang Nok, Koh Samui, Surat Thani: CUMZ 3385, 3891. Na Muang Waterfall, Koh Samui, Surat Thani: CUMZ 4800, 3862, 3989 (Figs 9B, 12D, 16B), 4780. Wat Tham Yai, Tha Cha Na, Surat Thani: CUMZ 3990 (Fig. 13E), 4476, 4722, 4769, 4787, 4857, 4863, 4891, 4896, 10084, 10093, 10095. Wat Wichit Dittharam, Tha Cha Na, Surat Thani: CUMZ 10085. Wat Khao Hauy Hang, Hauy Yod, Trang: CUMZ 3840, 3846, 3945, 3993, 4774. Samet Chun Waterfall, Kanom, Nakhon Si Thammarat: CUMZ 4773. Tham Khao Krot, Kanom, Nakhon Si Thammarat: CUMZ 3944, 4000, 10087, 10092. Tham Wang Thong, Kanom, Nakhon Si Thammarat: CUMZ 3820, 4311, 4388, 4708 (Fig. 13D), 4781, 10091. Thongnian, Kanom, Nakhon Si Thammarat: CUMZ 10090. Wat Ao Sadet, Kanom, Nakhon Si Thammarat: CUMZ 10088. Tham Nam Wang Srithammasokkarach, Lan Saka, Nakhon Si Thammarat: CUMZ 4855. Wat Tham Khao Dang, Ron Phiboon, Nakhon Si Thammarat: CUMZ 10068. Wat Tham Khao Dang, Hintok, Ron Phiboon, Nakhon Si Thammarat: CUMZ 3838. Khao Chang (Sikeed), Sichon, Nakhon Si Thammarat: CUMZ 3894, 3999, 4786 (Fig. 14F). Khao Hu Thu, Khao Noi, Sichon, Nakhon Si Thammarat: CUMZ 3871. Khao Nam Lark (Sikeed), Sichon, Nakhon Si Thammarat: CUMZ 3893, 3898, 4757. Wat Tham Pra Chaison, Khao Chaison, Patthalung: CUMZ 10096. Khao Pu-Khao Ya, Si Banphot, Patthalung: CUMZ 3995, 4304, 4375, 4399, 4811, 10076, 10077. Khao Roop Chang, Padang Besa, Songkhla: CUMZ 3849, 4479, 10097. Tham Srikaesorn, Rattaphum, Songkhla: CUMZ 4488.

##### Diagnosis.

Shell thick and nearly flattened. Detached whorl medium to long in length, curved, descending, and twisted. Breathing device knob-shaped. Shell colour uniformly brown to purplish or sometimes with brownish zigzag pattern. Operculum calcareous, and tall cup-shaped.

##### Differential diagnosis.

*Riostomasamuiense* can be distinguished from *R.haughtoni* in having a brown to purplish shell, knob-shaped breathing device, and medium detached-whorl length. In comparison, *R.haughtoni* has a smaller shell, brown to whitish shell colour, a short detached whorl, and notch-shaped breathing device.

##### Description.

***Shell*.** Shell small, cW 15.1–18.9 mm, cH 8.1–10.7 mm, thick, and nearly flattened to sub-discoidal shape; detached-whorl length 3.5–14.5 mm. Apex acute with dark colouration. Whorls 4 to 5, convex, increasing regularly; suture wide and shallow; last whorl rounded and slender. Shell surface with fine growth lines. Periostracum thick, corneous, dark brown, and usually worn down on ventral surface. Shell colour uniformly brown to purplish (sometimes with brownish zigzag pattern); peripheral band usually absent (rarely present). Detached whorl medium to long, ~ 2× apertural width, curved, descending, and sometimes twisted. Peristome circular and double; lip thickened, expanded, and multi-layered. Aperture opened sub-laterally to ventrally. Breathing device knob-shaped; outer lip protruding, with small knob; inner lip with indistinct incision. Umbilicus widely opened and deep. Operculum calcareous, tall cup-shaped, and multispiral (Figs 12–14).

***Radula*.** Teeth arrangement and shape are very similar to *R.haughtoni*. Central tooth with large central cusp and two lateral cusps on each side. Lateral teeth composed of three to four cusps. Marginal teeth each composed of three cusps (Fig. 16B, C).

##### Distribution.

The previous records of this species range from the type locality in Samui Island, Gulf of Thailand, to the northern part of peninsular Malaysia, and the southern part of Thailand (Tomlin 1938; Patamakanthin 2001). The northern limit is around Bang Sapan, Prachuapkhirikhan, extending southward along the east coast of the Gulf of Thailand to northern peninsular Malaysia (Fig. 10).

##### Remarks.

The previous records of both *R.samuiense* and *R.asiphon* from “Samui Islands” were inaccurate (see Möllendorff 1893, 1894; Tomlin 1932). This study surveyed entire ranges of the Samui Islands area and found these two species to be allopatric. *Rhiostomaasiphon* has a distribution restricted to the Angthong Islands (probably the exact type locality), which are the limestone islands ~ 30 km northwest of Samui Island. In contrast, *R.samuiense* has a narrow range only on the granitic Samui Island (type locality) and its surrounding satellite islands.

Previously, *R.chupingense* Tomlin, 1938, had long been recognised as a distinct species mainly distributed in northern peninsular Malaysia and southern Thailand (Berry 1963; Sutcharit et al. 2019). The COI barcoding of the topotype of *R.chupingense* together with other populations from southern Thailand and the topotype of *R.samuiense* indicate these specimens cluster in a monophyletic clade and with low genetic distance (Figs 3, 4; Appendix 1: Table A1). Therefore, we consider *R.chupingense* Tomlin, 1938 as a junior subjective synonym of *R.samuiense*. This new assignment has made the distribution range of *R.samuiense* much broader on the Malay Peninsula.

Morphological variation occurs in *R.samuiense*, especially in the length of the detached whorl, which can be divided into three morphotypes:

1. The “typical” morphotype tends to have medium detached-whorl length, approximately the same as apertural width. The specimens tend to have thickened and brownish periostracum. Currently, this typical morphotype occurs only from the type locality.
2. The “ *chupingense*” morphotype is similar to the “typical” but has a long detached whorl (~ 2× longer than aperture width; detached-whorl length 11.0–18.0 mm, aperture width 6.1–8.1 mm) that is curved, descending and turned so that aperture opens sub-laterally. Some populations tend to have obvious brownish zigzag colour patterns. This morphotype has a wide distribution ranging from Perlis, Malaysia, to the southern part of Thailand and one locality in Myanmar.
3. The “proboscis” morphotype exhibits a markedly long detached whorl (~ 3× longer than aperture width; detached-whorl length 16.0–24.0 mm, aperture width 7.3–8.4 mm) that is curved, descending, and twisted, which turns the aperture to open ventrally. This morphotype is restricted to a few localities in the northern part of the Khao Luang Mountains.

#### ﻿3. Rhiostoma rhothonotaphrosa

Taxon classificationAnimaliaArchitaenioglossaCyclophoridae

Tongkerd & Panha sp. nov.

31284397-670B-522A-8FF1-97FBD4A20EEF

https://zoobank.org/547E244E-54FE-41D3-A990-B7B87BC30947

Figs 10 , 15 , 16D

Rhiostoma sp. 6 —Tumpeesuwan 2001: 85–89, figs 4.33–4.35.
Rhiostoma sp. 9 —Tumpeesuwan 2001: 96–99, figs 4.40, 4.41.

##### Type material.

***Holotype***CUMZ 4709 (cW 21.0 mm, cH 11.8 mm, dL 22.7 mm; Fig. 15A). ***Paratypes***CUMZ 3858 (25 adults + 12 juveniles), CUMZ 4710 (2 shells; Figs 15B, C, 16D), CUMZ 4823 (5 shells), NHMUK 20220438 (5 shells), and SMF 368673 (5 shells). All paratypes are from the type locality.

##### Type locality.

Tham Sri Thong, Khlong Had District, Srakeo Province, Thailand (13°28'43.6"N, 102°16'53.8"E). Limestone outcrop with dry deciduous forest, surrounded by sugarcane and tapioca farms.

##### Other material examined.

**Thailand**: Khao Wong Kot, Kaeng Hang Maeo, Chanthaburi: CUMZ 4852, 4874, 4877, 10174, 10176. Khao Cha Ang Oun, Bo Thong, Chonburi: CUMZ 4363, 4364. Khao Chamao-Khao Wong, Khao Chamao, Rayong: CUMZ 3808, 3857, 3923, 4337, 4368, 4711 (Fig. 15D, E), 10177, 10178. Khao Wong, Khao Chamao, Rayong: CUMZ 4725. Limestone near Khao Chamao Khao Wong, Khao Chamao, Rayong: CUMZ 3851. Khao Chakan, Khao Chakan, Srakeo: CUMZ 3806, 4369, 4370, 4735, 4820, 4829, 4882, 4895 (Fig. 15F), 4898, 10173, 10179. Tham Mahasombat, Khao Chakan, Srakeo: CUMZ 3830. Khao Siwa, Khlong Had, Srakeo: CUMZ 4371. Tham Leam, Khlong Had, Srakeo: CUMZ 3852. Tham Phet Po Thong, Khlong Had, Srakeo: CUMZ 4856, 4878, 10172. Wat Khao Pha Pheung, Khlong Had, Srakeo: CUMZ 10180. Ta Phraya, Srakeo: CUMZ 4862. Tham Khao Phlapphueng Thong, Wang Som Bun, Srakeo: CUMZ 10175.

##### Diagnosis.

Shell thick and flattened. Detached whorl long, curved, and descending. Breathing device notch-shaped. Shell colour whitish with brownish zigzag pattern. Operculum calcareous, tall and cup shaped.

##### Differential diagnosis.

This new species differs from *R.samuiense* from peninsular Thailand by having a notch-shaped breathing device and white shell with brownish zigzag colour pattern. In contrast, *R.samuiense* has a knob-shaped breathing device and generally monochrome brown to purplish shell. Although this new species has a notch-shaped breathing device similar to *R.haughtoni*, the distinct characters are longer detached whorl and dark apex, while *R.haughtoni* has a shorter detached whorl and without dark colour on apex. In addition, *R.rhothonotaphrosa* sp. nov. differs from *R.housei* and *R.simplicilabre* by having a notch-shaped breathing device and relatively longer and descending detached whorl. In contrast, these two species possess tubular breathing devices and a relatively shorter detached whorl.

##### Description.

***Shell*.** Shell medium, cW 19.8–24.9 mm, cH 9.7–12.5 mm, detached-whorl length 10.0–18.5 mm, thick, and sub-discoidal shape. Apex acute with dark colouration; spire slightly convex. Whorls 5 to 6, convex, increasing regularly; suture depressed and deep; last whorl rounded and slender. Shell surface with fine growth lines. Periostracum thick and brown. Shell with brownish zigzag pattern or sometimes with uniformly brownish colour; ventral surface with paler colour; with narrow dark brown spiral band on periphery. Detached whorl long, nearly 2× longer than apertural width, curved and descending. Peristome circular and double; lip thickened, slightly expanded, and multi-layered. Aperture opened sub-ventrally. Breathing device notch-shaped; outer lip protruding, with narrow groove; inner lip with shallow to deep incision. Umbilicus widely opened and deep. Operculum calcareous, tall cup-shaped, and multispiral (Fig. 15).

***Radula*.** Teeth arrangement and shape are almost identical to *R.haughtoni*. Central tooth with large central cusp and two lateral cusps on each side. Lateral teeth composed of three or four cusps. Marginal teeth each composed of three cusps (Fig. 16D).

##### Etymology.

The specific name *rhothonotaphrosa* comes from two Greek words, *rhothon* meaning nose and *taphros* meaning ditch or trench. It refers to the notch-shaped breathing device of this new species.

##### Distribution.

This new species is known from several localities in eastern Thailand near the Cambodia border in Srakeo, Chanthaburi, Rayong, and Chonburi provinces.

##### Remarks.

Four species, *R.hainesi*, *R.cambodjense*, *R.rhothonotaphrosa* sp. nov., and *R.cheliopegma* sp. nov., are distributed in eastern Thailand. Our survey throughout their distribution ranges revealed no sympatric populations except for a single locality with adjacent populations of *R.cambodjense* and *R.rhothonotaphrosa* sp. nov. These two species inhabit a small limestone outcrop of Khao Chakan, Srakeo Province; the outcrop is ~ 1 km long, 0.5 km wide, and oriented east-west. The new species occupies the northern side of the outcrop, while *R.cambodjense* commonly occurs on the southern flank of the same mountain. However, these two species are totally different in shell morphology, and their COI barcoding suggests they are not closely related taxa (Fig. 3).

### ﻿Group II: *Rhiostomahousei* group. Species with long detached whorl and tubular-shaped breathing device

#### ﻿4. Rhiostoma housei

Taxon classificationAnimaliaArchitaenioglossaCyclophoridae

(Haines, 1855)

03122B5F-ADC5-5EFE-8BC5-CF4838D12522

**Figure 17.**
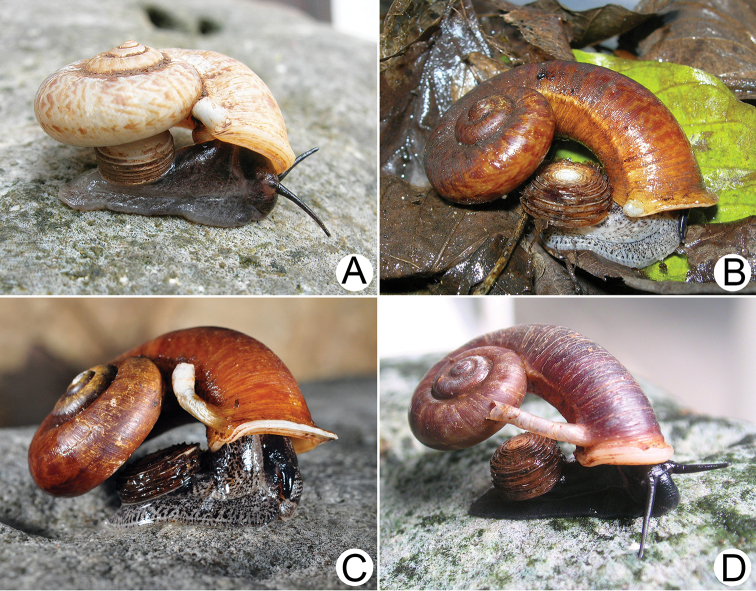
Live snails of the species group II **A***Rhiostomahousei* specimen CUMZ 3987 from Ao Manow, Prachuap Khiri Khan **B, C***Rhiostomahainesi* from Khao Soi Dao, Chanthaburi **B** without breathing device and **C** with long breathing device **D***Rhiostomajalorensis* specimen CUMZ 3994 from Wat Tham Sue, Krabi. All figures are not to scale. Photographs: P. Tongkerd (**B, C**).

**Figure 18.**
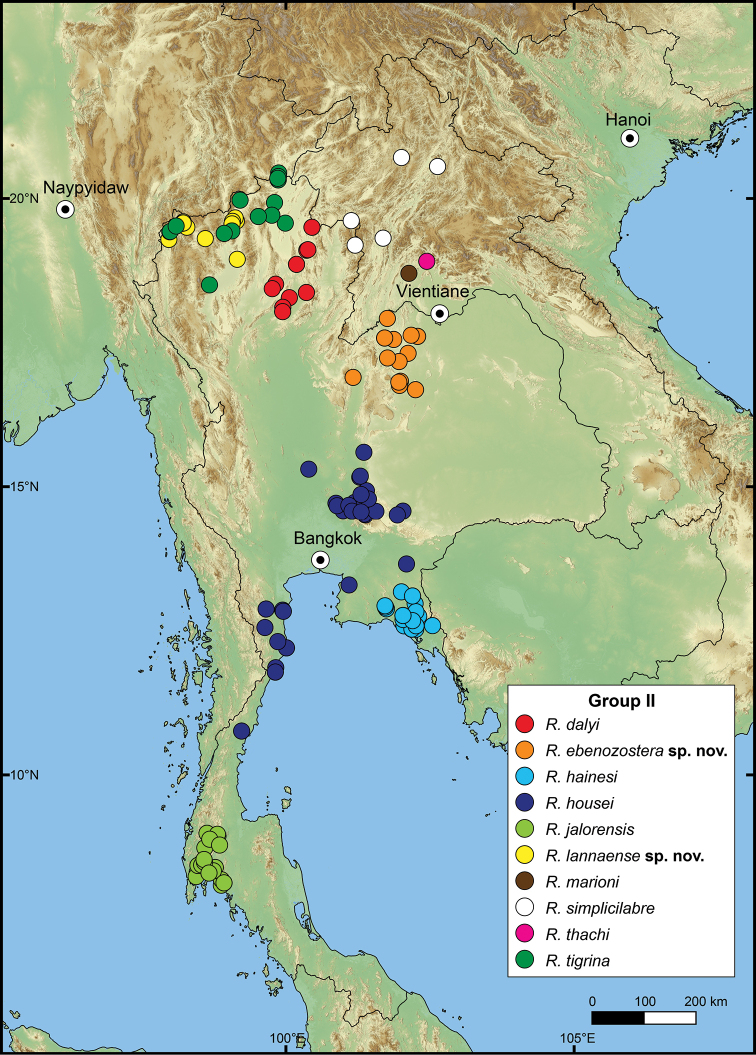
Distribution map of *Rhiostomahousei*–species group.

**Figure 19.**
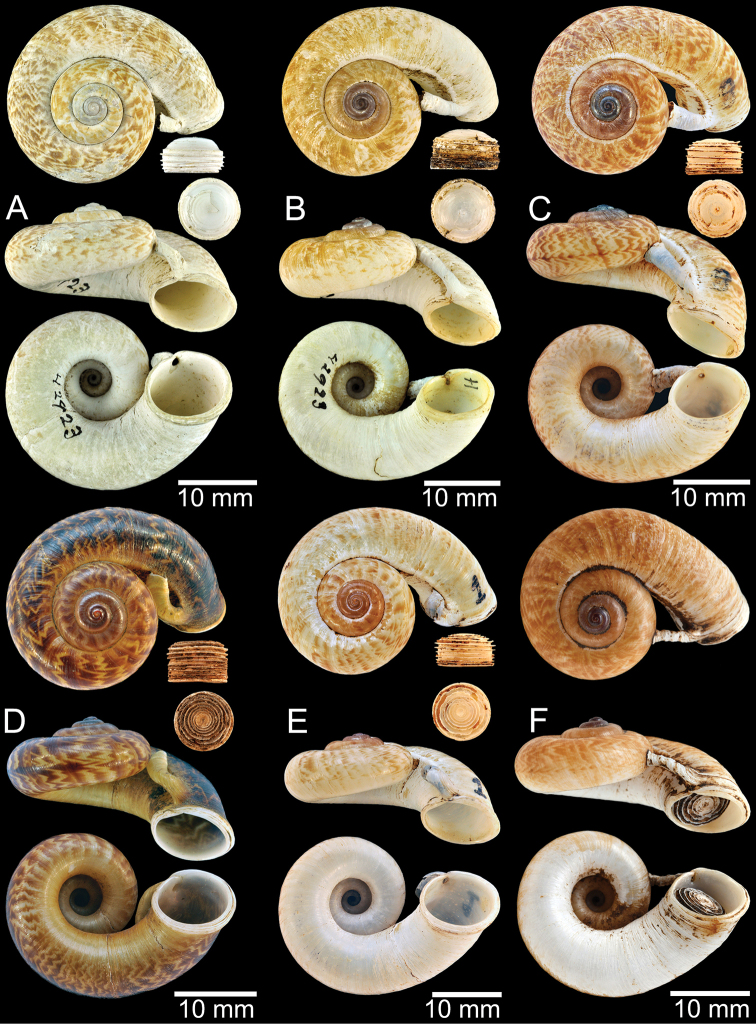
Shell of *Rhiostomahousei***A, B** syntype AMNH 42923 from Siam **A** figured specimen in Haines (1855: figs 12, 13) anb **B** other specimen from the same syntype lot **C** specimens CUMZ 4383 from Tham Phitsadan, Chumphon **D** specimen CUMZ 3982 from Tham Sriwilai, Saraburi **E** specimen CUMZ 4384 from Pak Chong, Nakhon Ratchasrima **F** specimen CUMZ 4879 from Hua Hin, Prachuap Khiri Khan.

**Figure 20.**
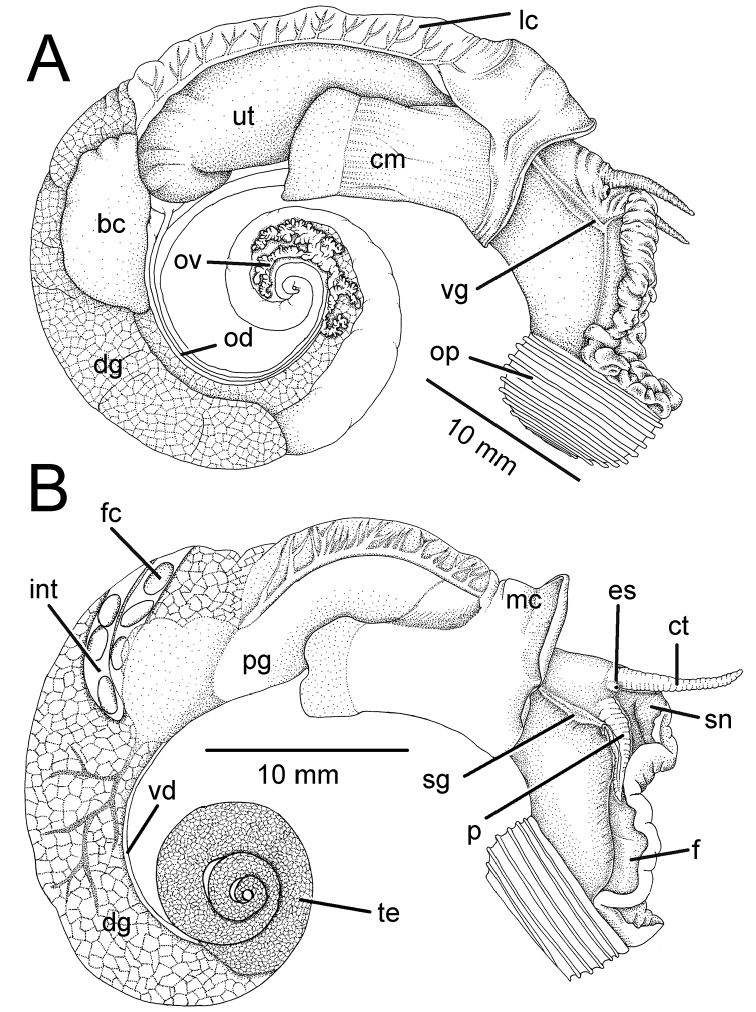
Drawing of general anatomy of *Rhiostomahousei* specimen CUMZ 3982 from Saraburi Province, Thailand. **A** female **B** male. Abbreviations: **bc**, bursa copulatrix; ; **cm**, columellar muscle; **ct**, cephalic tentacle; **dg**, digestive gland; **es**, eyespot; **f**, foot; **fc**, faeces; **int**, intestine; **lc**, lung cavity; **mc**, mantle collar; **od**, oviduct; **op**, operculum; **ov**, ovary; **p**, external penis; **pg**, prostate gland; **sg**, sperm groove; **sn**, snout; **te**, testis; **ut**, uterus; **vd**, vas deferens; **vg**, vaginal groove.

Figs 17A , 18 , 19 , 20 , 32A

Cyclostoma housei Haines, 1855: 157, pl. 5, figs 12–15. Type locality: Siam [= Thailand]. Boyko and Cordeiro 2001: 52.
Pterocyclos housei —Pfeiffer 1858: 29. Reeve 1863: Pterocyclos pl. 4, species 21.
Rhiostoma housei —Benson 1860: 97. Pfeiffer 1862a: 117, pl. 12, fig. 9. Pfeiffer 1865: 38. Martens 1867: 63. Fischer 1891: 101. Möllendorff 1894: 152. Kobelt and Möllendorff 1897: 115. Kobelt 1902: 178. Fischer and Dautzenberg 1904: 427. Kobelt 1911: 757, 758, pl. 110, figs 8–10, pl. 113, fig. 2. Zilch 1956: 174. Rees 1964: 67, pl. 4, fig. 15. Solem 1966: 11. Fischer 1973: 47. Abbott 1989: 29, 42, with 3 figures. Tumpeesuwan 2001: 41–46, figs 4.10–4.12. Kongim et al. 2013b: 16, fig. 2f, g.
Rhinostoma [sic] housei—Habe 1965: 128 (in part), pl. 2, fig. 10.
Pterocyclos (Spiraculum) housei —Martens 1860: 10.
Pterocyclus [sic] (Rhiostoma) housei—Nevill 1878: 263.
Cyclostoma (Pterocyclos) housei —Boyko and Cordeiro 2001: 52.

##### Type material.

This species was described from specimens from the Haines collection (AMNH). The unique name-bearing type was not designated; however, at least two specimens were illustrated in the original description. The specimen identical to the illustration (shape of breathing device) and measurements in Haines (1855: figs 12, 13) is figured herein, ***syntype***AMNH 42923 (5 shells; Fig. 19A, B) from Siam [Thailand].

##### Other material examined.

**Thailand**: Boong Tuey, East Siam: RBINS 659990, 659991; Pak Chong, East Siam: RBINS 659978, 659979, 659980; Lam Ton Lang, East Siam: RBINS 659982, 659983, 659984. Siam: NHMW 75000/E/23126 (1 shell). Samui, Siam: NHMW Rusnov (1 shell). Tham Phet Tham Thong, Takhli, Nakhonsawan: CUMZ 4385. Pukae Botanical Garden, Chaloem Phra Kiat, Saraburi: CUMZ 4742. Wat Tham Srivilai, Chaloem Phra Kiat, Saraburi: CUMZ 3982 (Figs 7A–C, 19D, 20, 32A), 4449. Kaeng Khoi, Saraburi: CUMZ 4315. Ban Tha Sao Village, Kaeng Khoi, Saraburi: CUMZ 10135. Wat Tham Phra Phothisat, Kaeng Khoi, Saraburi: CUMZ 10026, 10158. Chet Sao Noi Waterfall, Muaklek, Saraburi: CUMZ 4732. Muaklek, Saraburi: CUMZ 4390, 4460. Wat Tham Dao Khao Kaew, Muaklek, Saraburi: CUMZ 3802, 3934, 4389, 4760, 4842, 4897, 10131, 10133. Khao Sompod, Chai Ba Dan, Lopburi: CUMZ 3932, 3936, 3937. Wat Tham Promlok, Chai Ba Dan, Lopburi: CUMZ 10128. Sub Langka, Lamsonthi, Lopburi: CUMZ 4736. Khao Look Chang, Pak Chong, Nakhon Ratchasima: CUMZ 3805, 3981, 4316, 4318, 4320, 4379, 4384 (Fig. 19E), 4454. The way to Khao Yai, Pak Chong, Nakhon Ratchasima: CUMZ 4454. Wat Tham Prommachan Thammaram, Pak Chong, Nakhon Ratchasima: CUMZ 10160. Wat Tham Sub Muet, Pak Chong, Nakhon Ratchasima: CUMZ 10132. Wat Tham Thep Nimit, Pak Chong, Nakhon Ratchasima: CUMZ 10126. Wat Thepphitak Punnaram, Pak Chong, Nakhon Ratchasima: CUMZ 4321, 4416. Sakaerat Biosphere, Pak Thong Chai, Nakhon Ratchasima: CUMZ 4391. Sakaerat Environmental Research Station, Wang Nam Khiao, Nakhon Ratchasima: CUMZ 10134. Ban Bueng, Ban Bueng, Chonburi: CUMZ 4312. Khao Chakan, Srakeo: CUMZ 10156. Khao Nang Panturat, Cha Am, Phetchaburi: CUMZ 4879 (Fig. 19F), 10129, 10130, 10164, 10165. Khao Nang Panturat Nature Reserve, Cha Am, Phetchaburi: CUMZ 4480. Tham Na Kwang, Cha Am, Phetchaburi: CUMZ 3988, 4452, 4719, 10162. 30 km. before Pa La Au Waterfall, Hua Hin, Phetchaburi: CUMZ 3827, 4740. Kaeng Kachan, Phetchaburi: CUMZ 4409, 4412. Khao Lom Muak, Muang, Prachuap Khiri Khan: CUMZ 3870, 3987 (Fig. 17A), 4322, 10163. Bang Poo, Sam Roi Yod, Prachuap Khiri Khan: CUMZ 4414, 4417, 4429, 4430. Saun Wiweg Bureau of Monks, Sam Roi Yod, Prachuap Khiri Khan: CUMZ 10127. Ao Noi, Prachuap Khiri Khan: CUMZ 3868. Tham Pissadarn, Tha Sae, Chumphon: CUMZ 4383 (Fig. 19C), 4453, 4851.

##### Diagnosis.

Shell medium to large, thick, and depressed. Detached whorl longer than aperture width, curved, and descending. Breathing device straight with complete tube. Shell usually with dark brown zigzag patterns.

##### Differential diagnosis.

*Rhiostomahousei* can be distinguished from *R.tigrina* sp. nov. in usually having a stout detached whorl, generally a curved tubular breathing device, and a faded colour pattern. In contrast, *R.tigrina* sp. nov. has a slender detached whorl, generally a straight tubular breathing device, and a brownish zigzag colour pattern.

##### Description.

***Shell*.** Shell medium to large, cW 22.1–26.4 mm, cH 12.1–13.8 mm, thick, and sub-discoidal to discoidal shape; detached-whorl length 8.0–14.5 mm. Apex acute with dark colouration; spire slightly elevated. Whorls 5 to 6, convex and increasing regularly; suture wide and deep; last whorl rounded and stout. Shell surface with fine growth lines. Periostracum thin corneous, and transparent to thick with brownish colour. Shell colour whitish with dark brown zigzag pattern; dorsal shell surface with darker colour pattern than ventral surface; with narrow brownish spiral band on periphery (sometimes absent). Detached whorl of medium length, approximately the same as apertural width. Peristome circular and double; lip thickened, slightly expanded and multi-layered. Aperture opened sub-laterally. Breathing device tubular and its tip usually attached to preceding whorl; outer lip forms a long, curved or straight, closed tube; inner lip with small hole inside aperture. Umbilicus widely opened and deep. Operculum calcareous, tall cup-shaped, and multispiral (Fig. 19).

***Radula*.** Taenioglossate radula arranged in inverted V-shaped rows. Central tooth with well-developed central cusp and two lateral cusps on each side tapering in size; central cusp large with blunt tip; lateral cusps with triangular shape and pointed tip. Lateral teeth consisting of four cusps; central cusp large, elongate, and flanked by pointed tips of two small inner cusps and one outer cusp. Inner and outer marginal teeth each composed of three cusps; central cusp with large and dull tip, flanked by small pointed tips of one inner cusp and one outer cusp (Fig. 32A).

##### Distribution.

The type locality was given as a broad location of “Siam”; however, the specimens from central Thailand are the most similar to the syntype illustrated herein (Fig. 19A, B). In this study, *R.housei* has a wide distribution ranging from central to peninsular Thailand. The southernmost limits are around the Isthmus of Kra (Pratiew, Chumphon Province), and the northern limit is approximately in Nakhon Sawan Province (Fig. 18).

##### Remarks.

Previous literature recorded *R.housei* from several places in Laos, Cambodia, and Vietnam [Tonkin] (Möllendorff 1885; Ancey 1898; Fischer 1973). However, these records seem ambiguous because the type specimens have never been examined, were very limited in the number of specimens, and many species have a similar shell morphology to *R.housei*. Moreover, the species delimitation by previous authors is probably different from the concept of the type specimens. For example, this species has been reported in Chiangdao, Chiangmai, northern Thailand (sensu Solem 1966). We collected many specimens from several populations in the same area and examined Solem’s specimens in ZMUC. Due to the clear differences in shell characters, we recognise these populations as distinct species (*R.tigrina* sp. nov.). For that reason, the distribution range of *R.housei*, especially from Laos, Cambodia, and Vietnam, needs to be re-examined with new specimen collections with more precise locality.

Do et al. (2020b) reported several “*R.housei*” populations from Northern Vietnam in Dien Bien, Hao Binh, and Son La provinces. The figured specimen from Dien Bien Province shows the distance ~800 km away from the central population of this species from central Thailand. This specimen (Do et al. 2020b: fig. 6b, c) had a depressed spire, and a curved, tubular breathing device, and is probably *R.simplicilabre* rather than *R.housei*.

The specimens collected from southern Thailand (Chumporn, Phetchaburi, and Prachuapkhirikhan provinces) tended to have longer detached whorls, longer breathing devices, and brighter shell colour than the populations from central Thailand (typical). However, the shell colour pattern, detached whorl, and breathing device structures are identical to the type specimens (Fig. 19A, B). Therefore, these are attributed to morphological variation across the wide distribution range of this species.

#### ﻿5. Rhiostoma hainesi

Taxon classificationAnimaliaArchitaenioglossaCyclophoridae

Pfeiffer, 1862

8C1BB00D-BF89-5825-9604-0088370AD469

**Figure 21.**
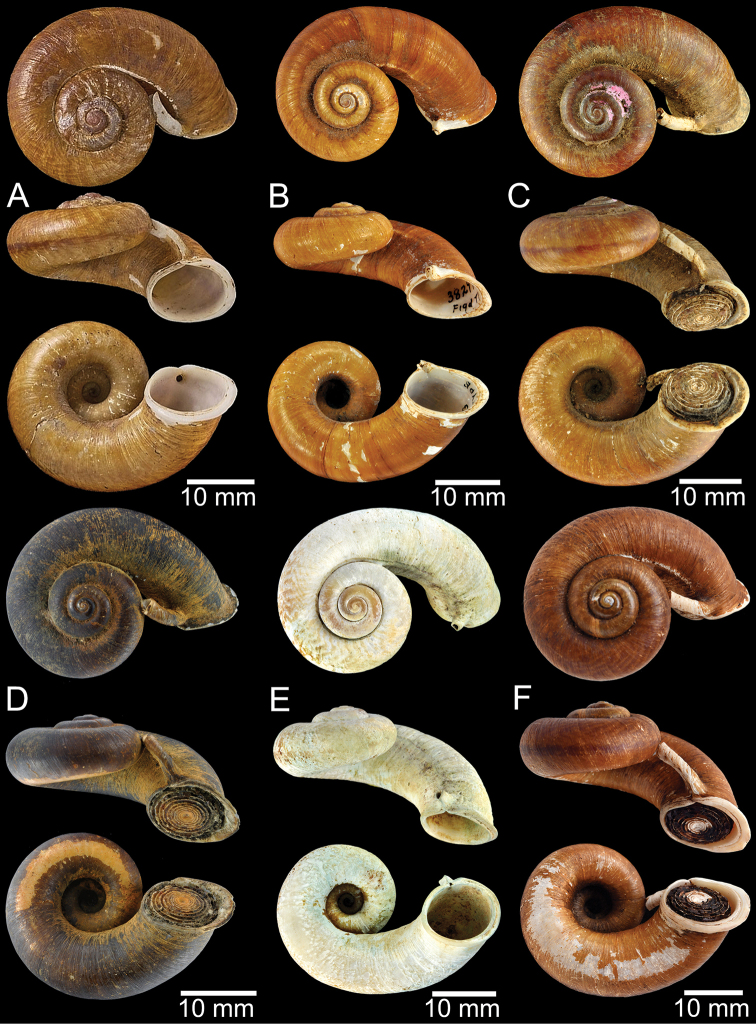
Shell of *Rhiostomahainesi***A** syntype NHMUK 20170371 from Camboja **B** holotype of “*Rhiosotmasmithi* Bartsch, 1932” NMNH 382943 from Khao Sabap, southern Siam **C, D** originally labelled as “*Rhiostomatomlini* Salisbury, 1949” from Khao Sabap, Siam **C** holotype NMW 1955.158.24924 and **D** paratype NHMUK 1949.6.7.1 **E** holotype of “*Rhiostomahouseikirai* Habe, 1965” NSMT 52242 from Chanthaburi, Thailand **F** specimen CUMZ 4381 from Chanthaburi.

**Figure 22.**
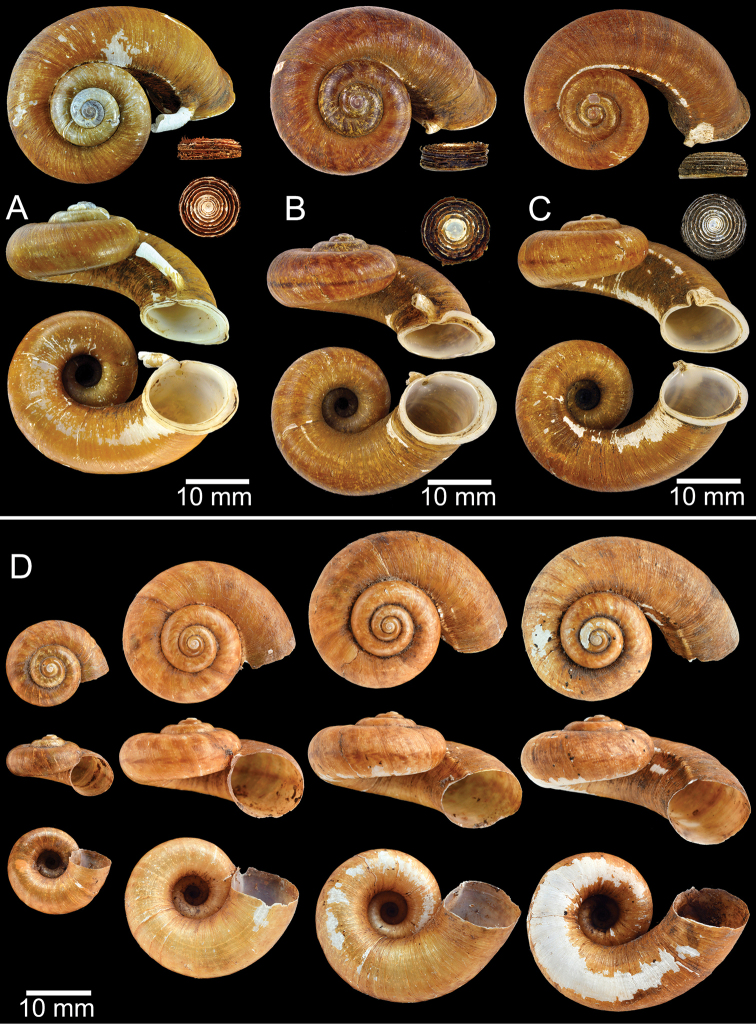
Shell of *Rhiostomahainesi***A** specimen CUMZ 4381 from Chanthaburi **B** specimen CUMZ 4814 from Makok Waterfall, Chanthaburi **C** specimen CUMZ 4816 from Khao Chamao, Rayong **D** specimens CUMZ 10194 from Chanthaburi shows series of growth from small juvenile (on the left) to nearly adult (on the right).

Figs 17B, C , 18 , 21 , 22 , 32B

Rhiostoma hainesi Pfeiffer, 1862a: 115, pl. 12, fig. 8. Type locality: Camboja [Cambodia]. Pfeiffer 1865: 38, 39. Martens 1867: 64. Fischer 1891: 101. Kobelt 1902: 178. Fischer and Dautzenberg 1904: 427. Kobelt 1911: 762, pl. 113, fig. 1. Fischer 1973: 47. Tarruella and Doménech 2010: 189, fig. 1d. Tumpeesuwan 2001: 35–40, figs 4.7–4.9. Kongim et al. 2013b: 16, fig. 2e. Sutcharit et al. 2019: 28, fig. 6f, g.
Pterocyclos hainesii [sic]—Reeve 1863: Pterocyclos pl. 4, species 19.
Pterocyclus [sic] (Rhiostoma) hainesi—Nevill 1878: 263.
Rhiostoma smithi Bartsch, 1932: 70, 71, fig. 1. Type locality: Khao Sabap, southern Siam [Khao Sabap, Khlung District, Chanthaburi Province, Thailand]. Abbott 1989: 34, with 2 figures (paratype). Solem 1966: 11. Panha and Thanamitramanee 1997: 2. Hemmen et al. 1999: 8, fig. 4. Patamakanthin 2001: 222, 223, fig. m1–m3. New synonym.
Rhiostoma tomlini Salisbury, 1949: 41, 42, pl. 3b, figs 3, 4. Type locality: Khao Sabap, Siam [Khao Sabap, Khlung District, Chanthaburi Province, Thailand]. Solem 1966: 11. Sutcharit et al. 2019: 51, fig. 12d. Chen et al. 2022: fig. 1b. New synonym.
Rhinostoma [sic] houseikirai Habe, 1965: 128 [in part], pl. 2, fig. 11. Type locality: Chanthaburi, Thailand. New synonym.

##### Type material.

***Syntype***NHMUK 20170371 (2 shells; Fig. 21A) from Camboja. ***Holotype***NMNH 382943 (Fig. 21B) of *Rhiostomasmithi* Bartsch, 1932, from Kao Sabap, S.E. Siam. ***Holotype***NMW 1955.158.24924 (Fig. 21C) of *Rhiostomatomlini* Salisbury, 1949, from Khao Sabap, Thailand, ***paratype***NHMUK 1949.6.7.1 (2 shells; Fig. 21D) from Khao Sabap, Siam, and ***paratype***NHMUK 20170372 (1 juvenile shell) from Khao Sabap, Siam. ***Holotype***NSMT 52242 (Fig. 21E) of *Rhiostomahouseikirai* Habe, 1965, from Chanthaburi, Thailand.

##### Other material examined.

**Thailand**: Khao Yai, Muang, Nakornnayok: CUMZ 4458. Khoa Sib Ha Chun, Kaeng Hang Maeo, Chanthaburi: CUMZ 10075, 10107. Klong Plub, Khlung, Chanthaburi: CUMZ 10017. Makok Waterfall, Khlung, Chanthaburi: CUMZ 3807, 3814, 3920, 3921, 4421, 4814 (Fig. 22B), 4819, 4827, 10102, 10105, 10109. Trok Nong Waterfall, Khlung, Chanthaburi: CUMZ 4455. Ban Ma Kham, Ma Kham, Chanthaburi: CUMZ 10099. Phrabat Khao Ma Kham, Ma Kham, Chanthaburi: CUMZ 10116. Khlong Narai waterfall, Muang, Chanthaburi: CUMZ 10101. Plieu Waterfall, Chanthaburi: CUMZ 3801, 4336, 4338, 4339, 4340, 4380, 4381 (Figs 21F, 22A), 4393, 4394, 4403, 4404, 4405, 4418, 4422, 4425, 4426, 4428, 4434, 10106, 10194 (Fig. 22D). Si Bha Chan Waterfall, Muang, Chanthaburi: CUMZ 4733. Hin Dat waterfall, Pong Nam Ron, Chanthaburi: CUMZ 10100. Khao Soi Dao Wildlife Breeding Center, Soi Dao, Chanthaburi: CUMZ 3855, 3883, 3898, 3917, 4402, 4423, 4457, 4743, 10114, 10117. Wat Khao Sukim, Tha Mai, Chanthaburi: CUMZ 3803, 3815, 3828, 3918, 3919, 4867, 10103, 10108, 10110, 10111, 10118, 10119, 10120. Wat Tham Klong Tip, Tha Mai, Chanthaburi: CUMZ 10112. Chanthaburi: CUMZ 10113. Khao Chamao-Khao Wong, Khao Chamao, Rayong: CUMZ 4456, 4816 (Fig. 22C), 4822, 10098. Khao Chamao Waterfall, Khao Chamao, Rayong: CUMZ 3897. Klong Pla Kang waterfall, Khao Chamao-Khao Wong, Khao Chamao, Rayong: CUMZ 10104. Wat Chat San Phatthanaram, Bo Rai, Trat: CUMZ 10115.

##### Diagnosis.

Shell large, thick, and depressed. Detached whorl longer than aperture width, curved and descending. Apertural lip much expanded on palatal wall. Breathing device usually tubular and its tip often attached to preceding whorl. Shell usually with thick dark brown periostracum.

##### Differential diagnosis.

*Rhiostomahainesi* differs from *R.rhothonotaphrosa* sp. nov. by having a large and stout last whorl, tubular- or notch-shaped breathing device, thickened brownish periostracum, and lip slightly thickened and widely expanded. In contrast, *R.rhothonotaphrosa* sp. nov. has a smaller shell size, slender last whorl, notch-shaped breathing device, white shell colour with brownish zigzag pattern, periostracum thin corneous, and lip thickened, slightly expanded, and multi-layered.

##### Description.

***Shell*.** Shell large, cW 22.1–27.8 mm, cH 11.3–15.2 mm, thick, and discoidal shape; detached-whorl length 11.0–28.5 mm. Apex acute; spire elevated. Whorls 5 to 6, convex, increasing regularly; suture wide and slightly deep; last whorl rounded and stout. Shell surface with fine growth lines. Periostracum thick corneous with brownish to dark brown colour (without periostracum shell uniformly whitish to yellowish colour); with narrow dark spiral band on periphery. Detached whorl long, ~2× longer than apertural width, curved and descending. Peristome circular and double; lip thickened, broadly expanded on palatal wall, and multi-layered. Aperture opened sub-laterally to sub-ventrally. Breathing device typically tubular (incomplete tube and notch shape sometime present), and its tip sometimes attached to preceding whorl; outer lip forms a short to long closed tube and is located just behind apertural lip; inner lip with small hole inside aperture (or deep incision in notch shape). Umbilicus widely opened and deep. Operculum calcareous, cup-shaped, and multispiral (Figs 21, 22).

***Radula*.** Teeth arrangement and shape are almost identical to *R.housei*. Central tooth with large central cusp and two lateral cusps on each side. Lateral teeth composed of four cusps; central cusp large with blunt tip; two inner cusps with pointed tips; one outer cusp small and dull. Marginal teeth each consisting of three pointed cusps (Fig. 32B).

##### Distribution.

The previous records of *R.hainesi* were mostly from vague locations in Cambodia and Thailand: Khao Sabap (= Plieu National Park, Chanthaburi) (Pfeiffer 1862a; Habe 1965). The recent study obtains specimens from several localities in Nakhonnayok, Rayong, and Chanthaburi provinces (Fig. 18).

##### Remarks.

Three nominal taxa have long been confused with *R.hainesi: R.smithi* Bartsch, 1932, *R.tomlini* Salisbury, 1949, and *R.houseikirai* Habe, 1965. The first two species were described based only on differences in shell colour and shapes of the breathing device. The type localities of both are from the same location at “Khao Sabap” [= Plieu National Park, Khlung, Chanthaburi, Thailand], and their holotypes are identical with *R.hainesi* (Fig. 21A–D). The last taxon, *R.houseikirai*, was nominated as a subspecies of *R.housei* (sensu Habe 1965). However, the holotype (Fig. 21E) exhibits a long and curved detached whorl, short tubular breathing device, expanded and thick apertural lip, and brownish periostracum (remaining inside umbilical area), which are the unique characters of *R.hainesi*. In addition, the type locality was Chanthaburi Province, which is within the distribution range of *R.hainesi*. Therefore, we recognise these three nominal taxa as junior subjective synonyms of *R.hainesi*.

The three variations in breathing device shape were reported in Tumpeesuwan (2001: fig. 5.2d–f): tubeless, incomplete tube, and complete tube. In this study, *R.hainesi* from Chanthaburi populations also exhibit sympatrically occurring morphs. The typical and more abundant morph has a short to long tubular breathing device (Figs 21F, 22A), and the less abundant morph has a notch-shaped breathing device (Fig. 22C). Specimens with these three distinct types of breathing devices were sampled for the COI analysis, and the results suggest that these are conspecific (Fig. 3).

#### ﻿6. Rhiostoma simplicilabre

Taxon classificationAnimaliaArchitaenioglossaCyclophoridae

Pfeiffer, 1862

5DDC17FD-CC49-5429-AB6E-A0F794A8A960

Figs 18 , 23

Rhiostoma simplicilabre Pfeiffer, 1862a: 115, pl. 12, fig. 7. Type locality: Camboja [Cambodia]. Pfeiffer 1865: 39. Fischer 1891: 101. Kobelt and Möllendorff 1897: 115. Kobelt 1902: 178, 179. Fischer and Dautzenberg 1904: 427. Kobelt 1911: 756, 757, pl. 110, figs 5–7, pl. 113, fig. 3. Sutcharit et al. 2019: 48, 49, fig. 11f, g.
Pterocyclos simplicilabris —Reeve 1863: Pterocyclos pl. 4, species 20.
Pterocyclus [sic] (Rhiostoma) simplicilabris—Nevill 1878: 262.

##### Type material.

**Figure 23.**
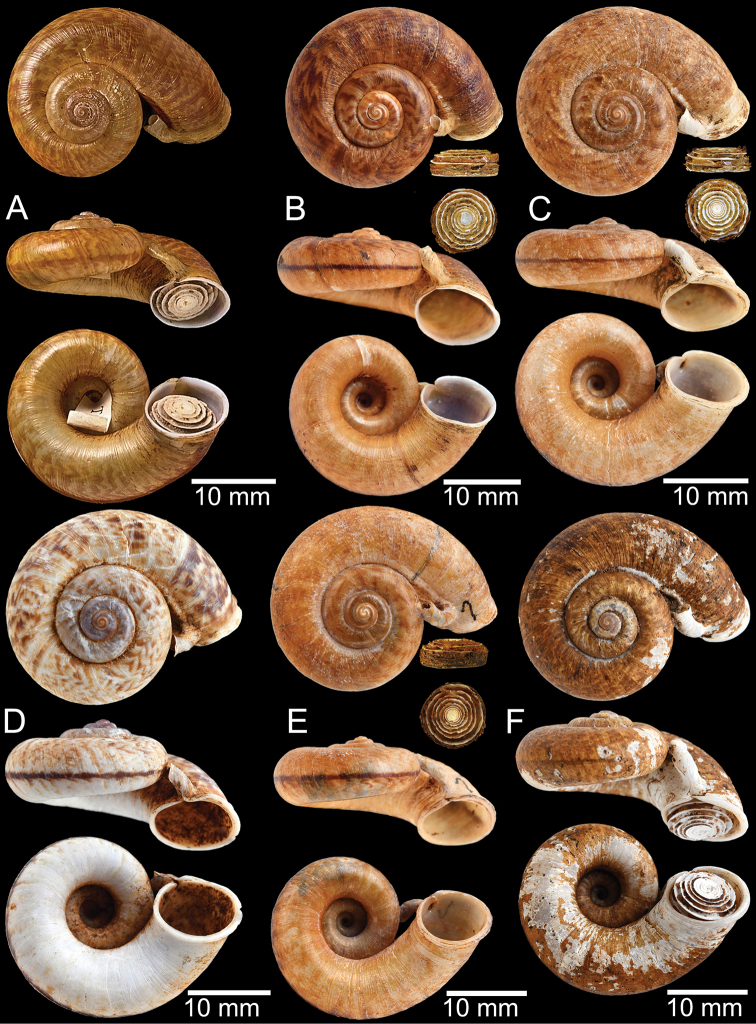
Shell of *Rhiostomasimplicilabre***A** syntype NHMUK 20130214 from Camboja **B, C** specimens CUMZ 10009/2 from Ngeun, Sayaboury, Laos **D** specimen CUMZ 10006 from Meuang Xai, Oudomxay, Laos **E** specimen CUMZ 10010/2 from Nong Khiaw, Luang Prabang, Laos **F** specimen CUMZ 4868 Bor Kluea, Nan.

***Syntype***NHMUK 20130214 (4 shells; Fig. 23A) from Camboja [Cambodia]

##### Other material examined.

**Laos**: Tham Pha Tok, Nong Khiaw, Luang Phrabang: CUMZ 10010/1, 10010/2 (Fig. 23E). Ban Oudom, Meuang Xai, Oudomxay: CUMZ 10006 (Fig. 23D). Ban Homsai, Meuang Ngeun, Sayaboury: CUMZ 10007, 10008. Ban Na Mone, Sayaboury: CUMZ 10009/1, 10009/2 (Fig. 23B, C). **Thailand**: Sa-pan Waterfall, Bo Kluea, Nan: CUMZ 4868 (Fig. 23F).

##### Diagnosis.

Shell medium, thin, and depressed. Detached whorl longer than aperture width and descending. Breathing device tubular, and often attached to preceding whorl. Shell with brownish zigzag patterns; thick corneous periostracum.

##### Differential diagnosis.

Based on the type and museum specimens, *R.simplicilabre* is very closely allied to *R.housei*. The differences are the tubular-shaped breathing device perpendicular to the detached whorl, depressed spire, and thickened periostracum. It differs from *R.hainesi* by having a brownish zigzag colour pattern, shorter detached whorl, and smaller shell size. In comparison, *R.hainesi* has a thick brownish to dark brown periostracum, longer detached whorl, and larger shell size.

##### Description.

***Shell*.** Shell medium, cW 20.9–26.1 mm, cH 9.7–12.8 mm, thin, and sub-discoidal to discoidal shape; detached-whorl length 7.5–12.5 mm. Apex acute; spire slightly elevated. Whorls 5 to 6, convex, increasing regularly; suture wide and deep; last whorl rounded and stout. Shell surface with fine and regular growth lines. Periostracum thick corneous, and transparent. Shell with brownish zigzag patterns; ventral shell surface with paler colour and less brownish pattern; with narrow dark brown spiral band on periphery. Detached whorl long and almost same length as apertural width, curved and descending. Peristome circular and double; lip somewhat thin and slightly expanded. Aperture opened sub-laterally. Breathing device tubular and its tip usually attached to preceding whorl; outer lip forming short, curved, closed tube located just behind apertural lip; inner lip with small hole inside aperture. Umbilicus widely opened and deep. Operculum calcareous, cup-shaped, and multispiral (Fig. 23).

**Figure 24.**
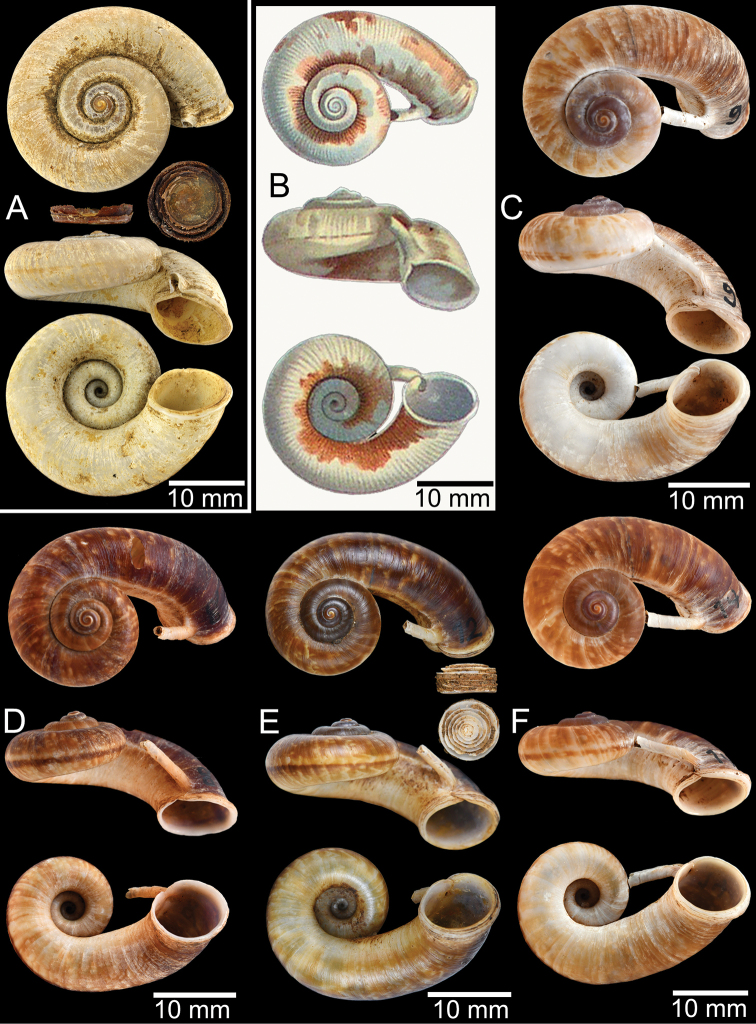
**A** specimen mentioned as being the syntype of “*Pterocyclosmarioni* Ancey, 1898” NMW 1955.158.24090. **B–F** shell of *Rhiostomamarioni***B** published figure of the type specimen (after Ancey 1898: pl. 9, fig. f.) and **C–F** specimens CUMZ 10210 from Feuang, Vientiane, Laos.

##### Distribution.

None of the *R.simplicilabre* specimens were collected from modern-day Cambodia; therefore, the distribution ranges of this species still need to be verified with specimens from accurate localities within Cambodia (Fig. 18).

In this study, *R.simplicilabre* has been determined to be mainly distributed in the northern part of Laos in Luang Phrabang and Xayabuli provinces, and in northern Thailand. In addition, the specimens recorded from Son La, Dien Bien, and Hao Binh provinces in northern Vietnam have been attributed to *R.housei*.

##### Remarks.

This species was described in the same publication by L. Pfeiffer (1862a), immediately after *R.hainesi* based on specimens in the collection of H. Cuming obtained from Henry Mouhot. The collection locality was brief “Camboja”; it is known that Mouhot’s recorded localities were imprecise and referred to broad geographical areas, for example, “Siam”, “Lao Mountains, Camboja” and “Camboja” (see Páll-Gergely et al. 2016a; Inkhavilay et al. 2019; Pholyotha et al. 2021). This has made it challenging to infer more precise type localities of several land snail species described from Mouhot’s specimens. According to the recorded itinerary of H. Mouhot (Mouhot 1864: 57; Ashburton 1864: map), land snail surveys from southern Cambodia could not find any specimens matching this species. The most recent works on land snails from southern Cambodia suggest the non-existence of *R.simplicilabre* (see Sutcharit et al. 2020). We have surveyed several limestone hills near Luang Phrabang areas, Laos, and encountered numbers of empty shells and living specimens that are well-matched with the type specimens of *R.simplicilabre* in all aspects (Fig. 23B–E). In addition, the specimens referred to as “*Rhiostomamarioni* (Ancey, 1898)” in Inkhavilay et al. (2019: fig. 11b) should be recognised as this species.

#### ﻿7. Rhiostoma marioni

Taxon classificationAnimaliaArchitaenioglossaCyclophoridae

(Ancey, 1898)

DD8864CB-7857-556E-8097-60FA2168DC7B

Figs 18 , 24B–F

Pterocyclos marioni (in part) Ancey, 1898: 137, pl. 9, fig. f. Type locality: Luang-prabang, Laos and Mont Hou, Tonkin. Fischer and Dautzenberg 1904: 430. Wood and Gallichan 2008: 64, pl. 25, fig. 3 (syntype).
Pterocyclus marioni —Kobelt 1911: 757.

##### Type material.

The figured specimens in Ancey (1898: pl. 9, fig. f) could not be located.

##### Other material examined.

**Laos**: Wat Non Hinhare, Feuang District, Vientiane Province: CUMZ 10210 (7 shells; Fig. 24C–F)

##### Diagnosis.

Shell medium, thick and depressed shell. Detached whorl long, ~2× longer than aperture width, curved, slightly descending. Breathing device tubular and straight. Shell colour uniformly brownish or with irregular blotches.

##### Differential diagnosis.

*Rhiostomamarioni* has been considered a junior synonym of *R.housei* from Thailand (Kobelt 1911). However, this species differs from *R.housei* in having a monochrome brownish shell colour, long detached whorl, and long straight tubular breathing device. In comparison, *R.housei* tends to have a brownish zigzag pattern, medium detached-whorl length, and a curved breathing tube.

##### Description.

***Shell*.** Shell medium, cW 17.1–20.5 mm, cH 9.1–10.6 mm, thick, and sub-discoidal to discoidal shape; detached-whorl length 14.0–20.5 mm. Apex acute; spire slightly elevated. Whorls 5 to 6, convex, increasing regularly; suture wide and deep; last whorl rounded and stout. Shell surface with fine growth lines. Periostracum thin corneous, and transparent. Shell colour uniformly brownish to dark brown (rarely with brownish blotches); ventral shell surface with paler colour; with narrow and dark spiral band on periphery. Detached whorl long, ~ 2× longer than apertural width, curved and descending. Peristome circular and double; lip thickened, expanded and multi-layered. Aperture opened sub-ventrally. Breathing device tubular and its tip often attached to preceding whorl; outer lip forming a long, straight, closed tube, located just behind apertural lip; inner lip with small hole inside aperture. Umbilicus widely opened and deep. Operculum calcareous, cup-shaped, and multispiral (Fig. 24B–F).

##### Distribution.

This species is currently known only from Vientiane Province, Laos (Fig. 18).

##### Remarks.

This species was described based on two specimen lots from “Luang-prabang, Laos” and “Mont Hou, Tonkin” [probably in the area of Muang Khua and Yot Ou Districts, Phongsaly Province, Laos (see Pavie 1903: pl. 1)]. The original description includes an illustration of a single specimen and one set of measurements (Ancey 1898). Unfortunately, this figured specimen could not be located from the RBINS or NMW collection. The specimen lot NMW 1955.158.24090 consisting of a single specimen (with operculum) that has Ancey’s type label and collection locality “Luang-prabang” was considered the possible holotype by Wood and Gallichan (2008: pl. 25, figs 3, iv). However, this surviving specimen looks different from the actual illustrated specimen in the original publication. This specimen has a flattened shell, short detached whorl, and notch-shaped breathing device projecting anteriorly (Fig. 24A). Although the operculum is not clearly mentioned in the original description, the operculum accompanied by this specimen lot (NMW 1955.158.24090) is dark corneous, plate-shaped and multispiral with elevated lamellae (Fig. 24A). It is distinct from the typical operculum of *Rhiostoma* (Fig. 2), and more closely resembling the *Ptychopoma* or *Scabrina.* This specimen is contradicting to Ancey’s concept of “*Pterocyclosmarioni*”, whereas the species description agrees well with the specimen illustration (Ancey 1898: pl. 9, fig. f). Therefore, we consider specimen NMW 1955.158.24090 (Fig. 24A; Inkhavilay et al. 2019: fig. 11a; Do et al. 2020b: fig. 1d) as a distinct species. We have excluded that specimen from our attention in the type series.

This redescription is based on the recently collected specimens from Feuang District, Vientiane Province, Laos. Before establishing the French Protectorate of Laos in 1893 (Pavie 1903: pl. 6; Tips 1999: 131), the historical name “Luang Prabang” generally referred to the city or the Kingdom Luang Prabang (during the 18^th^ to 19^th^ century). This name encompasses a wide geographical area, mainly present-day northern Laos: Bokeo, Houa Phanh, Luang Namtha, Luang Prabang, Oudomxay, Phongsaly and Vientiane provinces. Therefore, this newly collected specimen from Vientiane Province is considered the topotypic specimen of this species. This specimen lot possesses a long detached whorl and a long tubular-shaped breathing device identical to the unique characteristic of *R.marioni*; it agrees well with the illustration (Fig. 24B) and the species description.

The previous records under the name *Rhiostoma* “*marioni*” from Lai Chu, Son La, Dien Bien and Lau Cai provinces, North Vietnam, as mentioned by Do et al. (2020b), should be referred to another species (see under *R.abletti*).

#### ﻿8. Rhiostoma dalyi

Taxon classificationAnimaliaArchitaenioglossaCyclophoridae

Blanford, 1902

970B9754-C196-5BA0-85A5-B629474EDB39

Figs 18 , 25

Rhiostoma dalyi Blanford, 1902: 34, 35, fig. 1. Type locality: Juxta Phitsanulok, in sylvis humidis etr densis [Phitsanulok Province, Thailand]. Blanford 1903: 281. Fischer and Dautzenberg 1904: 427. Solem 1966: 11. Sutcharit et al. 2019: 21, fig. 4i, j.
Rhiostoma housei —Tumpeesuwan 2001: 41–46, figs 4.10–4.12 (misidentified).

##### Type material.

**Figure 25.**
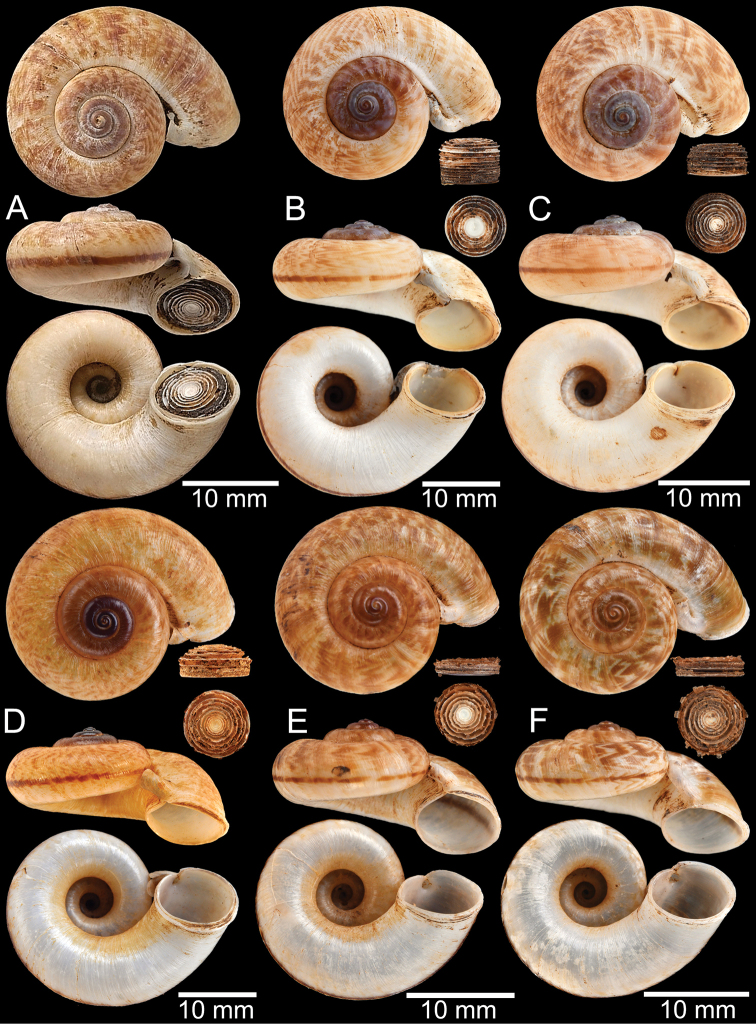
Shell of *Rhiostomadalyi***A** syntype NHMUK 1902.1.24.14–16 from Juxta Phitsanulok **B, C** specimens CUMZ 4848 from Tham Erawan, Long, Prae **D** specimen CUMZ 3979 from Tarnsawan Waterfall, Phayao **E, F** specimens CUMZ 3926 from Tham Pha Tangyai, Pong, Phayao.

***Syntype***NHMUK 1902.1.24.14–16 (3 shells; Fig. 25A) from Pitsunaloke, Siam. This syntype lot in the Blanford ex. Daly collection consists of three specimens; the figured specimen in the original description can be recognised by its curved breathing device and is figured herein.

##### Other material examined.

**Thailand**: Tham Pha Dang, Chiangkham, Phayao: CUMZ 4462, 4729. Doi Phunang, Dokkamtai, Phayao: CUMZ 3938, 3942, 4313, 4319, 4420. Tarnsawan Waterfall, Dokkamtai, Phayao: CUMZ 3824, 3826, 3977 (Fig. 25D). Tham Pha Tangyai, Pong, Phayao: CUMZ 3926 (Fig. 25E, F), 4784. Tham Pha Tung, Pong, Phayao: CUMZ 10062. Ban Tha Sri, Muang, Lampang: CUMZ 4728. Pratupha, Ngoaw, Lampang: CUMZ 4463. 3 km. before the junction to Tham Sereethai, Long, Prae: CUMZ 4450. Hill Near Tham Air Tammachard, Long, Prae: CUMZ 3979, 4451. Tham Air Thammachad, Ban Pan, Long, Prae: CUMZ 4762. Tham Erawan, Long, Prae: CUMZ 4792, 4848 (Fig. 25B, C), 10063, 10064. Wat Tham Pha Nang Koy, Rong Kwang, Prae: CUMZ 3884, 3924, 4726.

##### Diagnosis.

Shell large, thick, and depressed. Detached whorl medium in length, and slightly descending. Breathing device tubular, curved, and attached to preceding whorl. Shell whitish with brownish zigzag patterns.

##### Differential diagnosis.

This species is very similar to *R.housei* from central Thailand. The distinguishing characteristics of *R.dalyi* are a stout last whorl and a tubular-shaped breathing device that is short, curved and attached to the preceding whorl. In comparison, *R.housei* has a slender last whorl, and a long and straight tubular breathing device. In addition, this species also differs from *R.trigina* sp. nov. in having a stout and short detached last whorl, a short and curved tubular breathing device, and a ventral shell surface usually without a pattern. In contrast, *R.trigina* sp. nov. possesses a slender detached whorl, long and straight tubular breathing device, and usually with brown zigzag patterns on both sides.

##### Description.

***Shell*.** Shell medium to large, cW 19.9–24.6 mm, cH 9.7–13.4 mm, thick, and discoidal shape; detached-whorl length 7.5–14.0 mm. Apex acute with dark colouration; spire slightly elevated. Whorls 5 to 6, convex and increasing regularly; suture wide and deep; last whorl rounded and stout. Shell surface with fine growth lines. Periostracum thick or thin corneous and transparent. Shell colour whitish with brownish zigzag pattern; ventral shell surface usually without pattern; with narrow and dark brown spiral band on periphery. Detached whorl of medium length, approximately the same length as apertural width. Peristome circular and double; lip thickened, slightly expanded and multi-layered. Aperture opened sub-laterally. Breathing device tubular, curved and its tip attached to preceding whorl; outer lip forming a short and closed tube, and located just behind apertural lip; inner lip with hole inside aperture. Umbilicus widely opened and deep. Operculum calcareous, cup-shaped, and multispiral (Fig. 25).

##### Distribution.

This species is distributed in the northern part of Thailand in Phitsanuloke (type locality), Lampang, Phrae and Phayao provinces (Fig. 18).

##### Remarks.

Solem (1966) record the specimens from ‘Chieng Dao’ as this species. However, we examined a number of specimens from Chieng Dao, Chieng Mai Province and found clearly distinct shell morphology compared to the type specimen of *R.dalyi*. In addition, the COI barcoding suggests that *R.dalyi* and *R.trigina* sp. nov. are separate species (Fig. 3). Therefore, the record of this species by Solem (1966) should be referred to *R.trigina* sp. nov.

#### ﻿9. Rhiostoma jalorensis

Taxon classificationAnimaliaArchitaenioglossaCyclophoridae

Sykes, 1903

775B1D12-12E7-5CA5-AA0D-F4E622975446

**Figure 26.**
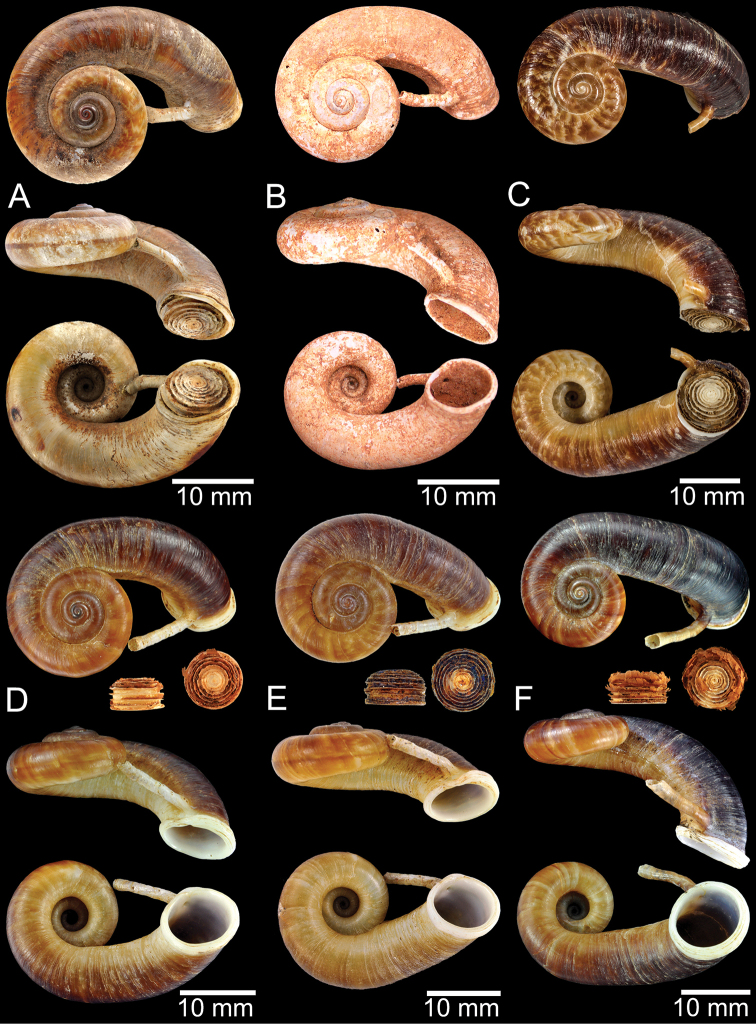
Shell of *Rhiostomajalorensis***A** holotype UMCZ 1030 from Biserat, Jalor **B** paratype NMW 2.1981.118.02760 from type locality **C** holotype of “*Rhiostomahuberi* Thach, 2018” MNHN-IM-2000-34049 from Ao Luc, South Thailand **D, E** specimens CUMZ 3994 from Wat Tham Sue, Krabi **F** specimen CUMZ 4387 from Tham Nam Pud, Phang Nga. Photograph: M Caballer and V Heros, MNHN (**C**).

**Figure 27.**
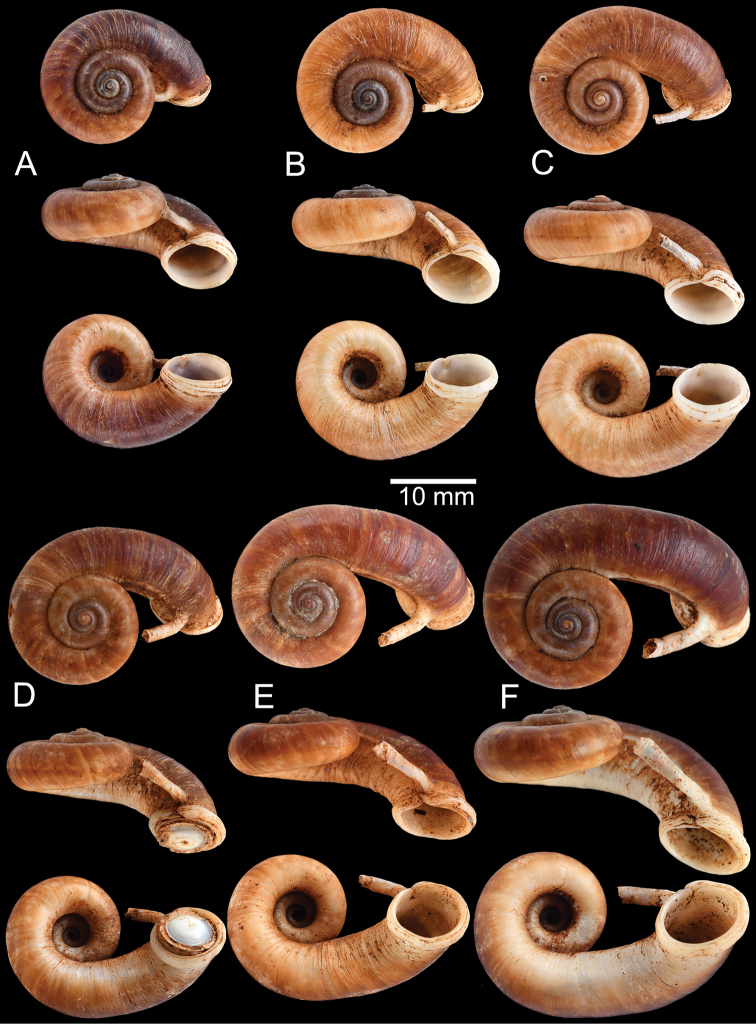
Shell of *Rhiostomajalorensis***A–C** specimens CUMZ 4843 from Wat Tham Suwannakhuha, Phang Nga shows variation of the shell size and length of detached whorl **D–F** specimens CUMZ 4483 from Tao Thong Waterfall, Phang Nga shows variation in length of the breathing devices.

Figs 17D , 18 , 26 , 27 , 32C

Rhiostoma jalorensis Sykes, 1903: 196, pl. 20, figs 6–8. Type locality: Limestone hills and caves, Biserat, Jalor [=Yala Province, Thailand]. Salisbury 1949: p1. 3B, figs 1, 2. Tumpeesuwan 2001: 47–52, figs 4.13–4.15. Tarruella and Doménech 2010: 190, fig. 2a, b. Kongim et al. 2013b: 16, fig. 2h.
Rhiostoma jalorense —Laidlaw 1928: 31.
Rhiostoma cf. smithi —Hemmen et al. 1999: 10, 11, figs 7, 9, 10 (misidentified).
Rhiostoma smithi —Hemmen and Hemmen 2001: 38, fig. 4 (misidentified). Patamakanthin 2001: 222, 223, figs f1–f3, i1–i4, j1–j4, l1–l3 (misidentified).
Rhiostoma huberi Thach, 2018: 17, figs 68–70. Type locality: Ao Luc, South Thailand [Ao Luek District, Krabi Province]. New synonym.

##### Type material.

***Holotype***UMZC I.100155 (Fig. 26A) from Biserat, State of Jalor, Malay Peninsula and ***paratype***NMW 2.1981.118.02760 (1 shell; Fig. 26B). ***Holotype***MNHN-IM-2000-34049 (Fig. 26C) of *Rhiostomahuberi* Thach, 2018, from Ao Luc, South Thailand.

##### Other material examined.

**Thailand**: Klong Seang Wildlife Sanctuary, Ban Ta Khun, Surat Thani: CUMZ 4323. Nature Trail, Khaosok, Ban Ta Khun, Surat Thani: CUMZ 4478, 4723. Ratchaprapha Dam, Ban Ta Khun, Surat Thani: CUMZ 4894. Anurak Resort, Panom, Surat Thani: CUMZ 10142. Tham Nam Lod, Panom, Surat Thani: CUMZ 10145. Ao Phang-nga, Muang, Phang Nga: CUMZ 4327. Khao Prao, Muang, Phang Nga: CUMZ 4327. Phang-nga Bay, Muang, Phang Nga: CUMZ 4325, 4326. Wat Tham Suwannakhuha, Takua Thung, Phang Nga: CUMZ 3837, 3848, 4328, 4329, 4821, 4843 (Fig. 27A–C). Tham Nam Pud, Muang, Phang Nga: CUMZ 3836, 3839, 3841, 3899, 4387 (Fig. 26F), 10136. Tham Kiriwong (Tham Kob), Thap Put, Phang Nga: CUMZ 3822, 3823, 3832, 4484, 4485, 4850, 10143. Tao Thong Waterfall, Thap Put, Phang Nga: CUMZ 3817, 3829, 3845, 4386, 4482, 4483 (Fig. 27D–F), 4798, 4837, 4858, 4873, 10141. On the way to Than Bok Khorani, Ao Luek, Krabi: CUMZ 10144, 10146. Tham Na Mee, Ao Luek, Krabi: CUMZ 10137. Tham Phet, Ao Luek, Krabi: CUMZ 3831. Tham Sra Yoon Thong, Ao Luek, Krabi: CUMZ 3819. Tham Chang Sri, Muang, Krabi: CUMZ 4330. Tham Lang Rong Rian, Ban Tubprik Primary School, Muang, Krabi: CUMZ 4487. Wat Tham Sua, Muang, Krabi: CUMZ 3834, 3994 (Figs 17D, 26D, E), 4331, 4486, 4859, 10138, 10139, 10140.

##### Diagnosis.

Shell medium, thick and depressed. Detached whorl long, curved and descending. Breathing device tubular. Shell colour usually uniformly brown to dark brown and with or without peripheral band.

##### Differential diagnosis.

*Rhiostomajalorensis* can be distinguished from *R.ebenozostera* sp. nov. in having longer detached whorl, uniformly dark brown shell colour, and narrow peripheral band. In contrast, the new species has a shorter detached whorl, dark brown blotches shell colour, and with broad and dark brown peripheral band. In addition, the geographically disjunct distributions suggest that they are distinct species.

##### Description.

***Shell*.** Shell small to medium, cW 17.1–20.6 mm, cH8.8–11.8 mm, slightly thick, and sub-discoidal shape; detached-whorl length 12.5–19.5 mm. Apex acute; spire slightly elevated. Whorls 5 to 6, convex, increasing regularly; suture wide and deep; last whorl rounded. Shell surface with fine growth lines. Shell colour uniformly brown, dark brown to black (rarely with zigzag pattern); ventral shell surface with paler colour; thin and narrow peripheral band sometimes present. Periostracum thick or thin corneous and transparent. Aperture opened sub-ventrally to ventrally. Detached whorl long, ~ 2× longer than apertural width, curved and descending. Peristome circular and double; lip thickened, expanded, and multi-layered. Breathing device tubular, straight and its tip usually attached to preceding whorl (sometimes not attached); outer lip forming a long, straight and closed tube, and located just behind apertural lip; inner lip with small hole inside aperture. Umbilicus widely opened and deep. Operculum calcareous, cup-shaped, and multispiral (Figs 26, 27).

***Radula*.** Teeth arrangement and shape are similar to *R.housei*. Central tooth with large central cusp and two lateral cusps on each side. Lateral teeth composed of four cusps; central cusp large and triangular; two inner cusps with pointed tips and tapering in size; one outer cusp small. Marginal teeth each composed of three cusps (Fig. 32C).

##### Distribution.

The previous records of this species were from the type locality, Biserat, Jalor (former name of Yala Province, Thailand). The records from Koh Si-Hah, Singgora (now in Phatthalung Province, Thailand) by Laidlaw (1928) and Salisbury (1949) need to be verified with new collections. The recent collections are mainly from the western part of peninsular Thailand in Surat Thani, Phang Nga and Krabi provinces (Fig. 18).

##### Remarks.

*Rhiostomajalorensis* tends to have high variation in both shell form and shell colour. For example, the specimens from Tham Nam Pud, Phang Nga (Fig. 26F) exhibit relatively large shells with long and descending detached whorl (dL = 12.0–25.0 mm, 19.28±3.43 mm), dark shell colour, and aperture opened sub-ventrally to ventrally. On the other hand, the population from Wat Suwankuha, Phang Nga (Fig. 27A–C) tend to have small shells with short detached whorl (dL = 9.0–14.5 mm, 11.67 ± 1.31 mm), pale brown to dark shell colour, and aperture opened sub-laterally. The specimens from Tao Tong Waterfall, Phang Nga (Fig. 27D–F) have medium to long descending detached whorl, brownish shell colour, and aperture opened sub-ventrally. However, the unique attributes of descending detached whorl, tubular-shaped breathing device and monochrome brown to black shell colour suggest that these are morphological variations.

The holotype of *Rhiostomahuberi* Thach, 2018 from Krabi Province is in an early stage of maturation, as the shell has a thin and sharp apertural lip. This specimen has a long and descending detached whorl, a brownish zigzag pattern on the coiled whorl, tubular-shaped breathing device, and aperture opened ventrally. In the absence of continuous characters in other populations, this extreme example would undoubtedly be recognised as a distinct species. However, the COI analysis clearly suggests that specimens from the entire distribution range and with these shell variations belong to a single species (Fig. 3). Therefore, we formally synonymise *Rhiostomahuberi* Thach, 2018 with *R.jalorensis*.

#### ﻿10. Rhiostoma thachi

Taxon classificationAnimaliaArchitaenioglossaCyclophoridae

Huber, 2018

F27659C3-EB87-5707-AAA7-679EE4CB9D7D

Figs 18 , 28

Rhiostoma thachi Huber in Thach, 2018: 17, 18, figs 74–80. Type locality: Thakhek, Central Laos (close to Vietnam borderline).
Rhiostoma haughtoni —Thach 2018: 17, figs 66, 67 (misidentified).

##### Type material.

**Figure 28.**
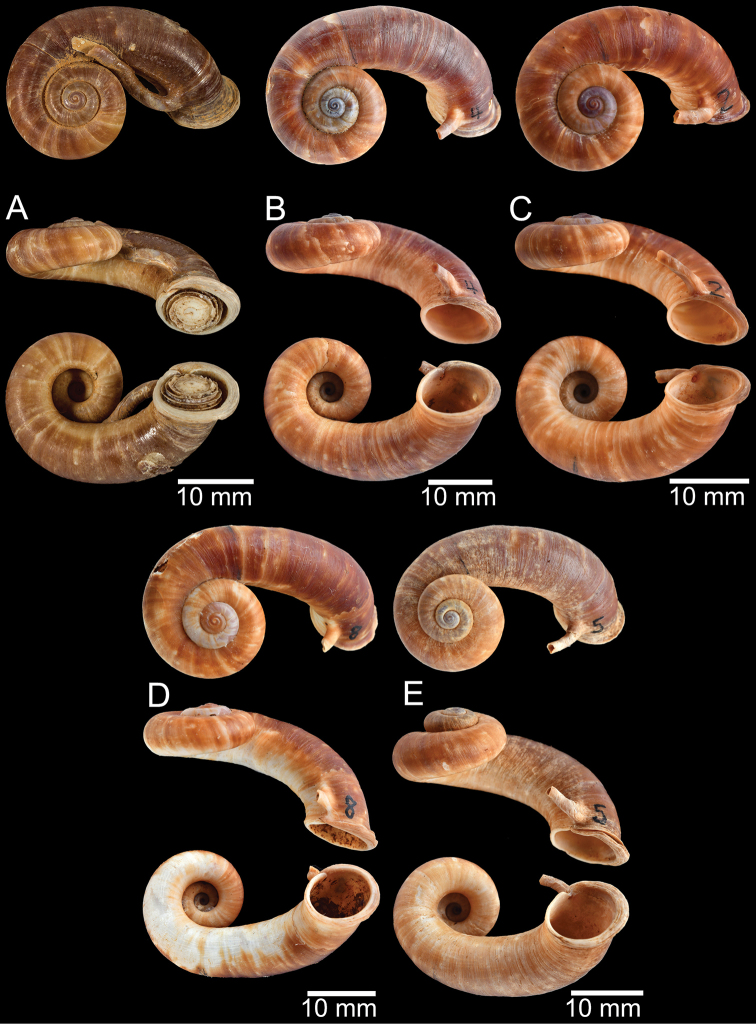
Shell of *Rhiostomathachi***A** holotype MNHN-IM-2000-34050 from Thakhek, Laos **B–E** specimen CUMZ 3941 from Vang Vieng, Vientiane, Laos (**E** with malform). Photograph: M Caballer and V Heros, MNHN (**A**).

***Holotype***MNHN-IM-2000-34050 (Fig. 28A) from Thakhek, Central Laos.

##### Other material examined.

**Laos**: Tam Jung (cave), Vang Vieng District, Vientiane Province: CUMZ 3941 (8 shells; Fig. 28B–E), CUMZ 10201 (5 shells).

##### Diagnosis.

Shell medium, thick and depressed shell. Detached whorl long, curved and descending, and sutural ridge absent. Breathing device very long, tubular. Shell colour uniformly brown to dark brown along dorsal and ventral sides. Peripheral band absent. Apertural lip much expanded at palatal side, double, with many concentric lines.

##### Differential diagnosis.

*Rhiostomathachi* can be distinguished from *R.jalorensis* from peninsular Thailand in having a very long detached whorl, without peripheral band, and lip much expanded at palatal side. In comparison, *R.jalorensis* has a shorter detached whorl, with peripheral band, and lip evenly thickened and expanded.

##### Description.

***Shell*.** Shell medium, cW 17.2–21.4 mm, cH 8.8–11.9 mm, thick, and nearly flattened to sub-discoidal shape; detached-whorl length 19.5–27.0 mm. Apex acute; spire slightly elevated. Whorls 5 to 6, convex, increasing regularly; suture wide and deep; last whorl rounded and stout. Shell surface with fine growth lines. Periostracum thin corneous, and transparent. Shell colour uniformly brown to dark brown; ventral shell surface with paler colour; peripheral band absent. Detached whorl long, ~ 2× longer than apertural width, curved and descending, and sutural ridge inconspicuous. Peristome circular and double; lip brownish, thickened, much expanded on palatal side, and multi-layered. Aperture opened sub-laterally to ventrally. Breathing device tubular, and may be attached to preceding whorl; outer lip forming a long and complete tube, located just behind apertural lip; inner lip with hole inside aperture. Umbilicus widely opened and deep. Operculum calcareous, cup-shaped, and multispiral (Fig. 28).

##### Distribution.

This species is known from the type locality, Khammouane Province, and a limestone mountain in Vientiane Province (Fig. 18).

##### Remarks.

The empty shells from Vang Vieng District, Vientiane Province, Laos, are relatively bleached and have no operculum. Instead, they have a long, curved and descending detached whorl; tubular-shaped breathing device; aperture opened ventrally; apertural lip brownish, thickened and expanded. However, these unique shell characters suggest they are conspecific with *R.thachi* from Central Laos. No living specimens of this species were found during this survey. Therefore, more sampling effort and molecular phylogenetic analyses are required to demonstrate the relationship between this species and other species in the *R.housei* group.

The specimen mentioned as *R.haughtoni* in Thach (2018: 17, figs 66, 67) is absolutely different from the type specimens from Myanmar (Fig. 11A, B). In addition, this specimen agrees well with all shell characters and was also collected from an area near the *R.thachi* type locality. Therefore, this specimen is misidentified and should be recognised as *R.thachi*.

#### ﻿11. Rhiostoma ebenozostera

Taxon classificationAnimaliaArchitaenioglossaCyclophoridae

Tongkerd & Panha sp. nov.

814D1AE6-5F96-583C-ABAA-FB3CC5E041A7

https://zoobank.org/24A470B8-4449-4FF9-802F-AC4DC0BCBB11

Figs 18 , 29 , 32D

##### Type material.

**Figure 29.**
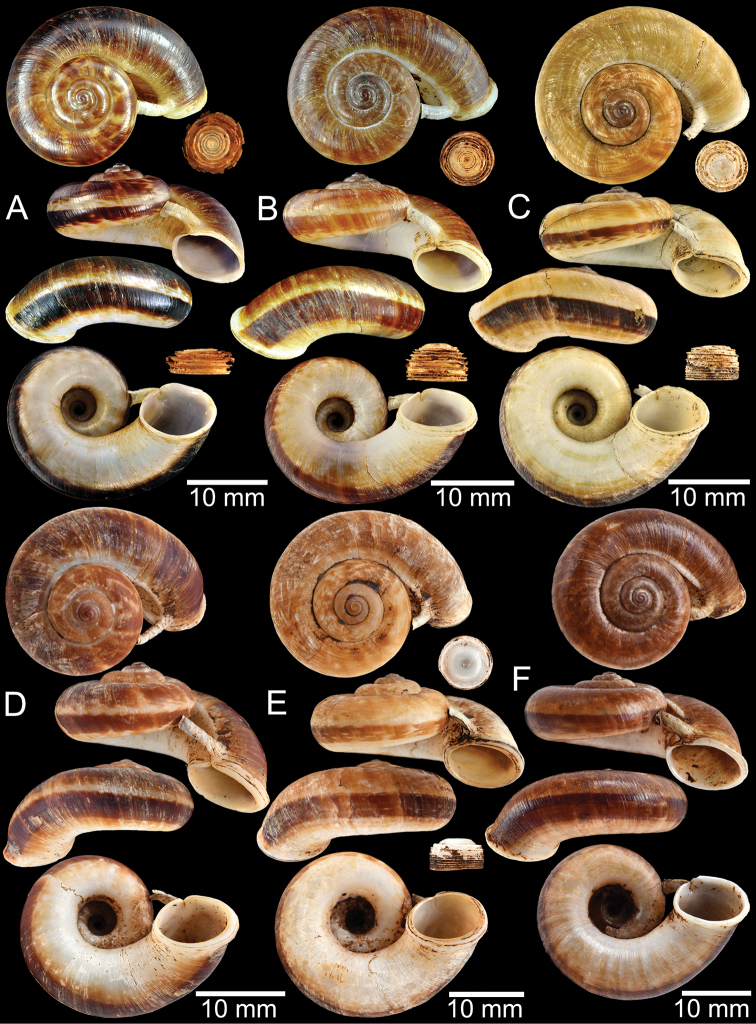
Shell of *Rhiostomaebenozostera* sp. nov. **A** holotype CUMZ 4703 from Tham Pha Poo, Loei **B, C** paratypes from type locality **B** paratype CUMZ 4704 and **C** paratype CUMZ 3889 **D** specimen CUMZ 10211 from Lom Kao, Phetchabun **E** specimen CUMZ 4765 from Suwannakhuha, Nongboa Lumphu **F** specimen CUMZ 10166/2 from Phu Wiang, Khon Kaen.

***Holotype***CUMZ 4703 (cW 19.4 mm, cH 11.1 mm, dL 13.7 mm; Fig. 29A). ***Paratypes***CUMZ 3887 (228 shells), CUMZ 3889 (53 adults + 25 juveniles; Fig. 29C), CUMZ 4704 (2 shells; Figs 29B, 32D), CUMZ 4766 (99 adults + 24 juveniles), NHMUK 20220439 (5 shells), and SMF 368674 (5 shells). All paratypes are from the type locality.

##### Type locality.

Tham Pha Poo, Muang District, Loei Province, Thailand (17°34'45.1"N, 101°42'36.2"E). Dry dipterocarp forest on limestone hill.

##### Other material examined.

**Thailand**: Wat Tham Pha Ban, Chiangkan, Loei: CUMZ 4444. Phu Pha Lom, Muang, Loei: CUMZ 10013. Wat Tham Phu Pha Lom, Muang, Loei: CUMZ 4443. Tham Khuha Waree, Wang Sapung, Loei: CUMZ 3925, 3946. Tham Pha Fang, Wang Sapung, Loei: CUMZ 10155. Khao Wangpha, Na Wang, Nongboa Lumphu: CUMZ 3935. Tham Suwannakhuha, Na Wang, Nongboa Lumphu: CUMZ 4765 (Fig. 29E). Wat Paphaya, Suwannakhuha, Nongboa Lumphu: CUMZ 10031. Wat Suwannakhuha, Ban Nasi, Suwannakhuha, Nongboa Lumphu: CUMZ 3889, 3933, 10021. Wat Phu Poon, Lomkao, Phetchabun: CUMZ 10211 (Fig. 29D). Tham Poo Loup, Chum Phae, Khon Kaen: CUMZ 3931. Tham Nennoi, Chum Phae, Khon Kaen: CUMZ 10015. Tham Phayanakarat, Chum Phae, Khon Kaen: CUMZ 10025. Phu Wiang, Wiang Kao, Khon Kaen: CUMZ 10166/1, 10166/2 (Fig. 29F).

##### Diagnosis.

Shell medium, thick and depressed. Detached whorl medium in length, curved, and slightly descending. Breathing device tubular. Broad dark brown spiral band on periphery present.

##### Differential diagnosis.

*Rhiostomaebenozostera* sp. nov. can be distinguished from all other recognised *Rhiostoma* species by having a prominent wide dark brown to black peripheral band. This colour pattern persists in all populations recognised for this species. Regardless of the peripheral band, this new species can be distinguished from *R.housei* in having straight and slender tubular breathing device, and relatively longer detached whorl. In contrast, *R.housei* has a stout and curved tubular breathing device and relatively shorter detached whorl.

##### Description.

***Shell*.** Shell medium, cW 17.7–22.6 mm, cH 9.4–12.1 mm, thick, and sub-discoidal to discoidal shape; detached-whorl length 8.5–14.5 mm. Apex acute; spire nearly flat to elevated. Whorls 4 to 5, convex, increasing regularly; suture wide and shallow; last whorl rounded and stout. Detached whorl medium in length, approximately the same length as apertural width, curved and descending. Shell surface with fine growth lines. Periostracum thin, corneous, and transparent. Shell colour uniformly brownish or with pattern of dark brown blotches, and faded on ventral shell surface; dark spiral band on periphery always present and broad. Peristome circular and double; lip thickened, expanded and multi-layered. Aperture opened sub-ventrally. Breathing device tubular, perpendicular to detached whorl and its tip attached to preceding whorl; outer lip forming long and closed tube and located just behind apertural lip; inner lip with deep incision or small hole inside aperture. Umbilicus widely opened and deep. Operculum calcareous, cup-shaped, and multispiral (Fig. 29).

***Radula*.** Teeth arrangement and shape are similar to those of *R.housei*. Central tooth with large central cup and two lateral cusps on each side. Lateral teeth composed of three cusps; central cusp large, with convex tip, and flanked by small pointed tips of one outer cusp and one inner cusp. Marginal teeth each consisting of three pointed cusps (Fig. 32D).

##### Etymology.

The specific name *ebenozostera* is derived from the two Greek words *ebenos* meaning black colour, and *zoste* meaning girdle or belt. This name refers to the prominence of a dark, wide, peripheral band, which is the distinguishing character of this species.

##### Distribution.

This new species has narrow distribution; it is known from several limestone outcrops in dry deciduous forests of Loei, Nong Bua Lam Phu, and Khonkaen provinces (Fig. 18).

##### Remarks.

The specimens from Nong Bua Lam Phu possess a stout shell, slightly shorter detached whorl, and paler shell colour (Fig. 29E) than the typical population. However, a prominent broad, dark, peripheral band suggests that they are conspecific.

#### ﻿12. Rhiostoma lannaense

Taxon classificationAnimaliaArchitaenioglossaCyclophoridae

Tongkerd & Tumpeesuwan sp. nov.

7F939DA8-F9B0-5A34-B8D7-FBF6186DE2DE

https://zoobank.org/4DF8B280-2F75-4FFF-B4CD-3287CDCD3309

Figs 18 , 30 , 32E

Rhiostoma sp. 1—Tumpeesuwan 2001: 59–64, figs 4.19–4.21 (in part).

##### Type material.

**Figure 30.**
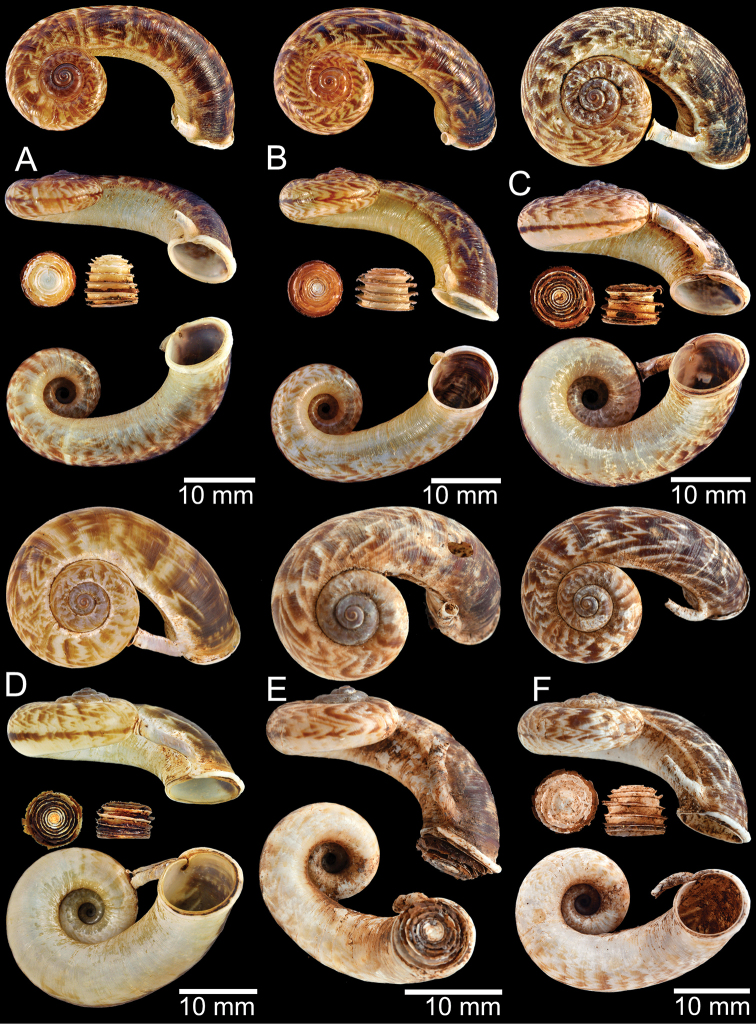
Shell of *Rhiostomalannaense* sp. nov. **A** holotype CUMZ 4500 from Pingkong, Chiang Dao, Chiang Mai **B** paratype CUMZ 4701 from type locality **C, D** paratypes CUMZ 4721 from type locality **E** specimen CUMZ 10043 from Pang Mapha, Maehongsorn **F** specimen CUMX 10041 from Chai Prakan, Chiang Mai

***Holotype***CUMZ 4500 (cW 17.1 mm, cH 8.1 mm, dL 35.0 mm; Fig. 30A). ***Paratypes***CUMZ 3910 (40 shells), CUMZ 4350 (1 shell), CUMZ 4701 (1 shell; Figs 30B, 32E), CUMZ 10037 (8 adults + 30 juveniles), CUMZ 10038 (5 shells), NHMUK 20220440 (5 shells), and SMF 368675 (5 shells). All paratypes are from the type locality.

##### Type locality.

Ban Ping Klong (village), Chiangdao District, Chiang Mai Province, Thailand (19°30'48.6"N, 99°03'21.1"E). Small limestone hills covered by dry deciduous forest.

##### Other material examined.

**Thailand**: Tham Sam Ta, Muang, Maehongsorn: CUMZ 4804. 1 km. from the junction to Tham Mae Ra Na, Pang Mapha, Maehongsorn: CUMZ 4440, 4464. Pang Mapha, Maehongsorn: CUMZ 4341. Tham Mae Lana, Pang Mapha, Maehongsorn: CUMZ 4702 (Fig. 30C, D), 10042, 10043 (Fig. 30E). Tham Pha Mon, Pang Mapha, Maehongsorn: CUMZ 4343. Tham Phadeang, Pang Mapha, Maehongsorn: CUMZ 10040. Wat Pa Tham Wua, Pang Mapha, Maehongsorn: CUMZ 10036. Tham Tabtao, Chai Prakarn, Chiang Mai: CUMZ 3912, 10041 (Fig. 30F). Chaiprakarn, km. 43 reach Chiang Dao, Chiang Mai: CUMZ 4738. Km. 93+200 m. Tham Klap, Pingkong, Chiang Dao, Chiang Mai: CUMZ 3908, 10039. Pa Sak Ngam, Doi Saket, Chiang Mai: CUMZ 4754. Huai Nam Dang, Mae Tang, Chiang Mai: CUMZ 10033.

##### Diagnosis.

Shell small to medium, thin, and flattened to depressed shell. Detached whorl long, slender, curved, and descending. Breathing device tubular. Shell colour with dark brown zigzag patterns. Operculum calcareous, tall cup-shaped with loose lamellae.

##### Differential diagnosis.

*Rhiostomalannaense* sp. nov. differs from *R.marioni*, *R.thachi*, and *R.jalorensis* in having a whitish shell with a dark brown zigzag pattern and longer detached whorl. In contrast, these three species exhibit uniformly brownish to dark brown shells and generally without a colour pattern and a relatively shorter detached whorl. In addition, *R.thachi* has a broader expanded aperture on the palatal side, while this new species has a thickened but not expanded lip.

This new species is superficially similar to *R.tigrina* sp. nov., but it can be distinguished by having very long and curved detached whorl, aperture opened ventrally, and tall cup-shaped operculum with looser lamellae. In contrast, the latter species has a shorter detached whorl, aperture opened sub-ventrally and low cup-shaped operculum with denser lamellae. Although these two species are mainly distributed in northern Thailand, the COI barcoding shows high genetic diversity (Appendix 1: Table A1) and divided *R.lannaense* sp. nov. as a distinct clade from other northern species, which suggests they are distinct species (Fig. 3).

##### Description.

***Shell*.** Shell small to medium, cW 14.5–19.3 mm, cH 7.3–10.1 mm, thin, and nearly flattened to sub-discoidal shape; detached-whorl length 19.5–28.5 mm. Apex acute with dark colouration; spire convex to nearly flat. Whorls 4 to 5, convex, increasing regularly; suture wide and shallow; last whorl rounded and slender. Shell surface with fine growth lines. Periostracum corneous and transparent. Shell colour with brown to dark brown zigzag pattern and faded on ventral shell surface; narrow black spiral band on periphery. Detached whorl long, ~3× longer than aperture width, curved, and descending. Peristome circular and double; lip thickened, slightly expanded and multi-layered. Aperture opened sub-ventrally to ventrally. Breathing device tubular and its tip sometimes attached to preceding whorl; outer lip forming a short to long and closed tube, and located just behind apertural lip; inner lip with deep incision or small hole inside aperture. Umbilicus widely opened and deep. Operculum calcareous, tall cup-shaped, and multispiral with loose lamellae (Fig. 30).

***Radula*.** Teeth arrangement and shape are similar to those of *R.housei*. Central tooth with large pointed central cusp; two lateral cusps on each side with small pointed tips. Lateral teeth consisting of four cusps; central cusp large, with dull tip, and flanked by two inner cusps and one small outer cusp. Marginal teeth each consisting of three pointed cusps (Fig. 32E).

##### Etymology.

The specific name *lannaense* is derived from the historical name of the Lan Na Kingdom, which flourished approximately from the 13^th^ to 18^th^ centuries. It refers to the distribution range of this new species in the northern part of Thailand, which is the approximate centre of the Lan Na Kingdom.

##### Distribution.

This new species has a narrow distribution range in a few localities in Chiang Mai and Maehongsorn provinces (Fig. 18).

##### Remarks.

There are two morphotypes occurring in this species. The typical morphotype has a long and curved detached whorl, and the tubular breathing device does not reach the preceding whorl. The shorter morphotype has a short, detached whorl, and the tubular breathing device reaches the preceding whorl (Fig. 30D). These two morphotypes are sympatric, and also have brownish zigzag colour patterns and a tall cup-shaped operculum with loose lamellae. However, the shell morphologies together with COI barcoding suggest these are conspecific.

#### ﻿13. Rhiostoma tigrina

Taxon classificationAnimaliaArchitaenioglossaCyclophoridae

Tongkerd & Tumpeesuwan sp. nov.

9B03C93A-9D3C-54BD-9C53-245AE285A411

https://zoobank.org/94D81E56-7DAB-469E-B93E-0C18C59B7999

Figs 18 , 31 , 32F

Rhiostoma sp. 1—Tumpeesuwan 2001: 59–64, figs 4.19–4.21 (in part).

##### Type material.

**Figure 31.**
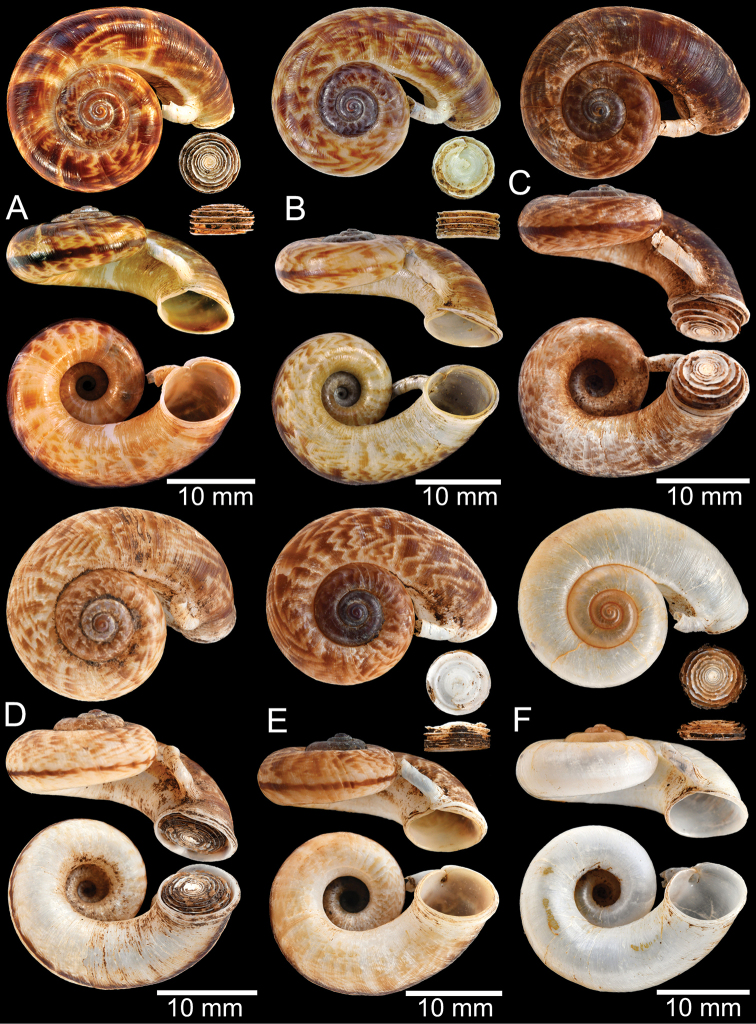
Shell of *Rhiostomatigrina* sp. nov. **A** holotype CUMZ 4497/1 from Tham Pum Tham Pla, Mae Sai, Chiang Rai **B** paratype CUMZ 4497/2 from type locality **C** paratype CUMZ 4806 from type locality **D** specimen CUMZ 4795 from Tham Phra, Chiang Rai **E, F** specimens CUMZ 4807 Mae Suai, Chiang Rai

**Figure 32.**
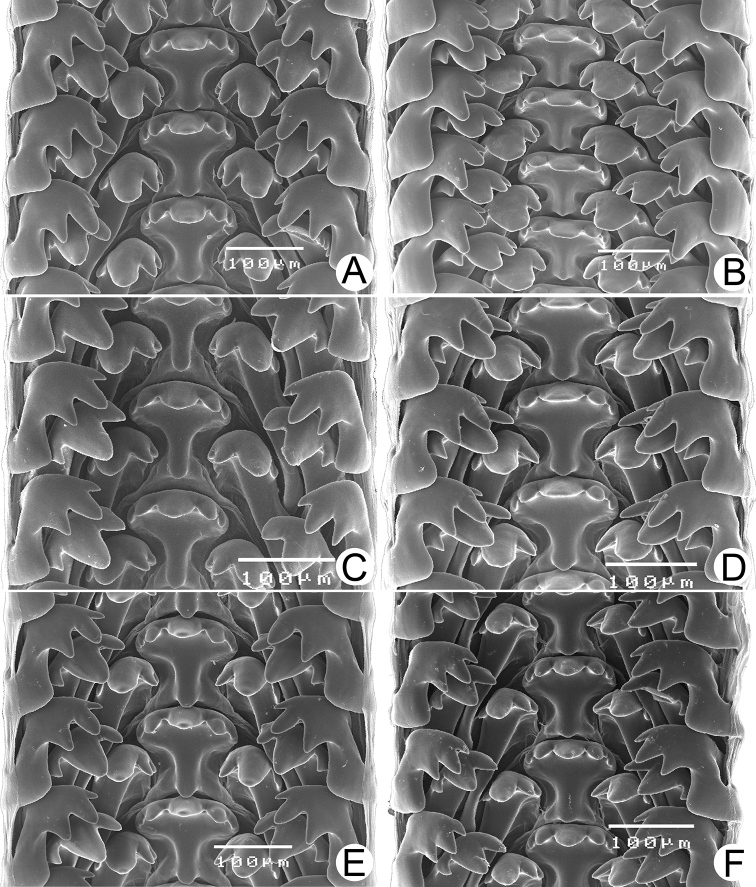
Radula morphology **A***Rhiostomahousei* specimen CUMZ 3982 from Wat Tham Srivilai, Saraburi **B***Rhiostomahainesi* specimen from Chanthaburi **C***Rhiostomajalorensis* specimen CUMZ 3994 from Wat Tham Sua, Krabi **D***Rhiostomaebenozostera* sp. nov. paratype CUMZ 4704 from Tham Pha Poo, Loei **E***Rhiostomalannaense* sp. nov. paratype CUMZ 4701 from Chiang Dao, Chiang Mai **F***Rhiostomatigrina* sp. nov. paratype CUMZ 4497/2 from Mae Sai, Chiang Rai.

***Holotype***CUMZ 4497/1 (cW 19.5 mm, cH 10.5 mm, dL 20.8 mm; Fig. 31A). ***Paratypes***CUMZ 3909 (29 adults + 10 juveniles), CUMZ 4497/2 (2 shells; Figs 31B, 32F), CUMZ 4806 (13 adults + 5 juveniles; Fig. 31C), CUMZ 10191 (6 adults + 4 juveniles), NHMUK 20220441 (5 shells), and SMF 368676 (5 shells). All paratypes are from the type locality.

##### Type locality.

Tham Pum Tham Pla, Mae Sai District, Chiang Rai Province, Thailand (20°21'00.4"N, 99°51'25.2"E). Limestone hill covered by dry deciduous forest.

##### Other material examined.

**Thailand**: Chiang Dao, North Thailand: ZMUC ex. B. Degerbøl collection (1 alcohol specimen at 400 m, and 2 alcohol specimens at 1100 m). Tham Samta, Muang, Maehongsorn: CUMZ 4441. Wat Pa Tham Wua, Pang Mapha, Maehongsorn: CUMZ 10028. Tham Luang, Khun Nam Nang Non, Mae Sai, Chiang Rai: CUMZ 10186, 10188. Tham Pha Chom, Mae Sai, Chiang Rai: CUMZ 4347. Tham Pla, Mae Sai, Chiang Rai: CUMZ 4346, 4803. Tham Saohin Prayanak, Mae Sai, Chiang Rai: CUMZ 3906, 4442, 10190, 10193. Wat Tham Pla School, Mae Sai, Chiang Rai: CUMZ 3913, 4844, 10189. Tham Mae Suai, Mae Suai, Chiang Rai: CUMZ 3907, 4345, 4807 (Fig. 31E, F). Khao Tham Phra, Muang, Chiang Rai: CUMZ 3915, 4795 (Fig. 31D), 4797, 4839, 4890, 10187. Wat Pha Cha Lui, Pa Dad, Chiang Rai: CUMZ 4435. Wat Tham Phra Bumpenboon, Phan, Chiang Rai: CUMZ 3914, 4495, 4496, 4706, 4846, 10027, 10034. Ban Pang Ma Yao, Chiang Dao, Chiang Mai: CUMZ 10185. Tham Chiang Dao, Chiang Dao, Chiang Mai: CUMZ 3911, 4438, 4499, 4881, 10181, 10182, 10183, 10192. Tham Brichinda, Chom Thong, Chiang Mai: CUMZ 10195. Huai Mae Muk Waterfall, Fang, Chiang Mai: CUMZ 10184.

##### Diagnosis.

Shell small to medium, slightly thin and depressed. Detached whorl medium in length, curved and descending. Breathing device tubular and usually attached to preceding whorl. Shell colour with dark brown zigzag patterns on both sides.

##### Differential diagnosis.

This new species differs from *R.marioni*, *R.jalorensis*, and *R.thachi* in having a brown zigzag colour pattern and short detached whorl. In contrast, these three species have uniformly brownish to dark brown shells (rarely with a blotched pattern) and long and twisted detached whorls. In addition, *R.thachi* has a broader expanded aperture on the palatal side, while *R.trigrina* sp. nov. has a thickened, slightly expanded, multi-layered lip.

##### Description.

***Shell*.** Shell small to medium, cW 15.7–20.5 mm, cH 8.5–11.7 mm, slightly thin, and sub-discoidal to discoidal; detached-whorl length 4.5–13.5 mm. Apex acute and dark; spire convex to nearly flat. Whorls 4 to 5, convex, increasing regularly; suture wide and shallow; last whorl rounded and stout. Shell surface with fine growth lines. Periostracum thin, corneous, and transparent. Shell with brown to dark brown zigzag patterns and faded on ventral shell surface, with narrow, dark, spiral band on periphery. Detached whorl medium in length, approximately the same length as aperture width, curved and descending. Peristome circular and double; lip thickened, expanded and multi-layered. Aperture opened sub-ventrally. Breathing device tubular and curved or straight, and its tip usually attached to preceding whorl; outer lip forming a long and closed tube, and located just behind apertural lip; inner lip with hole inside aperture. Umbilicus widely opened and deep. Operculum calcareous, cup-shaped, and multispiral (Fig. 31).

***Radula*.** Teeth arrangement and shape are similar to those of *R.housei*. Central tooth with triangular central cusp, flanked by two lateral cusps on each side, each pair with pointed tips. Lateral teeth composed of four cusps; central cusp large with dull tip and flanked by one outer and two inner cusps. Marginal teeth each consisting of three pointed cusps (Fig. 32F).

##### Etymology.

The specific name *tigrina* comes from the Latin word *tigris*. It refers to the brownish streak or zigzag colour pattern on the shell surface of this new species, which is similar to the colour pattern of the Bengal tiger, *Pantheratigristigris* (Linnaeus, 1758).

##### Distribution.

This species is mainly distributed in northern Thailand in Chiang Rai, Maehongsorn, and Chiang Mai provinces (Fig. 18).

##### Remarks.

This species has high variation in shell colour, from dark brown zigzag patterns to whitish shells without patterns (Fig. 31F) among specimens from the same collection localities.

### ﻿Group III: *Rhiostomaasiphon* group. Species with very short to nearly absent detached whorl; breathing device a canal or incomplete tube

#### ﻿14. Rhiostoma asiphon

Taxon classificationAnimaliaArchitaenioglossaCyclophoridae

Möllendorff, 1893

07D91D99-A3C2-56DC-A850-9285B6E730CD

**Figure 33.**
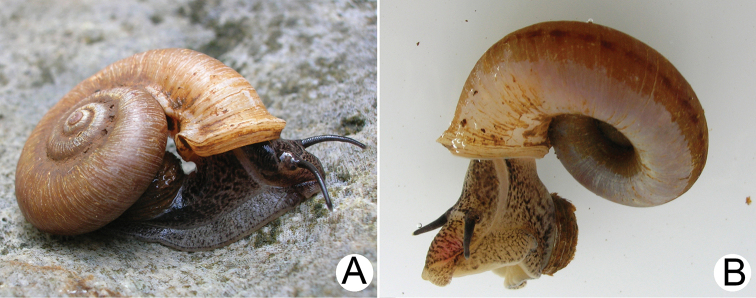
Live snails of the species group III. *Rhiostomaasiphon* specimen CUMZ 4826 from Koh Wua Ta Lab, Ang Angthong, Surat Thani (topotype), **A** life habitus and **B** fully extending body underwater. All figures are not to scale. Photographs: P. Tongkerd

**Figure 34.**
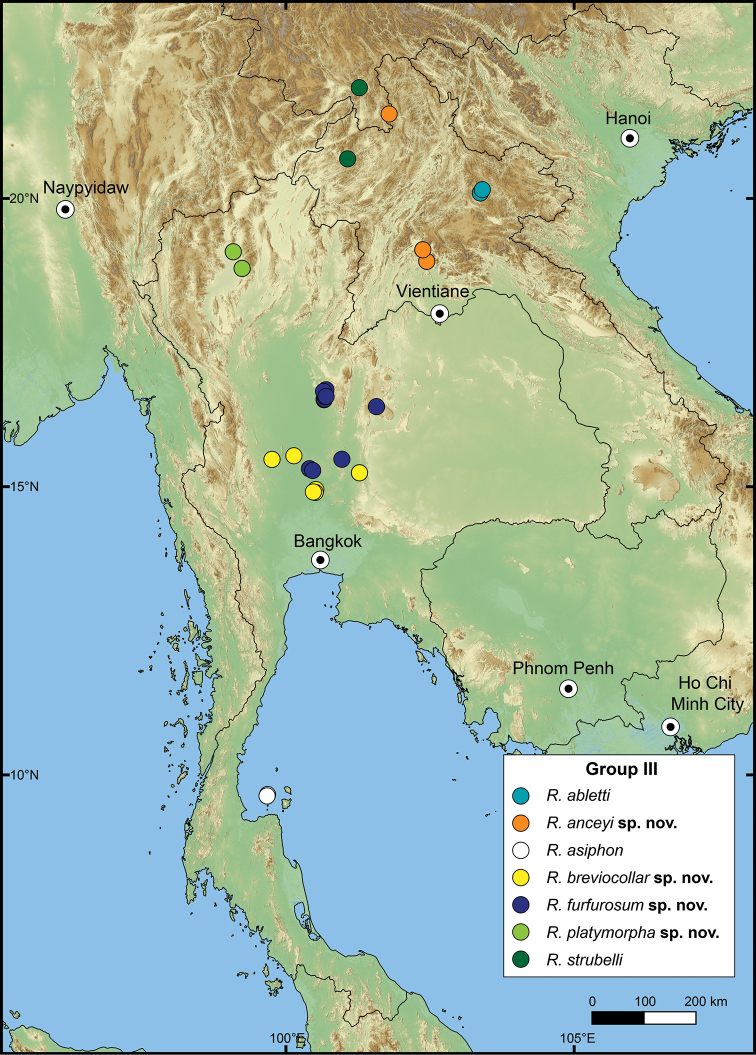
Distribution map of *Rhiostomaasiphon*–species group.

**Figure 35.**
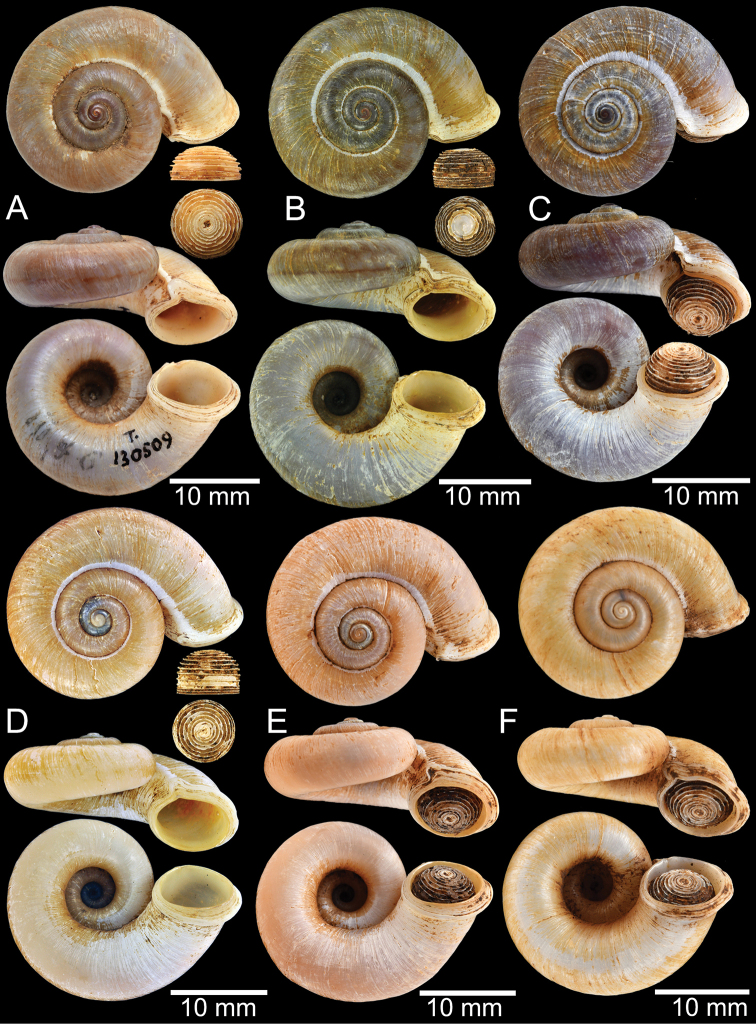
Shell of *Rhiostomaasiphon***A** lectotype SMF 130509 from Insel Samui, Golf von Siam **B, C** specimens CUMZ 4826 from Koh Wua Ta Lab, Angthong Islands, Surat Thani with dark shell form **D–F** specimens CUMZ 4767 from Koh Wua Ta Lab, Angthong Islands, Surat Thani with yellowish shell form.

Figs 33 , 34 , 35 , 42A

Rhiostoma asiphon Möllendorff, 1893: 142. Type locality: Insel Samui, Golf von Siam [Samui Island, Surat Thani Province, Thailand]. Möllendorff 1894: 152, 153, p1. 16, figs 16, 17. Fischer and Dautzenberg 1904: 427. Kobelt 1902: 176. Kobelt 1911: 760, 761, pl. 111, figs 4–8. Zilch 1956: 174. Benthem Jutting 1960: 11. Chan 1996: 5, fig. 3. Kongim et al. 2013b: 16, fig. 2c.
Rhiostoma sp. 7—Tumpeesuwan 2001: 90–92, figs 4.36, 4.37.

##### Type material.

***Lectotype*** (designation in Zilch (1956)) SMF 130509/1 (Fig. 35A) from Golf von Siam: Insel Samui. ***Paralectotypes***SMF 130510/5 (5 shells), SMF 130511/2 (2 shells), SMF 130512/1 (1 shell).

##### Other material examined.

**Thailand**: Siam: NHMW 6657 ex. Gerstenbrandt collection (1 shell). Insel Samui, Golf von Siam: NHMW 40488 ex. Möllendorff collection (1 shell). Koh-Samui, Gulf of Siam: NHMW Rüsnov collection (2 shells). Koh Samsao, Angthong Islands, Koh Samui, Surat Thani: CUMZ 3839, 4756. Koh Wua Ta Lab, Angthong Islands, Koh Samui, Surat Thani: CUMZ 3872, 4365, 4400, 4715, 4730, 4767 (Fig. 35D–F), 4825, 4826 (Figs 33, 35B–C, 42A).

##### Diagnosis.

Shell thick and depressed; detached whorl short. Shell uniformly brown to purplish, sometimes with yellowish colour. Breathing device notch-shaped.

##### Differential diagnosis.

*Rhiostomaaiphon* is superficially similar to *R.samuiense* in having a uniformly brownish to purplish shell and notch-shaped breathing device. However, this species can be distinguished by having an extremely short detached whorl, while *R.samuiense* possesses a medium to long and descending detached whorl.

##### Description.

***Shell*.** Shell small to medium, cW 18.4–22.7 mm, cH 9.4–12.5 mm, thickened, and sub-discoidal shape; detached-whorl length 2.5–8.0 mm. Apex acute, earlier whorls with dark colouration; spire nearly flat. Whorls 4 to 5, convex, increasing regularly; suture wide and very shallow; last whorl rounded and stout. Shell surface with fine growth lines. Periostracum brownish to thin, corneous, and translucent. Shell colour uniformly brown to purplish; with thin and narrow brownish peripheral band sometimes present. Detached whorl shorter than apertural width to nearly absent. Peristome circular and double; lip thickened, expanded, and multi-layered. Aperture opened sub-laterally. Breathing device notch-shaped; outer lip protruding, with shallow notch; inner lip with shallow incision to indistinct. Umbilicus widely opened and deep. Operculum calcareous, cup-shaped, and multispiral (Fig. 35).

***Radula*.** Taenioglossate radula arranged in inverted V-shaped rows. Central tooth with well-developed central cusp and three lateral cusps on each side; central cusp large and tall with blunt tip; lateral cusps triangular, tapering in size and with pointed tips. Lateral teeth consisting of four cusps; central cusp large, blunt tip, and flanked by pointed tips of two inner cusps and one outer cusp. Inner and outer marginal teeth each composed of three cusps; central cusp large with dull tip, flanked by small pointed tips of one inner cusp and one outer cusp (Fig. 42A).

##### Distribution.

The previous record of this species was only from the type locality “Insel Samui, Golf von Siam” (Möllendorff 1893, 1894). The records of “*R.asiphon*” on the Malay Peninsula (Thailand and Malaysia) are based on misidentifications and excluded herein. Therefore, this species is probably endemic to the Ang Thong Islands in Surat Thani Province. These isolated limestone islands are located ~30 km to the west of Samui Island, Surat Thani Province, and this is considered the type locality of this species (Fig. 34).

##### Remarks.

Under the name *Rhiostomaasiphon* [non Möllendorff 1893], this species has been reported from several places on the Malay Peninsula (see Benthem Jutting 1960; Berry 1963; Chan 1996) and also reported as “*Pterocyclus* [sic] *asiphon*” from several localities in southernmost Thailand (Tumpeesuwan 2001). We examined these specimens and revisited all mentioned localities, but found no samples that could be provisionally identified as *R.asiphon* s. s. On the other hand, numerous specimens of *Pterocyclosdiluvium*, common snails in those localities, were collected (see Sutcharit et al. 2014). Their shell morphology is superficially similar to *R.asiphon*. However, the distinguishing characters of the genus *Pterocyclos* are an outer lip expanded at the base, forming a nearly closed tube; an apertural groove is located inside the aperture near the apertural lip; and a calcified plate-shaped operculum is present (Fig. 2). Therefore, we conclude that the nominal species name *R.asiphon* in Benthem Jutting (1960), Berry (1963), and Chan (1996) and *Pterocyclusasiphon* in Tumpeesuwan (2001) and Tumpeesuwan and Panha (2003) are erroneously identified and should be recognised as *Pterocyclosdiluvium*.

Of the paralectotypes SMF 130510/5 (5 shells), four of them are identical to the lectotype specimen (Fig. 35A). The exception is a single specimen exhibiting canal-shaped breathing device that is expanded at the base near the suture, similar to the distinct characters of either *R.morleti* or *R.prestoni*. However, this is beyond the range of individual variation, and the recent topotype specimens (~100 shells) included no shells exhibiting these peculiar characters. Therefore, we excluded that specimen from our attention in the type series.

#### ﻿15. Rhiostoma strubelli

Taxon classificationAnimaliaArchitaenioglossaCyclophoridae

Möllendorff, 1899

09AEBD73-0773-5DBE-AA2D-351792EEC068

Figs 34 , 36 , 41B

Rhiostoms strubelli Möllendorff, 1899: 166. Type locality: Kalow, 5000’, südliche Shan Staaten [Kalaw, Taunggyi District, Shan State, Myanmar]. Kobelt 1902: 179. Kobelt 1911: 763, pl. 113, fig. 11. Zilch 1956: 174.

##### Type material.

**Figure 36.**
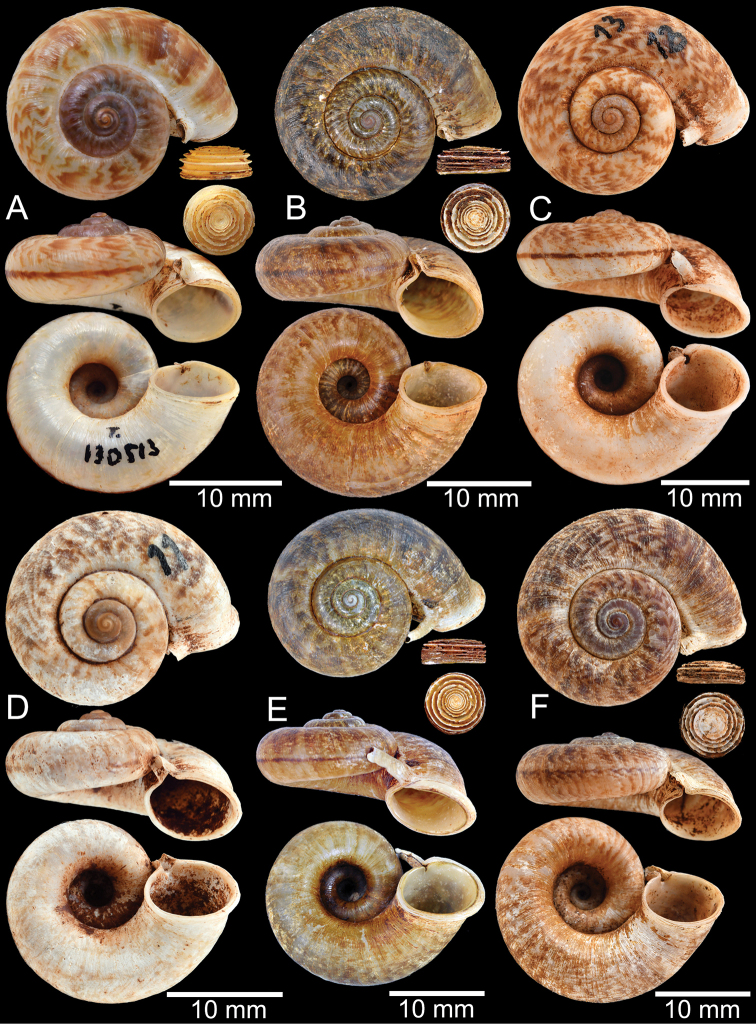
Shell of *Rhiostomastrubelli***A** lectotype SMF 130513 from S-Shan Staaten: Kalow **B–E** specimens CUMZ 10200 from Vieng Phuka, Luang Namtha, Laos and **E** with long breathing device **F** specimen CUMZ 10047 from Xishuangbanna Botanical Garden, Yunnan, China.

The original description did not explicitly indicate the number of specimens, and was without illustration or explicit designation of the name-bearing type, although one set of measurements was given in Möllendorff (1899). Later, Kobelt (1911: pl. 113, fig. 11) illustrated this species for the first time, and then Zilch (1956) recognised this figured specimen (SMF 130513) as the “holotypus”. This SMF collection lot contains only one specimen; therefore, the subsequent use of the term “holotypus” in Zilch (1956) constitutes a valid lectotype designation. ***Lectotype*** (designation in Zilch (1956)) SMF 130513 (Fig. 36A) from S-Shan Staaten: Kalow, 5000’.

##### Other material examined.

**China**: Xishuangbanna Tropical Botanical Garden, Mengla County, Xishuangbanna Prefecture, Yunnan Province: CUMZ 10047 (7 shells; Fig. 36F). **Laos**: Khao Pa Huak, Vieng Phuka District, Luang Nam Tha Province: CUMZ 10200 (29 shells; Figs 36B–E, 42B).

##### Diagnosis.

Shell small, thin, and depressed; detached whorl shorter than apertural width. Shell with brownish zigzag patterns. Breathing device short and incomplete tube shape.

##### Differential diagnosis.

*Rhiostomastrubelli* can be distinguished from *R.haughtoni* and *R.tigrina* sp. nov. by having a smaller shell, very short detached whorl, incomplete tubular shape of breathing device, and apex with dark colouration. In contrast, *R.haughtoni* has a canal-shaped breathing device with a monochrome shell, while *R.tigrina* sp. nov. has a long, detached whorl and short to long breathing device with complete tube shape.

##### Description.

***Shell*.** Shell small, cW 17.3–22.9 mm, cH 8.5–12.0 mm, thin, and sub-discoidal shell; detached-whorl length 2.0–7.0 mm. Apex acute and dark colouration; spire slightly elevated. Whorls 4 to 5, convex, increasing regularly; suture wide and shallow; last whorl rounded. Shell surface with fine growth lines. Periostracum thin, corneous, and transparent. Shell colour with brown zigzag pattern, and narrow dark brown spiral band on periphery. Detached whorl shorter than apertural width. Peristome circular and double; lip thickened and slightly expanded. Aperture opened sub-laterally. Breathing device with incomplete tube shape; outer lip protruded into a nearly closed tube; inner lip with deep incision or small hole inside aperture. Umbilicus widely opened and deep. Operculum calcareous, low cup-shaped, and multispiral (Fig. 36).

***Radula*.** Teeth arrangement and shape are similar to *R.asiphon*. Central tooth with large central cusp and two lateral cusps on each side. Lateral teeth having four cusps; central cusp large, triangular, and flanked by two small inner cusps and one outer cusp. Marginal teeth each consisting of three pointed cusps (Fig. 42B).

##### Distribution.

This species is known from Kalaw, Shan State, Myanmar. This study recorded specimens from Luang Namtha Province, Laos, and Yunnan in China (Fig. 34).

##### Remarks.

This species is previously known only from the holotype from the south of Shan State, Myanmar, which is close to northern Thailand and Laos. However, no *Rhiostoma* specimens collected from northern Thailand could be identified as this species. The specimen from Luang Namtha Province, Laos and Yunnan Province, China are very similar to the lectotype in having short detached whorl and breathing device with an incomplete tube shape. The only difference is in having darker irregular blotches on the shell than the type specimen, and this is considered as intrapopulation variation.

#### ﻿16. Rhiostoma abletti

Taxon classificationAnimaliaArchitaenioglossaCyclophoridae

Thach, 2016

37F4588D-4F34-5A08-AE01-AC391B2D01C7

Figs 34 , 37

Rhiostoma abletti Thach, 2016: 37, 38, figs 53, 122–124. Type locality: Northwest of Lai Chau city, on the way going to Paso, Lai Chau Province, North Vietnam. Sutcharit et al. 2019: 5, fig. 1a.
Rhiostoma christae Thach, 2016: 38, figs 51, 130–133. Type locality: Near the road No. 6 to Chieng Ngan, Son La Province, North Vietnam. Sutcharit et al. 2019: 17, fig. 3l. New synonym.
Rhiostoma ninhbien Do, Nguyen & Do, 2020b: 169, 170, fig. 2a–c. Type locality: near Tay Trang international border gate, Na U Commune, Dien Bien District, Dien Bien Province, Vietnam. New synonym.
Rhiostoma marioni —Do et al. 2020b: 168 (in part), fig. 1e, f (not Ancey 1898).

##### Type material.

**Figure 37.**
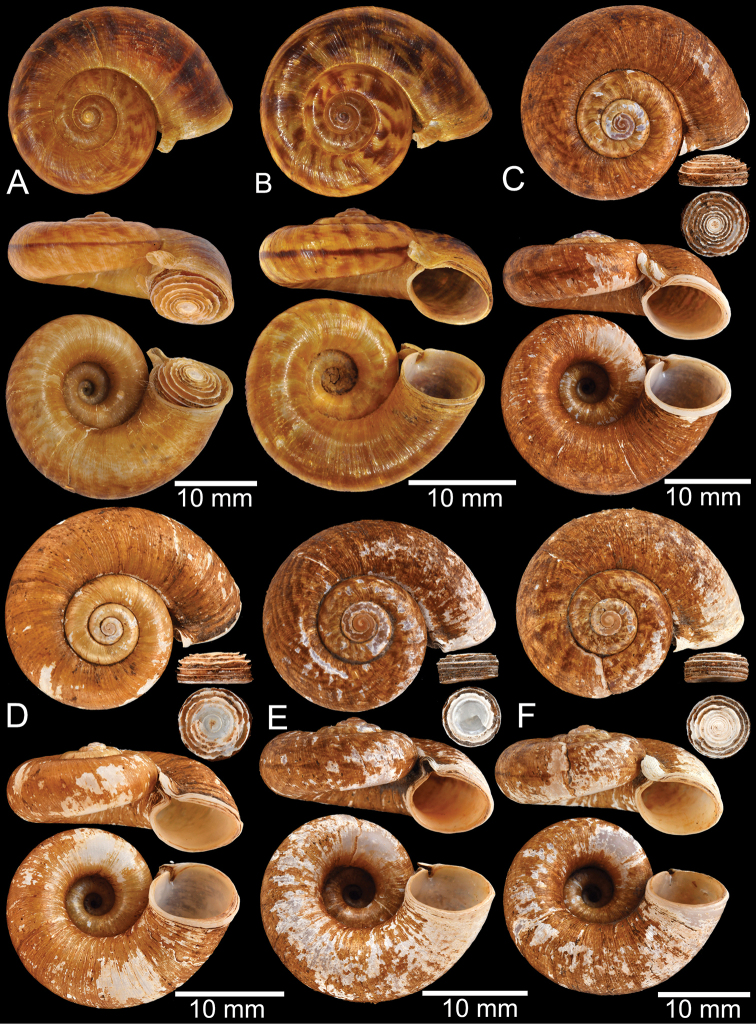
Shell of *Rhiostomaabletti***A** holotype NHMUK 20160307 from Lai Chau Province, Vietnam **B** holotype of *Rhiostomachristae* Thach, 2016, NHMUK 20160306 from Son La Province, Vietnam **C, D** specimens CUMZ 10207 from Ban Na Puek, Meuang Hiam, Houaphanh, Laos **E, F** specimens CUMZ 10206 from Hot Springs, Meuang Hiam, Houaphanh, Laos.

***Holotype***NHMUK 20160307 (Fig. 37A) from Northwest of Lai Chau city, on the way going to Paso, Lai Chau Province, Vietnam. ***Holotype***NHMUK 20160306 (Fig. 37B) of *Rhiostomachristae* Thach, 2016, from Near the road No. 6 to Chieng Ngan, Son La Province (North Vietnam).

##### Other material examined.

**Laos**: Hot Springs (Vieng Thong), Meuang Hiam District, Houaphanh Province: CUMZ 10206 (3 shells; Fig. 37E, F). Near Ban Na Puek, Meuang Hiam District, Houaphanh Province: CUMZ 10207 (2 shells; Fig. 37C, D).

##### Diagnosis.

Shell medium, thin and flattened; detached whorl shorter than apertural width. Shell colour with brownish zigzag patterns. Breathing device with incomplete tube shape and nearly perpendicular to detached whorl.

##### Differential diagnosis.

This species can be distinguished from *R.anceyi* sp. nov. by having an incomplete tube breathing device that is nearly perpendicular to detached whorl, and translucent periostracum. In contrast, the new species has a tubular breathing device, and thickened and reddish brown periostracum.

##### Description.

***Shell*.** Shell medium, width 38.5 mm, height 14.5 mm, thin, and flattened to sub-discoidal shape. Apex acute; spire slightly elevated to flattened. Whorls 5 to 6, convex, increasing regularly; suture wide and depressed; last whorl rounded and stout. Shell surface with fine growth lines. Periostracum thick corneous and translucent. Shell colour with dark brown zigzag pattern; with narrow dark spiral band on periphery. Detached whorl shorter than apertural width. Peristome circular and double; lip thickened and slightly expanded. Aperture opened sub-laterally. Breathing device with incomplete tube shape and nearly perpendicular to detached whorl; outer lip forming a short to long nearly closed tube; inner lip with deep incision or small hole inside aperture. Umbilicus widely opened and deep. Operculum calcareous, low cup-shaped, and multispiral (Fig. 37).

##### Distribution.

This species is known from Lai Chau, Son La and Dien Bien provinces, northern Vietnam. The current study recorded specimens from a few localities in Hua Phan Province, northern Laos (Fig. 34).

##### Remarks.

The holotypes of *R.abletti* from Lai Chau Province (Fig. 37A), “*R.christae*” from Son La Province (Fig. 37B) and “*R.ninhbien*” from Dien Bien Province (see Do et al. 2020b: fig. 2a–c) are nearly identical in appearance. They have a depressed shell, with short detached whorl and incomplete tube shape, but are slightly different in the shell colour pattern, which could not be used as a diagnostic trait. In addition, the type localities of these three species are in neighbouring areas of northern Vietnam, less than 150 km apart. Therefore, we recognise *R.christae* and *R.ninhbien* as junior subjective synonyms of *R.abletti*.

The specimens from Hua Phan Province, Laos, tend to have thicker periostracum and more coarse growth lines than the type specimen. However, a short detached whorl and incomplete tube as breathing device suggest they are more closely related to *R.abletti*.

#### ﻿17. Rhiostoma anceyi

Taxon classificationAnimaliaArchitaenioglossaCyclophoridae

Tongkerd & Inkhavilay sp. nov.

68E5935D-B3D3-5B30-9D84-DB6668218CD7

https://zoobank.org/5A3D8DA2-5C54-45ED-8FD9-4220FBD8E2E3

Figs 34 , 38 , 42C

##### Type material.

**Figure 38.**
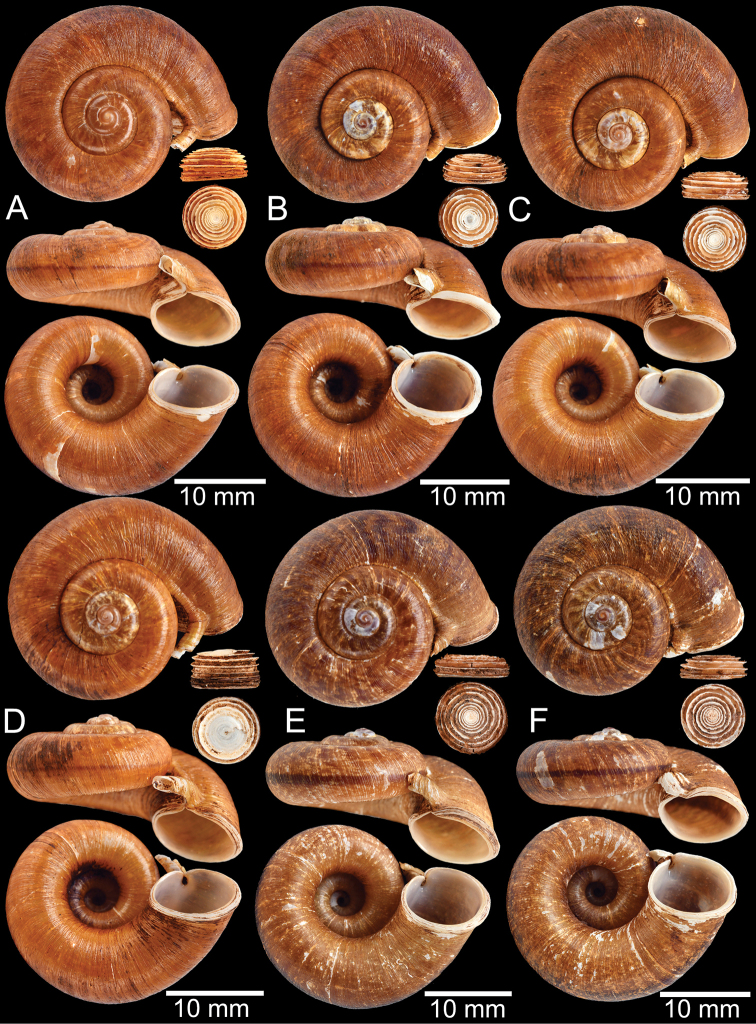
Shell of *Rhiostomaanceyi* sp. nov. **A** holotype CUMZ 4494/1 from Pha Hom, Phoxay, Vientiane, Laos **B, C** paratypes CUMZ 4494/2 from type locality **D** paratype CUMZ 4887 from type locality **E, F** specimens CUMZ 10208 from Ban Bokhoun, Boun Neua, Phongsaly, Laos.

***Holotype***CUMZ 4494/1 (cW 22.7 mm, cH 11.4 mm, dL 9.7 mm; Fig. 38A). ***Paratypes***CUMZ 4374 (9 shells), CUMZ 4465 (4 adults + 1 juvenile), CUMZ 4494/2 (4 shells; Fig. 38B, C), CUMZ 4758 (5 adults + 1 juvenile), CUMZ 4887 (3 shells; Fig. 38D), CUMZ 10044 (26 adults + 19 juveniles), NHMUK 20220442 (5 shells), and SMF 368677 (5 shells). All paratypes are from the type locality.

##### Type locality.

Massive limestone karsts in Ban Pha Hom (village), Phoxay District, Vientiane Province, Laos (19°06'27.9"N, 102°22'47.2"E).

##### Other material examined.

**Laos**: Near Ban Bokhoun, Boun Neua District, Phongsaly Province: CUMZ 10208 (3 shells; Fig. 38E, F).

##### Diagnosis.

Shell medium to large, thin, and depressed; detached whorl shorter than apertural width. Shell colour uniformly brownish, sometimes with dark zigzag patterns. Periostracum thick and reddish brown. Breathing device with short tubular shape.

##### Differential diagnosis.

*Rhiostomanceyi* sp. nov. differs from *R.furfurosum* sp. nov. by having a tubular breathing device, thickened brownish periostracum, and thickened multi-layered apertural lip. In contrast, *R.furfurosum* sp. nov. has an incomplete tube breathing device, transparent corneous periostracum, and a thickened, widely expanded apertural lip.

##### Description.

***Shell*.** Shell medium, cW 19.0–23.5 mm, cH 10.4–13.7 mm, slightly thin, and sub-discoidal shape; detached-whorl length 4–7.5 mm. Apex acute; spire slightly elevated. Whorls 5 to 6, convex, increasing regularly; suture wide and deep; last whorl rounded and stout. Shell surface with fine growth lines. Periostracum thick, corneous, and reddish brown. Shell colour uniformly brownish or with dark brown zigzag pattern; with dark spiral band on periphery. Detached whorl shorter than apertural width and slightly descending. Peristome circular and double; lip thickened and slightly expanded. Aperture opened sub-laterally. Breathing device tubular (sometimes with incomplete tube) and perpendicular to detached whorl; outer lip forming a short and closed tube; inner lip with small hole inside aperture. Umbilicus widely opened and deep. Operculum calcareous, low cup-shaped, and multispiral (Fig. 38).

***Radula*.** Teeth shapes are similar to those of *R.asiphon*; only minor variations occur in central tooth, with triangular and dull central cusp. Lateral teeth composed of four cusps, innermost lateral cusp small (Fig. 42C).

##### Etymology.

The species name *anceyi* is in honour of César-Marie-Felix Ancey (1860–1906), one of the pioneer malacologists who studied the land snail materials from Laos (Wood and Gallichan 2008).

##### Distribution.

This new species is known only from Vientiane and Phongsaly provinces, Laos (Fig. 34).

##### Remarks.

More effort in molecular phylogenetic analysis is required to demonstrate the relationship between this and the other congeners.

#### ﻿18. Rhiostoma breviocollar

Taxon classificationAnimaliaArchitaenioglossaCyclophoridae

Tongkerd & Tumpeesuwan sp. nov.

00ED7F36-5A49-51D7-8B1C-F5D3A44DAB81

https://zoobank.org/A0126A3A-DD78-4E6A-8C92-81D086588C57

Figs 34 , 39 , 42D

Rhiostoma sp. 4—Tumpeesuwan 2001: 76–81, figs 4.28–4.30 (in part).
Rhiostoma sp. 8—Tumpeesuwan 2001: 93–95, figs 4.38, 4.39.

##### Type material.

**Figure 39.**
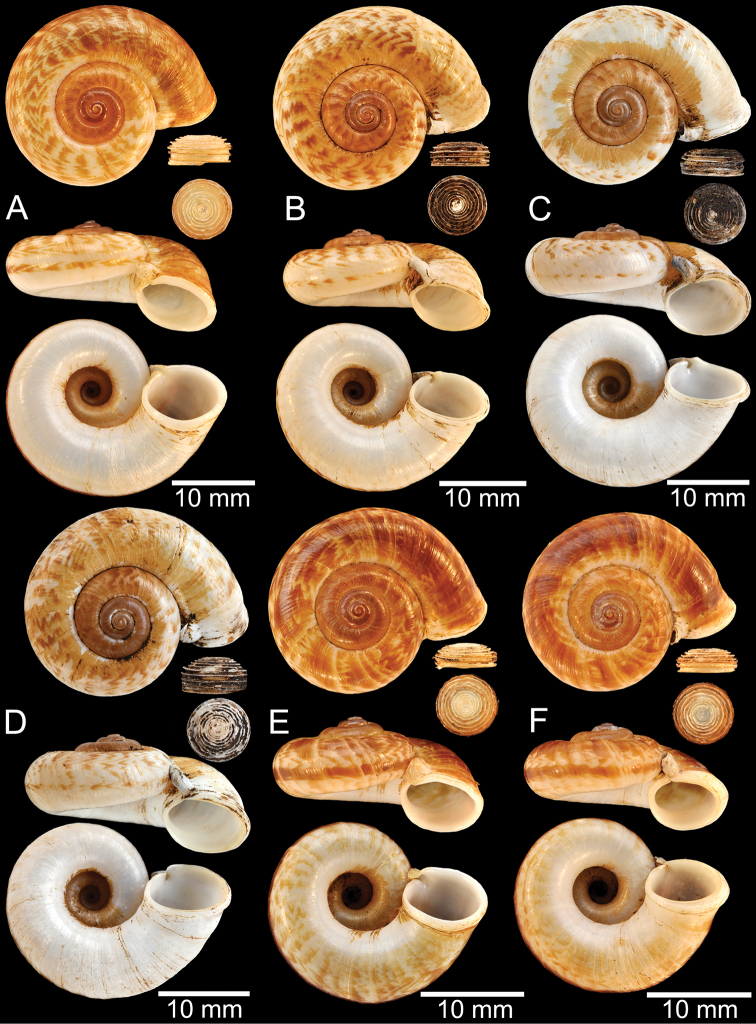
Shell of *Rhiostomabreviocollar* sp. nov. **A** holotype CUMZ 4490 from Khao Samorkon, Ban Mi, Lopburi **B–D** paratypes CUMZ 4491 from type locality **E, F** specimens from Khao Patavi, Tubtan, Uthai Thani **E** specimen CUMZ 4492 and **F** specimens CUMZ 4493.

***Holotype***CUMZ 4490 (cW 22.8 mm, cH 11.2 mm, dL 8.2 mm; Fig. 39A). ***Paratypes***CUMZ 3975 (139 adults + 10 juveniles), CUMZ 4366 (21 shells), CUMZ 4491 (6 shells; Figs 39B–D, 42D), CUMZ 10159 (4 adults + 4 juveniles), NHMUK 20220443 (5 shells), and SMF 368678 (5 shells). All paratypes are from the type locality.

##### Type locality.

Small isolated limestone hill at Wat Khao Smokon, Ban Mi District, Lopburi Province, Thailand (14°54'25.9"N, 100°30'21.9"E).

##### Other material examined.

**Thailand**: Khao Noi, Muang, Nakhonsawan: CUMZ 4352. Khao Patavi, Tubtan, Uthai Thani: CUMZ 3804, 3900, 3978, 4358, 4492 (Fig. 39E), 4493 (Fig. 39F), 4772. Khao Tee Hin, Ban Mi, Lopburi: CUMZ 4367. Wat Tham Tambon, Chai Ba Dan, Lopburi: CUMZ 3888, 3983, 4445, 4466, 4788, 4841, 10019, 10157.

##### Diagnosis.

Shell small to medium, thick, and depressed; detached whorl shorter than apertural width. Shell colour whitish and with brownish zigzag patterns. Breathing device incomplete tube shape.

##### Differential diagnosis.

*Rhiostomabreviocollar* sp. nov. can be distinguished from *R.anceyi* sp. nov. by having a thin corneous periostracum and incomplete tube as a breathing device. In contrast, *R.anceyi* sp. nov. has a thickened and brownish periostracum and a short tubular breathing device.

The COI barcoding suggests a close relationship between this new species and *R.housei* (Fig. 3). However, this new species differs by having flattened to a sub-discoidal shell, short to lacking detached whorl and incomplete tube as breathing device. In contrast, *R.housei* has a discoidal shell, long detached whorl, and a tubular breathing device.

##### Description.

***Shell*.** Shell small to medium, cW 21.6–25.5 mm, cH 10.0–12.8 mm, thick, and flattened to sub-discoidal shape; detached-whorl length 3.0–7.5 mm. Apex acute; spire nearly flattened to slightly elevated. Whorls 4 to 5, convex, increasing regularly; suture wide and shallow; last whorl rounded and stout. Shell surface with irregular growth lines. Shell colour with brownish zigzag or blotch patterns; ventral side with paler colour patterns; narrow dark brown spiral band on periphery. Periostracum thick or thin, corneous, and transparent. Detached whorl very short (sometimes nearly absent) and slightly descending. Peristome circular and double; lip thickened, expanded, and multi-layered. Aperture opened sub-laterally. Breathing device incomplete tube-shaped and its tip sometimes attached to preceding whorl; outer lip forming a short and nearly closed tube; inner lip with deep incision. Operculum calcareous, cup-shaped, and multispiral (Fig. 39).

***Radula*.** Teeth shapes are similar to those of *R.asiphon*; only minor variations occur in the central tooth with low triangular-shaped central cusp. Lateral teeth composed of three or four cusps, innermost lateral cusp very small to nearly absent (Fig. 42D).

##### Etymology.

The species name *breviocollar* comes from the two Latin words *brevis* meaning short or shorter, and *collum* meaning neck. Thus, it refers to a very short to absent detached whorl, a distinguishing character of this species.

##### Distribution.

This nominotypical species is recorded from several localities in Lopburi and Uthai Thani provinces, central Thailand (Fig. 34).

##### Remarks.

*Rhiostomahousei* and *R.furfurosum* sp. nov. have overlapping distribution ranges with *R.breviocollar* sp. nov., while the COI barcoding indicates they are distinct species (Fig. 3). However, additional morphometric analyses will be needed to identify differences in shell form.

#### ﻿19. Rhiostoma furfurosum

Taxon classificationAnimaliaArchitaenioglossaCyclophoridae

Tongkerd & Panha sp. nov.

9DD703EE-7A8F-5004-9DF8-8B2B81DE935F

https://zoobank.org/FC154E96-C52E-4272-8AFA-ADB73D77BCDE

Figs 34 , 40 , 42E

Rhiostoma sp. 3—Tumpeesuwan 2001: 71–75, figs 4.25–4.27.
Rhiostoma sp. 4—Tumpeesuwan 2001: 76–81, figs 4.28–4.30 (in part).

##### Type material.

**Figure 40.**
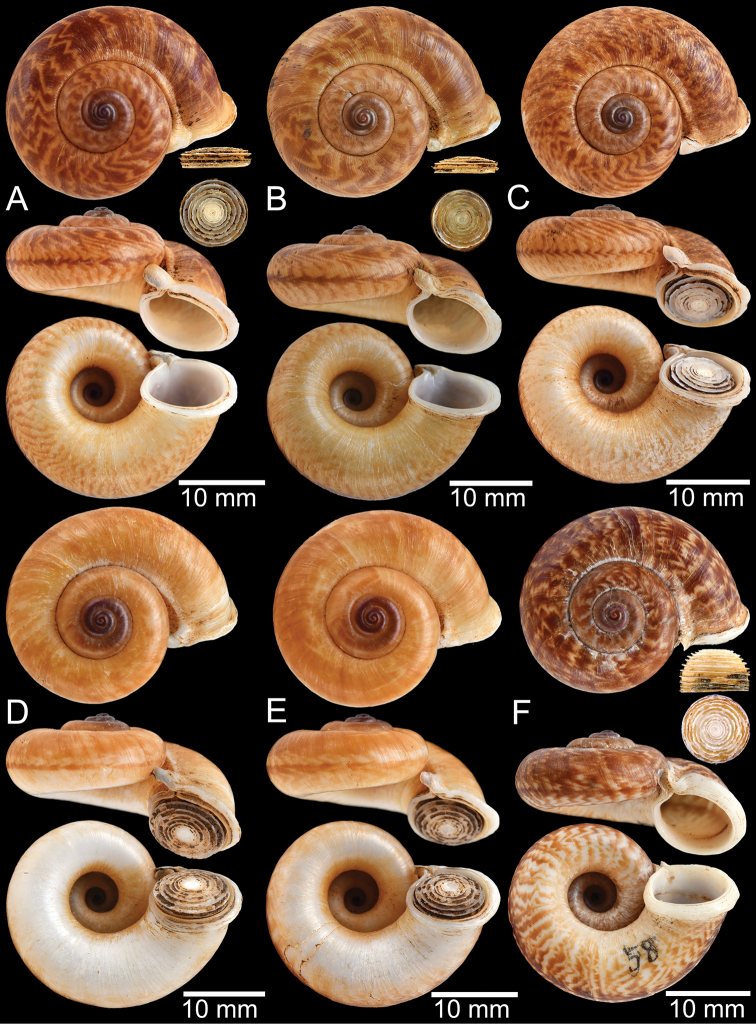
Shell of *Rhiostomafurfurosum* sp. nov. **A** holotype CUMZ 4705/1 from Tham Wang Dang, Noern Maprang, Phitsanulok **B, C** paratypes CUMZ 4705/2 from type locality **D, E** specimens CUMZ 3980 from Pa Ma Muang, Noern Maprang, Phitsanulok **F** specimen CUMZ 4353 from Phu Khiao, Chaiyaphum.

***Holotype***CUMZ 4705/1 (cW 23.2 mm, cH 13.1 mm, dL 9.5 mm; Fig. 40A). ***Paratypes***CUMZ 3901 (37 adults + 3 juveniles), CUMZ 3903 (105 adults + 11 juveniles), CUMZ 3904 (105 adults + 8 juveniles), CUMZ 3905 (200 adults + 14 juveniles), CUMZ 4447 (81 adults + 2 juveniles), CUMZ 4705/2 (6 shells; Figs 40B, C, 42E), CUMZ 4833 (51 adults + 4 juveniles), CUMZ 4835 (5 shells), CUMZ 4884/1 (70 adults + 2 juveniles), CUMZ 10151 (1 shell), CUMZ 10152 (8 shells), NHMUK 20220444 (5 shells), and SMF 368679 (5 shells). All paratypes are from the type locality.

##### Type locality.

Tham Wang Dang, Noern Maprang District, Phitsanulok (16°41'40.1"N, 100°40'42.5"E). Limestone hill with dry dipterocarp forest and surrounded by paddy fields.

##### Other material examined.

**Thailand**: Pa Ma Muang Bureau of Monks, Noern Maprang, Phitsanulok: CUMZ 3980 (Fig. 40D, E), 4446, 4885, 10023, 10148, 10149, 10150, Tham Bot Wang Na, Noern Maprang, Phitsanulok: CUMZ 10153. Tham Pha Tha Phol, Noern Maprang, Phitsanulok: CUMZ 4359, 4869, 10024. Tham Tao, Noern Maprang, Phitsanulok: CUMZ 3902. Tham Wang Thong, Noern Maprang, Phitsanulok: CUMZ 10147. Wat Ban Mung, Noern Maprang, Phitsanulok: CUMZ 10154. Wat Tham Muang, Noern Maprang, Phitsanulok: CUMZ 4356. Wat Tham Rue-si Mongkon, Srithep, Phetchabun: CUMZ 10016. Phu Keiw Wildlife Sanctuary, Phu Khiao, Chaiyaphum: CUMZ 4314, 4317, 4353 (Fig. 40F). Tham Saeng Wiset Monastic Residence, Takfa, Nakhonsawan: CUMZ 10018, 10030. Wat Tham Khao Chaithong, Takfa, Nakhonsawan: CUMZ 10161.

##### Diagnosis.

Shell medium, thick, and depressed; detached whorl shorter than apertural width. Shell colour brownish with dark brown zigzag patterns. Breathing device incomplete tube-shaped, and usually perpendicular to detached whorl.

##### Differential diagnosis.

*Rhiostomafurfurosum* sp. nov. differs from *R.breviocollar* sp. nov. by having a relatively larger shell size, and thickened and expanded apertural lip, whereas *R.breviocollar* sp. nov. has relatively smaller shell size, and apertural lip thickened and multi-layered.

##### Description.

***Shell*.** Shell medium, cW 21.7–24.7 mm, cH 11.1–13.9 mm, thickened, and sub-discoidal to discoidal shape; detached-whorl length 5.0–9.5 mm. Apex acute with dark colouration; spire elevated. Whorls 5 to 6, convex, increasing regularly; suture wide and depressed; last whorl rounded and slender. Shell surface with fine growth lines. Shell colour with brownish to reddish brown zigzag pattern and ventral surface with paler colour pattern or sometimes with uniformly brownish colour; narrow dark spiral band on periphery. Periostracum thick corneous and brown. Detached whorl shorter than apertural width. Peristome circular and double; lip thickened, expanded, and multi-layered. Breathing device short to long with incomplete tube shape and usually perpendicular to detached whorl; outer lip forming a short to long and nearly closed tube; inner lip with deep incision. Operculum calcareous, low cup-shaped, and multispiral (Fig. 40).

***Radula*.** Teeth arrangement and shape are similar to those of *R.asiphon*. Central tooth with large central cusp and two lateral cusps on each side tapered in size. Lateral teeth consisting of three cusps; inner cusp relatively small with pointed tip; outer cusp large with dull tip. Marginal teeth each consisting of three pointed cusps (Fig. 42E).

##### Etymology.

The species name *furfurosum* comes from the Latin, meaning like bran or brownish. Thus, it refers to the prominent brownish shell colour of this new species.

##### Distribution.

This new species was found only in small, isolated limestone hills in Phitsanulok, Chaiyaphum, and Phetchabun provinces (Fig. 34).

##### Remarks.

Shell variation can be observed by the shell colour ranging from monochrome reddish brown to brown zigzag patterns. Furthermore, the shape of the breathing device varies from incomplete tubular to canal-shaped. In addition, the specimens from Phu Kiew, Chaiyaphum (Fig. 40F) exhibit a lack of detached whorl, canal-shaped breathing device (rarely with short incomplete tube breathing device) attached to the preceding whorl, and with a dark brown zigzag pattern on both sides of the shell. However, we recognise this as variation resulting from an isolated population, while the similarities to *R.furfurosum* sp. nov. suggest that is the same species. Their shell morphologies are clearly distinct from other known species recorded in the adjacent areas.

#### ﻿20. Rhiostoma platymorpha

Taxon classificationAnimaliaArchitaenioglossaCyclophoridae

Tongkerd & Tumpeesuwan sp. nov.

D1130B98-97CF-5F86-8B09-6212955B437C

https://zoobank.org/13B875AD-3E60-431E-B4B2-51F3FC5E03A0

Figs 34 , 41 , 42F

Rhiostoma sp. 1—Tumpeesuwan 2001: 59–61, figs 4.19–4.21 (in part).

##### Type material.

**Figure 41.**
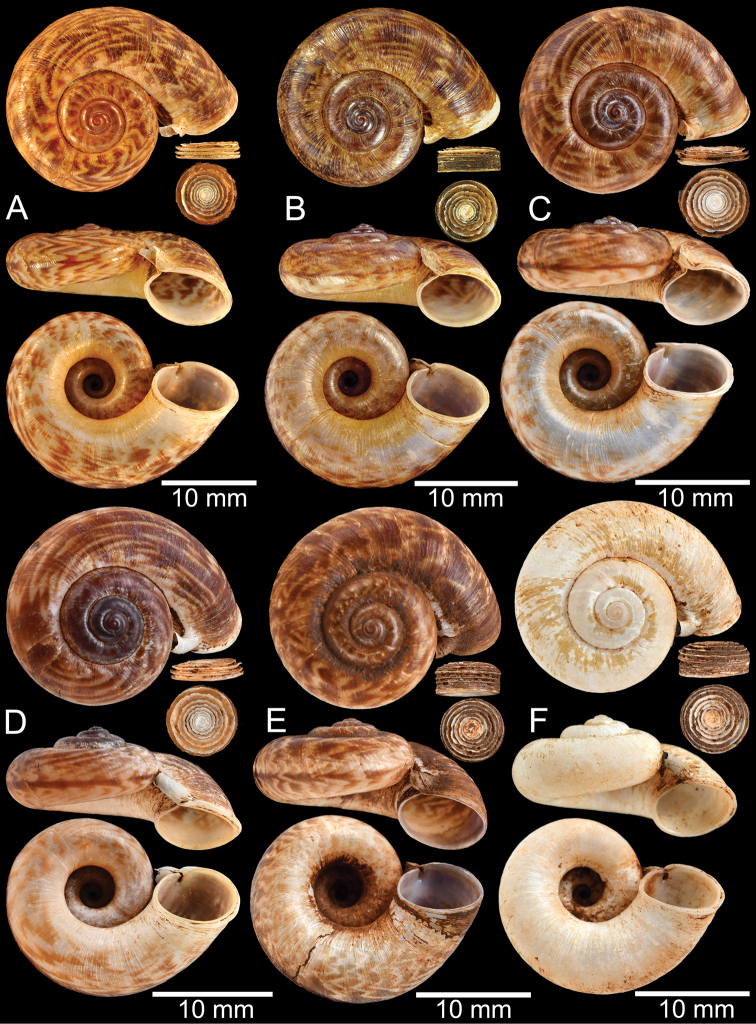
Shell of *Rhiostomaplatymorpha* sp. nov. **A** holotype CUMZ 4498/1 from Tham Muang On, Mae On, Chiang Mai **B** paratype CUMZ 4763 from type locality **C, D** paratypes CUMZ 4498/2 from type locality **E, F** specimens CUMZ 4783 from Tham Bua Tong, Mae Tang, Chiang Mai (with **F** albinistic shell form).

**Figure 42.**
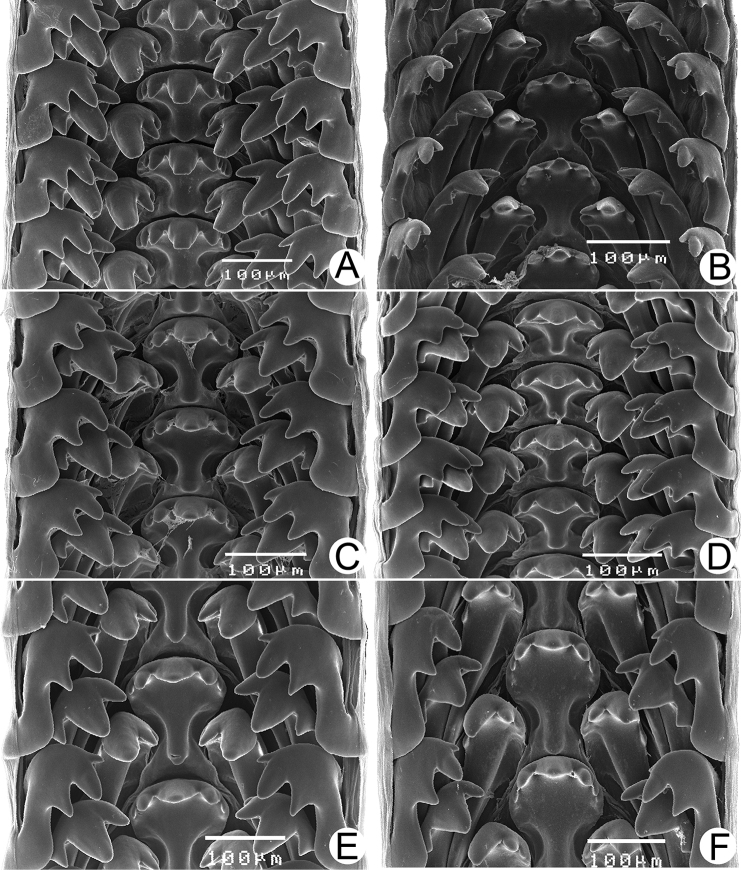
Radula morphology **A***Rhiostomaasiphon*CUMZ 4826 from Angthong Islands, Surat Thani **B***Rhiostomastrubelli* specimen CUMZ 10200 from Vieng Phuka, Luang Namtha, Laos **C***Rhiostomabreviocollar* sp. nov. paratypes CUMZ 4491 from type locality **D***Rhiostomaanceyi* sp. nov. paratype CUMZ 4494/2 from type locality **E***Rhiostomafurfurosum* sp. nov. paratype CUMZ 4705/2 from type locality **F***Rhiostomaplatymorpha* sp. nov. paratype CUMZ 4763 from type locality.

***Holotype***CUMZ 4498/1 (cW 19.0 mm, cH 8.4 mm, dL 7.2 mm; Fig. 41A). ***Paratypes***CUMZ 3976 (22 shells), CUMZ 4349 (1 shell), CUMZ 4436 (4 adults + 1 juvenile), CUMZ 4498/2 (6 shells; Fig. 41C, D), CUMZ 4731 (1 adult + 1 juvenile), CUMZ 4763 (4 shells; Figs 41B, 42F), CUMZ 4801 (48 shells), CUMZ 4809 (74 shells), CUMZ 4892 (10 adults + 1 juveniles), CUMZ 10196 (3 shells), CUMZ 10198 (12 adults + 2 juveniles), CUMZ 10199 (1 shell), NHMUK 20220445 (5 shells), and SMF 368680 (5 shells). All paratypes are from the type locality.

##### Type locality.

Tham Muang On, Mae On District, Chiang Mai Province, Thailand (18°47'10.6"N, 99°14'17.1"E). Limestone hill with dry evergreen forest. The locality is surrounded by a timber plantation and farms for annual crops.

##### Other material examined.

**Thailand**: Tham Boa Tong, Mae Tang, Chiang Mai: CUMZ 4437, 4783 (Fig. 41E, F), 10197.

##### Diagnosis.

Shell small, thin, and flattened; detached whorl shorter than apertural width. Shell colour with brownish zigzag patterns on both sides. Breathing device with incomplete tube shape (rarely short tubular) and attached to preceding whorl.

##### Differential diagnosis.

This new species can be distinguished from *R.strubelli* by having a flattened shell, short detached whorl, and brownish zigzag colour pattern, usually on both the dorsal and ventral sides of the shell. In contrast, *R.strubelli* has a sub-discoidal to discoidal shell shape, and brownish zigzag pattern only on the dorsal side.

##### Description.

***Shell*.** Shell small, cW 17.2–20.2 mm, cH 8.8–11.3 mm, thin, and flattened to sub-discoidal shape; detached-whorl length 2.0–6.0 mm. Apex acute; spire nearly flattened to slightly elevated. Whorls 4 to 5, convex, increasing regularly; suture wide and shallow; last whorl rounded and slender. Shell surface with irregular growth lines. Periostracum corneous and transparent. Shell colour with dark to brownish zigzag pattern, and ventral side with paler pattern; narrow dark brown spiral band on periphery. Detached whorl shorter than apertural width and descending. Peristome circular and double; lip slightly thickened and not expanded. Breathing device with incomplete tube shape and its tip usually attached to preceding whorl; outer lip forming a long and nearly closed tube; inner lip with deep incision. Umbilicus widely opened and deep. Operculum calcareous, low cup-shaped, and multispiral (Fig. 41).

***Radula*.** Teeth arrangement and morphology are similar to those of *R.asiphon*. Central tooth with large central cusp and two lateral cusps on each side. Lateral teeth composed of four cusps; central cusp large with pointed tip; two inner cusps with pointed tips; one outer cusp very small. Marginal teeth each composed of three pointed cusps (Fig. 42F).

##### Etymology.

The subspecific name *platymorpha* comes from two Latin words *platy*, which means flat, and *morpha* meaning form. It refers to the prominent flattened shell shape of this species.

##### Distribution.

This new species is known from the type locality, limestone hills in Chiang Mai Province.

##### Remarks.

The specimens from Tham Buatong, Chaing Mai (Fig. 41E, F), tend to differ from the type specimen in having sub-discoidal shells. However, the brownish zigzag colour pattern appears on the dorsal and ventral shell surface, and an incomplete tube-shaped breathing device suggests they are closely related to this species. That being noted, a few specimens with monochrome whitish shells also occur in this population; this is probably an albinistic shell (Fig. 41F).

### ﻿Group IV: *Rhiostomamorleti* group. Species without detached whorl; breathing device usually expanded at base near suture or canal-shaped

#### ﻿21. Rhiostoma cochinchinensis

Taxon classificationAnimaliaArchitaenioglossaCyclophoridae

(Pfeiffer, 1857)

29E0D508-F4FC-5FDE-B21B-C3FB7AA8F788

Fig. 45A

Cyclostoma (Opisthoporus) cochinchinense Pfeiffer, 1857 [1856]: 337. Type locality: Cochinchina [south of Vietnam].
Opisthoporus cochinchinensis —Pfeiffer 1858: 28. Fischer 1891: 100. Kobelt and Möllendorff 1897: 118.
Pterocyclos cochinchinensis —Reeve 1863: Pterocyclos, pl. 4, species 22.
Cyclotus cochinchinensis —Kobelt 1902: 209. Sutcharit et al. 2019: 19, fig. 4d.

##### Type material.

***Probable syntype***NHMUK 20170354 (1 shell; Fig. 45A) from Cochin China.

##### Diagnosis.

Shell flattened and without detached whorl. Uniformly whitish to pale yellowish shell. Breathing device with incomplete tube shape. Operculum calcareous and low cup-shaped.

##### Differential diagnosis.

This species can be distinguished from *R.asiphon*, especially a yellowish shell form in having a flattened shell and incomplete tube shape. In comparison, *R.asiphon* has a sub-discoidal shell and notch-shaped breathing device.

##### Description.

***Shell*.** Shell slightly thin and flattened, width 36.5 mm, height 12.5 mm. Apex acute; spire slightly elevated. Whorls 4, convex; suture deep; last whorl rounded and stout. Shell surface with fine growth lines. Periostracum corneous and transparent. Shell colour uniformly whitish to pale yellowish and without peripheral band. Breathing device with incomplete tube shape and attached to preceding whorl; detached whorl absent (or inconspicuous). Peristome circular and double; lip thickened and slightly expanded. Aperture opened sub-laterally; outer lip protruded and forming a nearly closed tube; inner lip with deep incision. Umbilicus widely opened. Operculum calcareous, cup-shaped, and multispiral (Fig. 45A).

##### Remarks.

This species was described by Pfeiffer (1857) based on the Cuming collection. Subsequently, Reeve (1863) published the images and re-assigned it to the *Pterocyclos*. Later, Kobelt (1902) redescribed and placed this species into the genus *Cyclotus*, and this treatment has been followed since then. The probable syntype was recently found and illustrated (see Sutcharit et al. 2019). This specimen has a unique breathing device and calcareous cup-shaped operculum with elevated lamellae, suggesting that it is a member of the genus *Rhiostoma*. In addition, the uniformly whitish to pale yellowish shell without any dark brown bands or streaks of this specimen probably represents the albinistic form. However, we maintain this as a valid species despite no additional specimens, and have assigned it to the genus *Rhiostoma*.

The collection locality was from “Cochinchina” (Pfeiffer 1857), which refers to the approximate area of southern Vietnam and southeast Cambodia. The recent land snail survey from southern Cambodia recovered no specimens (Sutcharit et al. 2020), and an intensive survey in southern Cambodia and southern Vietnam is essential for clarifying the systematic status of this species.

#### ﻿22. Rhiostoma cambodjense

Taxon classificationAnimaliaArchitaenioglossaCyclophoridae

(Morelet, 1875)

FEC65B29-20D3-56C0-BB61-F5830F875C58

Figs 44 , 45B–F , 53A

Pterocyclos cambodjensis Morelet, 1875: 286, 287, pl. 13, fig. 1. Type locality: Battambang, Cambodje [Cambodia]. Breure et al. 2018: 232, figs 173, 174.
Opisthoporus pulchellus Morlet, 1889: 154, 188–189, pl. 6, fig. 5. Type locality: Mount. Sisophon (Siam) [Serei Saophoan, Banteay Meanchey, Cambodia]. Fischer 1891: 100. Kobelt and Möllendorff 1897: 119. Morlet 1904: 376, 377, pl. 21, figs 6, 6a. Fischer and Dautzenberg 1904: 427. New synonym.
Opisthoporus cambodjensis —Fischer 1891: 100. Fischer and Dautzenberg 1904: 427.
Cyclotus (Siphonocyclus) pulchellus —Kobelt 1902: 210. Kobelt 1911: 815, pl. 121, figs 1, 2.
Rhiostoma cambodjense —Kobelt 1902: 177. Kobelt 1911: 763, 764, pl. 113, figs 14–16. Sutcharit et al. 2019: 16, fig. 3i.

##### Type material.

***Syntype***NHMUK 1893.2.4.766 (1 shell; Fig. 45B) from Battambang. ***Syntype***MNHN-IM-2000-9828 (1 shell; Fig. 45C) of *Opisthoporuspulchellus* Morlet, 1889, from Mont Sisophon (Siam).

##### Other material examined.

**Cambodia**: Thammaban Khiri Temple, Serei Saophoan (Sisophon), Banteay Meanchey: CUMZ 10203/2 (Fig. 45D). Wat Kirirom Phnombak, Serei Saophoan (Sisophon), Banteay Meanchey: CUMZ 10203/1. **Thailand**: Tham Khao Chakan, Khao Chakan, Srakeo: CUMZ 3810, 3953, 3854, 3856, 3860, 4714 (Figs 45E, F, 53A), 4734, 4813, 10122, 10123, 10124. Wat Khao Panom Sarapee, Khlong Had, Srakeo: CUMZ 10212.

##### Diagnosis.

Shell small, heliciform, detached whorl absent or inconspicuous, and breathing device notch-shaped. Peristome slightly thickened but not expanded, lip thickened, Shell colour yellowish to dark brown with zigzag patterns. Operculum calcareous, low cup-shaped.

##### Differential diagnosis.

*Rhiostomacambodjense* can easily be distinguished from all other known *Rhiostoma* species (except *R.gnomus* sp. nov.; for further comparison, see under that species) in having heliciform shell and narrow umbilicus. Although this species is superficially similar to *Cyclotus* with a heliciform shell, the distinguishing characters are a notch-shaped breathing device, outer lip not expanded, and cup-shaped operculum with elevated lamella and straight lateral fringe. In contrast, the heliciform *Cyclotus* tend to have an expanded outer lip, a concave operculum without elevated lamella, and a deep groove on the lateral fringe (Table 2).

##### Description.

***Shell*.** Shell small, width 16.5–18.7 mm, height 10.7–12.9 mm, thick or thin, and heliciform shape. Apex acute with dark colour; spire elevated. Whorls 4 to 5, convex, and increasing regularly; suture wide, shallow and with or without white subsutural band; last whorl rounded. Shell surface nearly smooth with fine growth lines. Periostracum thin, corneous, and transparent. Shell colour uniformly brownish, black, or with brown zigzag patterns, with narrow dark spiral band on periphery. Detached whorl usually absent (sometimes very short). Peristome circular and double; lip slightly thickened and expanded. Aperture opened laterally. Breathing device notch-shaped; outer lip protruding, with narrow groove; inner lip with shallow incision. Umbilicus narrowly opened and deep. Operculum calcareous, low cup-shaped, and multispiral (Fig. 45B–F).

***Radula*.** Taenioglossate radula arranged in inverted V-shaped row. Central tooth with well-developed central cusp and two lateral cusps on each side; central cusp large with pointed tip; lateral cusps triangular and tapering in size. Lateral teeth consisting of four cusps; central cusp large, triangular tip, and flanked by pointed tips of two inner cusps and one outer cusp. Inner and outer marginal teeth each composed of three cusps; central cusp large and pointed tip, and flanked by pointed tips of one inner cusp and one outer cusp (Fig. 53A).

##### Distribution.

The previous records of this species were only from Cambodia (Morelet 1875; Morlet 1889; Kobelt 1911). The recent record of this species is from Serei Saophoan in Banteay Meanchey Province and Battambang Province, Cambodia, and Srakeo Province in Thailand (Fig. 44).

##### Remarks.

*Rhiostomacambodjense* was described based on a specimen collected from Battambang in Cambodia (Morelet 1875). The syntype NHMUK 1893.2.4.766 (Fig. 45B) seems to have an eroded periostracum and outer shell surface, where only a dark apex and traces of brownish colour pattern remain on the upper shell surface. However, the calcareous and low cup-shaped operculum with elevated lamellae present on the syntype clearly show the characters of the genus *Rhiostoma*.

The original description of *Opisthoporuspulchellus* Morlet, 1889 includes an illustration of a specimen without an operculum. In this study, we re-visited the type locality and found that the specimens have a calcareous and low cup-shaped operculum, diagnostic of the *Rhiostoma*. In contrast, the syntype of *Opisthoporuspulchellus* Morlet, 1889 has a heliciform shell without a detached whorl, and a breathing device with notch shape. In addition, the type locality is Sisophon, whereas that of *R.cambodjense* is Battambang. These two localities are very close to each other (~70 km), and the type specimens are almost identical in shell characters except the brownish shell colour. Therefore, we consider *Opisthoporuspulchellus* Morlet, 1889 as a junior subjective synonym of *R.cambodjense*.

#### ﻿23. Rhiostoma morleti

Taxon classificationAnimaliaArchitaenioglossaCyclophoridae

Dautzenberg & Fischer, 1906

B0184368-8BD9-5701-858B-3AA8CADA8447

**Figure 43.**
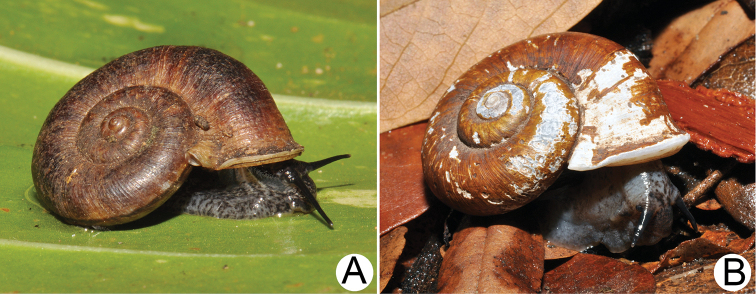
Live snails of the species group IV **A***Rhiostomamorleti*, from Vieng Xai, Houaphanh, Laos **B***Rhiostomalaosense* sp. nov. from Hineboun, Khammouane, Laos. Photographs: K. Inkhavilay.

**Figure 44.**
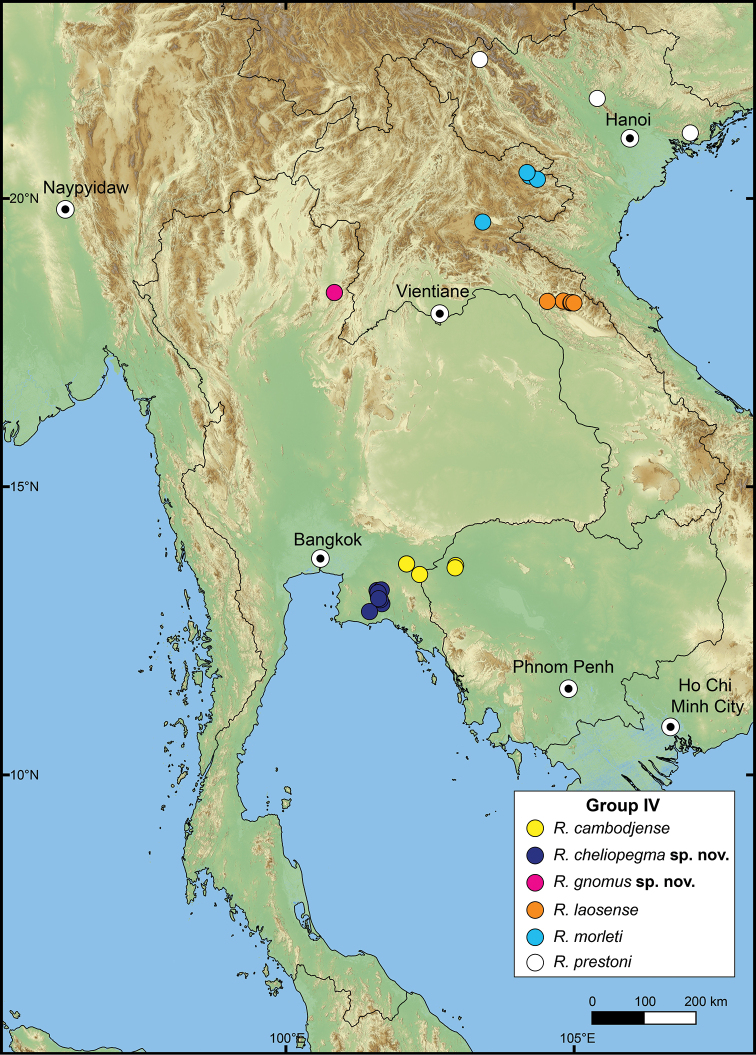
Distribution map of *Rhiostomamorleti*–species group.

Figs 43A , 44 , 46 , 47

Pterocyclos planorbulus Morlet, 1891: 247 (not Lamarck). Locality: Long-son [Lang Son Province, Vietnam].
Rhiostoma morleti Dautzenberg & Fischer, 1906: 429–431, pl. 10, figs 1–4. Type locality: Luang-Prabang, Laos; Ha Giang, Tonkin [Ha Giang Province, Vietnam]. Kobelt 1911: 755, 756, pl. 110, figs 1–4. Thach 2016: 38, fig. 125. Do et al. 2020b: 168, 169, fig. 1g–k.

##### Type material.

***Syntype***MNHN-IM-2000-20961 (1 shell; Fig. 46A) from Laos, chaine de partage entre le Mékong et le Ménam (figured in Dautzenberg and Fischer 1906: pl. 10, figs 1, 2). ***Syntype***MNHN-IM-2000-33837 (2 shells; Fig. 46B) from Ha-Giang. ***Syntype***MNHN-IM-2000-33838 (1 shell; Fig. 46C) from Ha-Giang. ***Syntype***RBINS-MT-658845 (2 shells; Fig. 46D) from Not-Son, Tonkin.

##### Other material examined.

**Laos**: Ban Na Wid, Vieng Xai District, Houaphanh Province: CUMZ 10004/1 (20 shells), 10004/2 (3 shells; Fig. 46E, F). Kraisorn Cave, Vieng Xai District, Houaphanh Province, Laos: CUMZ 10002/1 (15 shells; Fig. 47B, C), 10002/2 (2 shells; Fig. 47A). Km 31, Vieng Xai District, Houaphanh Province: CUMZ 10003 (7 shells; Fig. 47D). Tam Xang (cave), Mueng Kham District, Xiangkhouang Province, Laos: CUMZ 10209 (4 shells; Fig. 47E, F). **Vietnam**: Phong-Tho, Tonkin: SMF 130576 (4 shells), RBINS 659868 (2 shells), 659871 (2 shells).

##### Diagnosis.

Shell large, flattened to depressed shell and without detached whorl. Breathing device incomplete or canal-shaped and pointed apically. Peristome double; outer lip with narrow canal; inner lip with shallow incision or small hole.

##### Differential diagnosis.

This species is similar to *R.cochinchinense* in general shell form, but with an incomplete tube or canal-shaped breathing device, generally brownish to whitish pattern and with a peripheral band, which are the distinguishing characters. In comparison, *R.cochinchinense* has an incomplete tube-shaped breathing device, a uniformly whitish shell, and lacks a peripheral band.

##### Description.

***Shell*.** Shell usually large, width 18.9–28.1 mm, height 8.7–13.0 mm, thickened, and flattened shape. Apex acute; spire flat to slightly elevated. Whorls 4 to 5, convex, increasing regularly; suture narrow and deep; last whorl rounded and stout. Shell surface with fine growth lines. Periostracum thick corneous and brown. Shell colour varies from uniformly whitish or brownish pattern, with narrow to wide dark-brown spiral band on periphery. Detached whorl absent (rarely very short). Peristome circular and double; lip thickened and slightly expanded. Aperture opened sub-laterally. Breathing device incomplete or canal-shaped, protruding apically, and usually not attached to preceding whorl; outer lip protruded and with narrow canal or forming nearly closed tube; inner lip with shallow incision or small hole inside shell. Umbilicus widely opened. Operculum calcareous, cup-shaped, and multispiral (Figs 46, 47).

##### Distribution.

The previous record of this species was from Luang Phrabang, Laos, and Ha Giang and Lang Son, North Vietnam (Dautzenberg and Fischer 1906). Later records were reported from Ha Giang, Yen Bai, Son La, Lai Chau, and Dien Bien provinces, north Vietnam (Do et al. 2020b). This study recorded specimens from several localities in Houaphanh Province, northern Laos (Fig. 44).

##### Remarks.

The identification of this species without the operculum might easily (and wrongly) place it into the genus *Pterocyclos.* Nevertheless, the unique calcareous cup-shaped operculum with elevated lamellae of the type specimen clearly indicates that it is *Rhiostoma*.

The two specimens figured in Do et al. (2015: fig. 4c, d) as *R.morleti* and *Rhiostoma* sp. have the tubular-shaped breathing device that is curved, and with tip attached to the preceding whorl. This character clearly differs from the typical *R.morleti*, but rather should be referred to as *R.simplicilabre*. The specimen figured in Inkhavilay et al. (2019) as *R.morleti* from Bolikhamxay, Laos, shows considerable difference from the type specimen and from the newly collected specimens from Houaphanh Province, northern Laos. Therefore, this study recognises it as *R.laosense* sp. nov.

**Figure 45.**
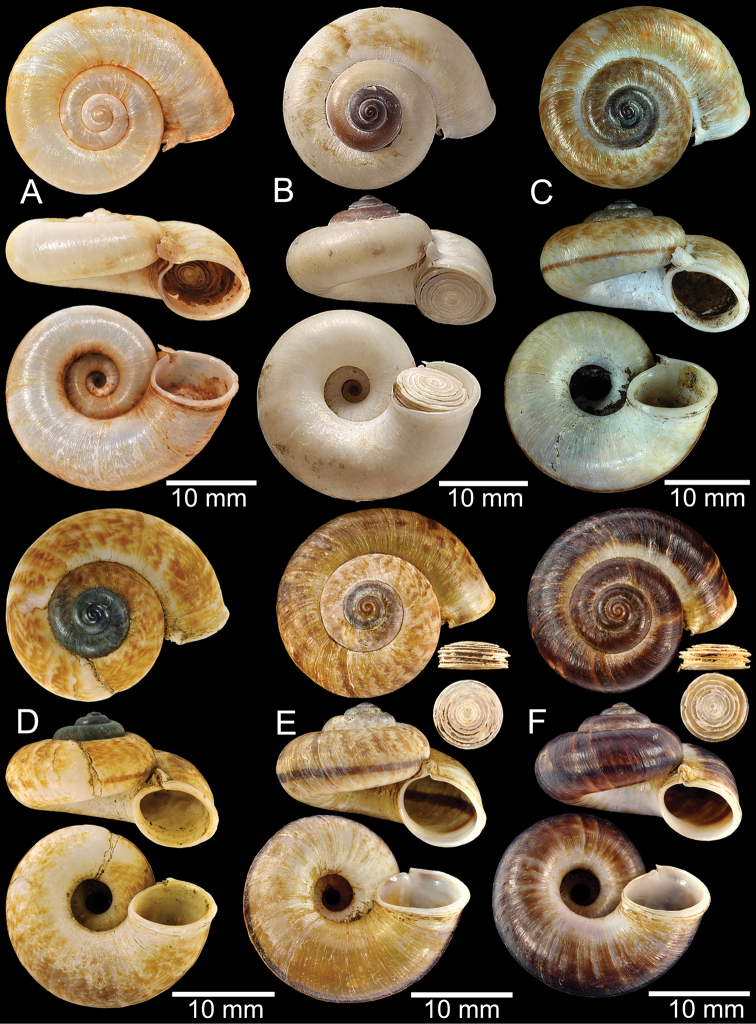
Shell of *Rhiostoma* spp. **A***Rhiostomacochinchinensis* probable syntype NHMUK 20170354 from Cochinchina **B–F***Rhiostomacambodjense***B** syntype NHMUK 1893.2.4.776 from Battambang, Cambodje **C** syntype of “*Opisthoporuspulchellus* Morlet, 1889” MNHN-IM-2000-9828 from Mount. Sisophon **D** specimen CUMZ 10203/2 from Thammaban Khiri, Serei Saophoan (Sisophon), Cambodia **E, F** specimens CUMZ 4714 from Khao Chakan, Srakeo. Photograph: A Robin and V Heros, MNHN (**C**).

**Figure 46.**
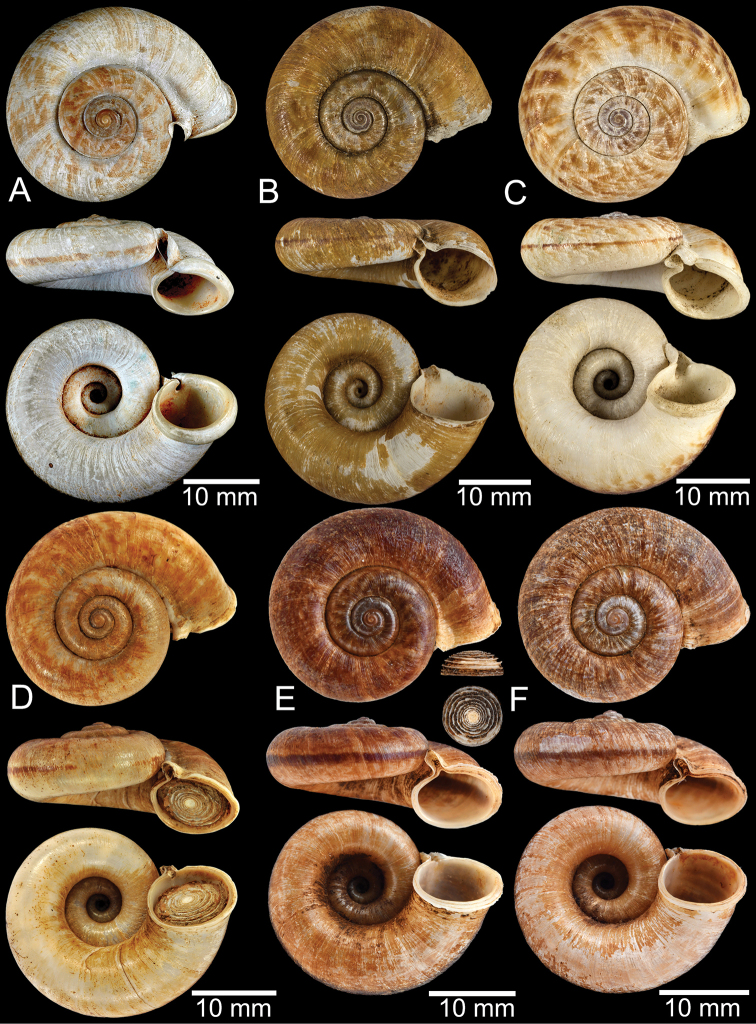
Shell of *Rhiostomamorleti***A** syntype MNHN-IM-2000-20961 from chaine de partage entre le Mékong et le Ménam **B** syntype MNHN-IM-2000-33837 from Ha-Giang **C** syntype MNHN-IM-2000-33838 from Ha-Giang **D** syntype RBINS-MT-658845 from Not-Son, Tonkin **E, F** specimens CUMZ 10004/2 from Ban Na Wid, Vieng Xai, Houaphanh, Laos. Photograph: M Caballer and V Heros, MNHN (**A–C**).

**Figure 47.**
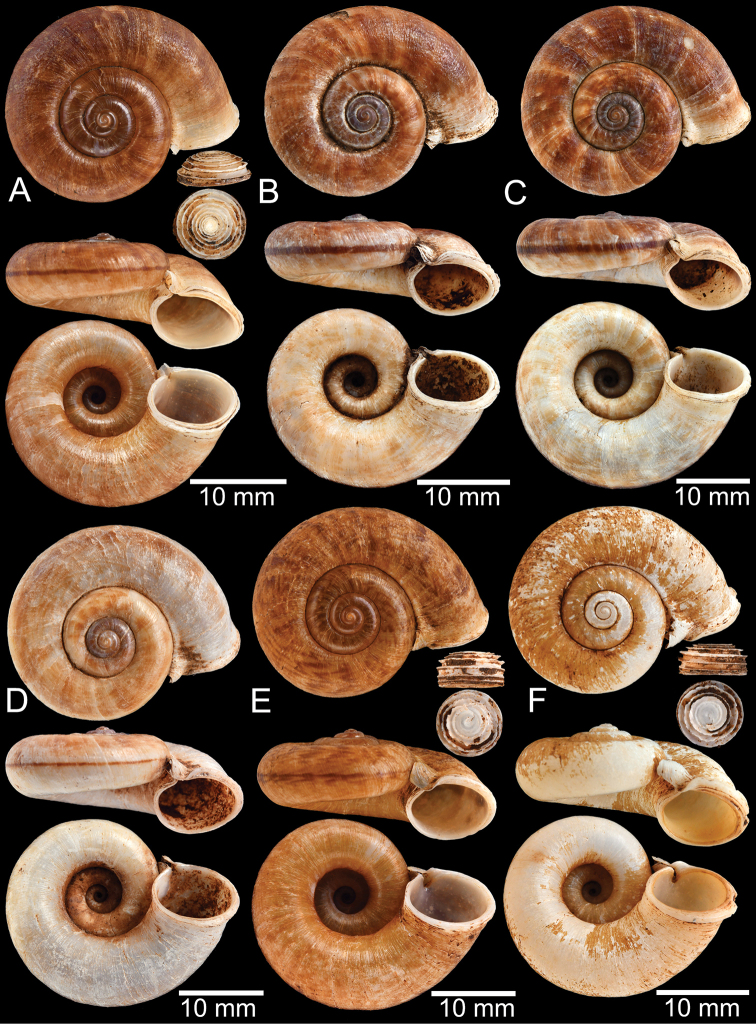
Shell of *Rhiostomamorleti***A** specimen CUMZ 10002/2 from Kraisorn Cave, Vieng Xai, Houaphanh, Laos **B, C** specimens CUMZ 10002/1 from Kraisorn Cave, Vieng Xai, Houaphanh, Laos **D** specimen CUMZ 10003 from Km 31, Vieng Xai, Houaphanh, Laos **E, F** specimen CUMZ 10209 from Tam Xang, Mueng Kham, Xiangkhouang, Laos.

#### ﻿24. Rhiostoma prestoni

Taxon classificationAnimaliaArchitaenioglossaCyclophoridae

(Bavay & Dautzenberg, 1909)

865E931F-A545-5578-8A59-2FD718646F26

Figs 48 , 49

Pterocyclos prestoni Bavay & Dautzenberg, 1909b: 248, 249. Type locality: Binh-Lu, Tonkin [Binh Lieu, Quang Ninh Province, Vietnam]. Bavay and Dautzenberg 1909a: 283, 284, pl. 11, figs 1–3. Kobelt 1913: 969, 970, pl. 112, figs 5–7. Zilch 1956: 172.
Pterocyclos prestoni var. depicta Bavay & Dautzenberg, 1909b: 249. Type locality: Phong-Tho, Tonkin [Phong Tho, Lai Chau Province, Vietnam]. Bavay and Dautzenberg 1909a: 284, pl. 11, fig. 4. Zilch 1956: 173. Kobelt 1913: 970, pl. 112, figs 8–9. New synonym.
Pterocyclos fruhstorferi Kobelt, 1909: 82. Type locality: Chiem-hoa, Tonkin [Chiem Hoa, Tuyen Quang Province, Vietnam]. Kobelt 1911: 739, 740, pl. 107, figs 7–9. Zilch 1956: 172.

##### Type material.

**Figure 48.**
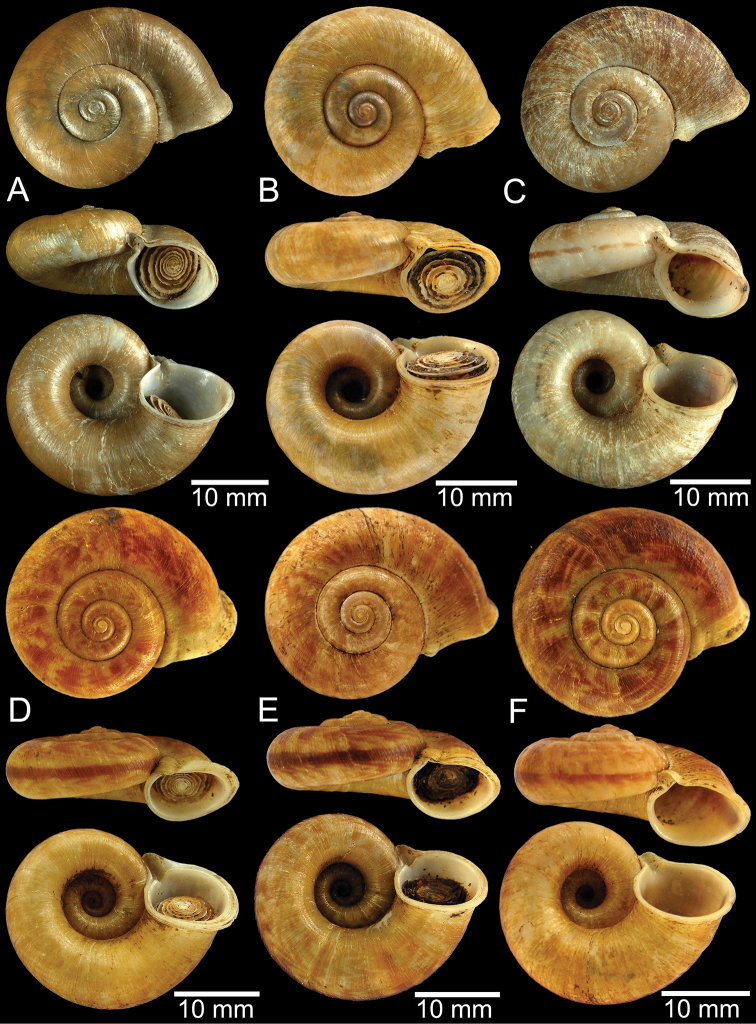
Shell of *Rhiostomaprestoni***A** syntype MNHN-IM-2000-30754 from Binh-Lu **B** syntype RBINS/MT/525354 from Binh-Lu **C–F** originally labelled as “*Pterocyclusprestonidepictus* Bavay & Dautzenberg, 1908” **C** syntype MNHN-IM-2000-30755 from Phong-Tho and **D–F** syntype RBINS/MT/525355 from Phong Tho. Photograph: M Caballer and V Heros, MNHN (**A, C**).

**Figure 49.**
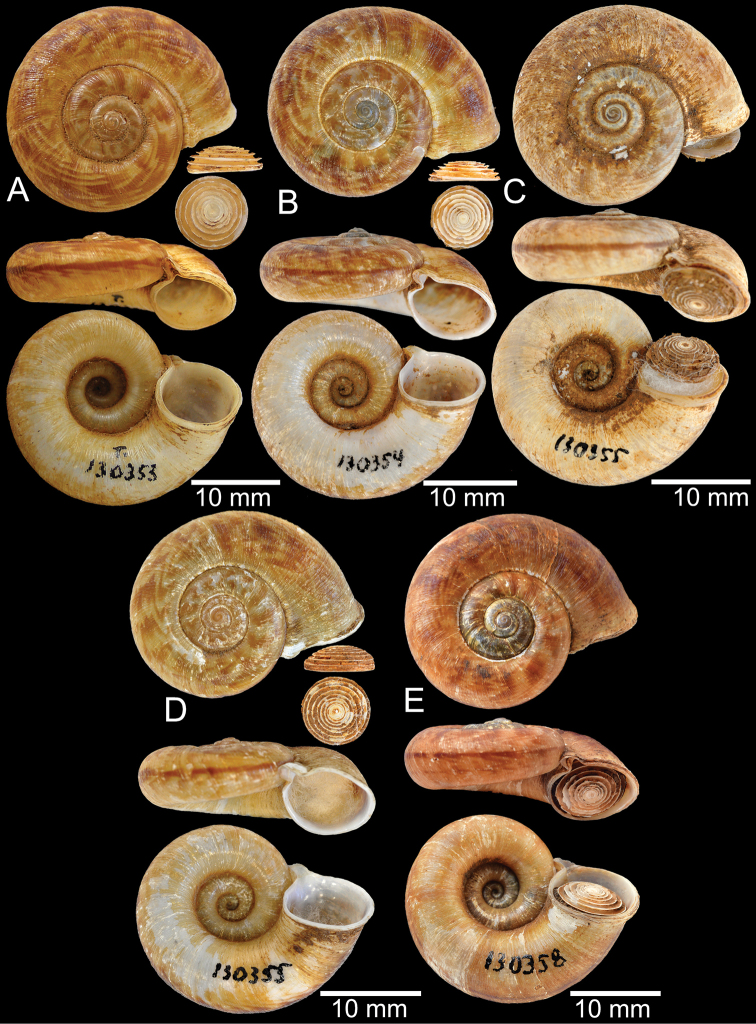
Shell of *Rhiostomaprestoni***A–D** originally labelled as “*Pterocyclosfruhstorferi* Kobelt, 1909” **A** lectotype SMF 130353 from Chiem-hoa, Tonkin **B** paralectotype SMF 130354 from type locality and **C, D** paralectotype SMF 130355 from type locality **E** specimen SMF 130358 from Muong-Hum.

***Syntype***MNHN-IM-2000-30754 (1 shell; Fig. 48A) from Binh-Lu (figured in Bavay and Dautzenberg 1909a: pl. 11, figs 1–3) and ***syntype***RBINS 525354 (1 shell; Fig. 48B) from Binh Lu. ***Syntype***MNHN-IM-2000-30755 (1 shell; Fig. 48C) of Pterocyclosprestonivar.depicta Bavay & Dautzenberg, 1909, from Phong-Tho (figured in Bavay and Dautzenberg 1909a: pl. 11, fig. 4) and ***syntype***RBINS 525355 (3 shells; Fig. 48D–F) from Phong-Tho. ***Lectotype*** (designation in Zilch (1956)) SMF 130353 (1 shell; Fig. 49A) of *Pterocyclosfruhstorferi* Kobelt, 1909, from Chiem-hoa, Tonkin, ***paralectotype***SMF 130354 (1 shell; Fig. 49B) and ***paralectotype***SMF 130355/2 (2 shells; Fig. 49C, D).

##### Other material examined.

**Vietnam**: Trinh-Loung, Tonkin: SMF 130356. Muong-Hum, Tonkin: SMF 130357, 130358 (Fig. 49E). Ba-Xot, Tonkin: NHMW 71770/R/9406 (1 shell). Not-Son, Tonkin: NHMW 71770/R/9405 (1 shell). Tuah-Tuong, Tonkin: NHMW 71770/R/9404 (1 shell). Khe-So, Tonkin: NHMW 47842 (2 shells). Muong Bo, Tonkin: NHMW 71770/R/9407 (1 shell). Bo Xat, Tonkin: NHMW 71770/R/9403 (1 shell).

##### Diagnosis.

Shell large, flattened to depressed shell, and without detached whorl. Breathing device canal-shaped, pointed anteriorly, and attached to preceding whorl. Peristome double, outer lip with wide canal, inner lip with wide and shallow incision. Operculum calcareous, low cup-shaped.

##### Differential diagnosis.

*Rhiostomaprestoni* has a shell morphology that resembles that of *R.morleti*, but it differs in having the canal-shaped breathing device completely attached to the preceding whorl, while *R.morleti* has an incomplete tube or a canal-shaped breathing device not attached to the preceding whorl.

##### Description.

***Shell*.** Shell medium to large, width 22.5–29.1 mm, height 7.8–12.0 mm. Shell thickened, and flattened shape. Apex acute; spire flat to slightly elevated. Whorls 4 to 5, convex, increasing regularly; suture wide and deep; last whorl rounded, stout and wider than penultimate whorl. Shell surface with fine growth lines. Periostracum thick, corneous, and brownish. Shell colour varies from uniformly brownish to brownish zigzag patterns; brownish peripheral band present or absent. Detached whorl absent. Peristome circular and double; lip thickened and slightly expanded. Aperture opened sub-laterally. Breathing device canal-shaped and attached to preceding whorl, causing it to appear without a detached whorl; outer lip protruding, expanded at base and forming wide canal; inner lip with a wide and shallow incision. Umbilicus widely opened. Operculum calcareous, cup-shaped, and multispiral (Figs 48, 49).

##### Distribution.

This species is mainly distributed in Lai Chau, Quang Ninh, and Tuyen Quang provinces of northern Vietnam (Fig. 44).

##### Remarks.

*Rhiostomaprestoni* has long been classified as a member of the *Pterocyclos* (see Kobelt 1911; Zilch 1956). The operculum stuck inside the last whorl of the syntype is a calcareous cup shape and multispiral with elevated lamellae (Figs 48, 49); these are the distinguishing characters of the genus *Rhiostoma* (Fig. 5). Therefore, we have reclassified this species as a member of *Rhiostoma*.

We examined specimens labelled as *R.prestoni* in the RBINS ex. Dautzenberg collection from northern Vietnam that show a high variation in shell colour, from monochrome yellowish to brownish zigzag pattern. However, the shape of the breathing device has less variation than the shell characters and colour patterns. Therefore, we retain *R.prestoni* as a distinct species until the DNA phylogeny becomes available and confirms their status and relationship.

The nominal species, *Pterocyclosfruhstorferi* Kobelt, 1909, was described based on specimens from northern Vietnam. The unique name-bearing type was subsequently designated in Zilch (1956: 172). The lectotype has a shell form similar to that of *R.prestoni*, and the only noticeable difference is the brownish zigzag colour pattern. In contrast, the syntype of *R.prestoni* has a monochrome yellowish brown shell colour. Therefore, we agree with Kobelt (1911) in recognising *P.fruhstorferi* as a junior subjective synonym of *R.prestoni*.

The specimen figured in Do et al. (2015: 122, fig. 4b) as *Pterocyclosprestoni* Bavay & Dautzenberg, 1909 exhibits a corrugated shell surface with nodules arranged on growth lines over the entire shell, and with canal-shaped breathing device protruding apically and attached to preceding whorl. These characters clearly differ from the typical *R.prestoni* and represent an undescribed species. However, this specimen has no operculum for comparison; therefore, the generic placement is still provisional.

Finally, the ‘var. depicta’ was identified based on the distinct shell colour having brownish zigzags or blotches. However, the shell pattern alone cannot be considered the distinguishing character for recognising species or subspecies, since several species of the genus *Rhiostoma* have high variation in shell colour and pattern.

#### ﻿25. Rhiostoma cheliopegma

Taxon classificationAnimaliaArchitaenioglossaCyclophoridae

Tongkerd & Tumpeesuwan sp. nov.

4AD27BA6-93EF-5C3C-8227-360D6DFFE297

https://zoobank.org/99F7CC56-90BD-46F2-B082-2EC1650567CC

Figs 44 , 50 , 53B

Rhiostoma sp. 4—Tumpeesuwan 2001: 76–81, figs 4.28–4.30 (in part).

##### Type material.

**Figure 50.**
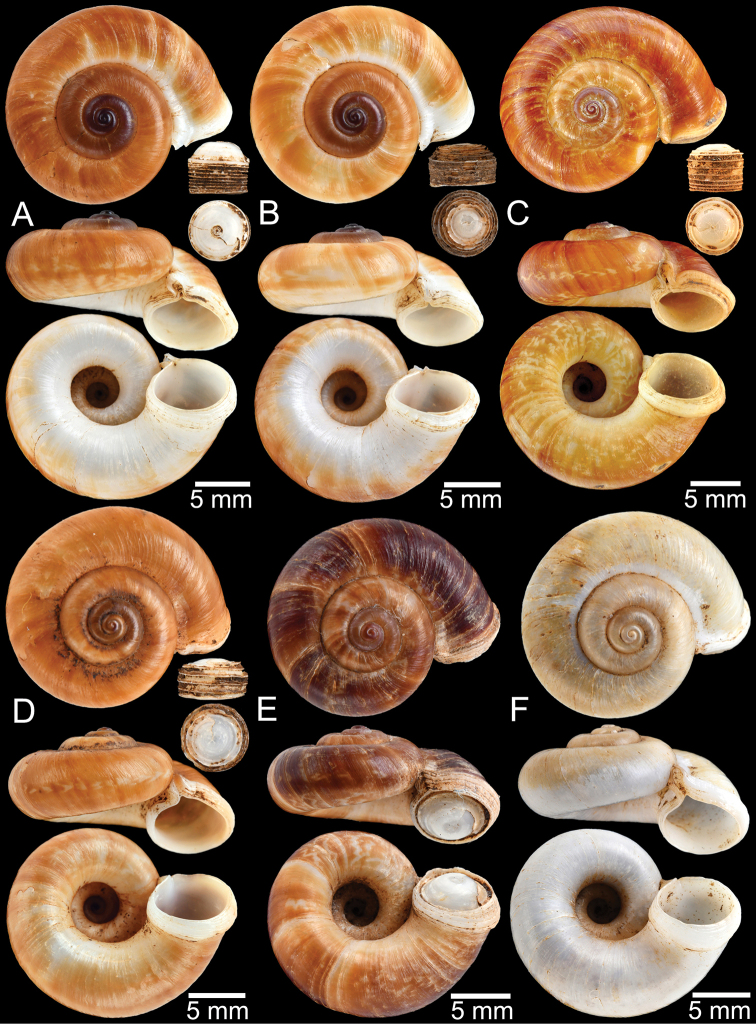
Shell of *Rhiostomacheliopegma* sp. nov. **A** holotype CUMZ 3985/1 from Tham Takien, Khao Chamao, Rayong **B, C** paratypes CUMZ 3985/2 from type locality **D, E** from Tham Khao Loi, Khao Chamao, Rayong **D** specimen CUMZ 4818 and **E** specimen CUMZ 4871 **F** paratype CUMZ 4853 from type locality with albinistic shell form.

***Holotype***CUMZ 3985/1 (W 18.2 mm, H 10.3; Fig. 50A). ***Paratypes***CUMZ 3985/2 (13 shells; Figs 50B, C, 53B), CUMZ 4470 (55 adults + 14 juveniles), CUMZ 4853 (10 shells; Fig. 50F), NHMUK 20220446 (5 shells), and SMF 368681 (5 shells). All paratypes are from the type locality.

##### Type locality.

Isolated limestone hill at Tham Takien, Khao Chamao District, Rayong Province, Thailand (13°05'30.0"N, 101°36'27.7"E).

##### Other material examined.

**Thailand**: Khao Cha-Ang Oun, Bo Thong, Chonburi: CUMZ 3809, 3818, 3861, 3984, 4377, 4448, 4461, 4755/1, 4886, 10053, 10054, 10055, 10057, 10058, 10060. Khao Cha-Ang, Bo Thong, Chonburi: CUMZ 4755/2, 4824, 4847, 4865. Khao Ha Yot, Bo Thong, Chonburi: CUMZ 4833, 10052, 10059, 10082. Khao Tham Mi, Bo Thong, Chonburi: CUMZ 10081. Tham Khao Prathun, Bo Thong, Chonburi: CUMZ 4860. Wat Pluang Thong, Bo Thong, Chonburi: CUMZ 4354, 4355, 4357, 4360. Tham Khao Bost, Khao Chamao, Rayong: CUMZ 4884/2. Tham Neramit Bureau of Monks, Khao Chamao, Rayong: CUMZ 4468, 4876. Wat Ma Diea, Tham Khao Loy, Khao Chamao, Rayong: CUMZ 4467, 4469, 4815, 4818 (Fig. 50D), 4871 (Fig. 50E), 10083. Wat Tham Wattana Mongkon, Khao Chamao, Rayong: CUMZ 4864.

##### Diagnosis.

Shell small, depressed, and without detached whorl. Peristome multi-layered; lip thickened; breathing device notch-shaped. Shell colour usually uniformly reddish to dark brown. Operculum calcareous, thick, tall cup-shaped, and multispiral.

##### Differential diagnosis.

This new species is superficially similar to *R.cambodjense*, and the two species are distributed in nearby geographical areas in eastern Thailand. *Rhiostomacheliopegma* sp. nov. differs from *R.cambodjense* by a sub-discoidal shell, a uniform or blotched reddish brown colour pattern, apertural lip thickened, and expanded tall cup-shaped operculum. For comparison, *R.cambodjense* has a heliciform shell, uniformly brownish or yellowish shell, apertural lip thin, and low cup-shaped operculum. In addition, the COI barcoding in this study suggests separating these two species (Fig. 3).

##### Description.

***Shell*.** Shell small, width 17.1–20.0 mm, height 5.8–11.5 mm, thickened, and sub-discoidal shape. Apex acute with dark colouration; spire slightly elevated. Whorls 4 to 5, convex, increasing regularly; suture wide and shallow; last whorl rounded. Shell surface nearly smooth with fine growth lines. Periostracum thin, corneous, and transparent. Shell colour usually uniformly reddish to dark brownish (rarely with albinistic forms), sometimes with unclear patterns; with narrow dark brown spiral band on periphery. Detached whorl usually absent. Peristome circular and double; lip thickened, expanded and multi-layered. Aperture opened sub-laterally. Breathing device notch-shaped; outer lip protruding, with narrow groove; inner lip with shallow to deep incision. Umbilicus widely opened and deep. Operculum calcareous, thickened, tall and cup-shaped, and multispiral (Fig. 50).

***Radula*.** Teeth arrangement and shape are similar to those of *R.cambodjense*. Central tooth with large central cusp and two lateral cusps on each side. Lateral teeth have four cusps: central cusp large, triangular, and flanked by two small inner cusps and one outer cusp. Marginal teeth each consisting of three pointed cusps (Fig. 53B).

##### Etymology.

The species name *cheliopegma* is derived from two Greek words *cheilos* meaning lip or rim, and *pegma* meaning thickened. This name refers to the strongly thickened apertural lip, which is the distinct character of this species.

##### Distribution.

This new subspecies is known from several isolated limestone hills in Chonburi and Rayong provinces, eastern Thailand (Fig. 44).

##### Remarks.

Despite the COI barcoding seeming to show two subclades (Chonburi and Rayong populations) within this species (Fig. 3). We assign them as conspecific due to the indistinguishable shell form and breathing device and their geographical proximity in eastern Thailand. Therefore, more effort should be paid to additional molecular markers, and morphometric analyses are required to determine whether these distinct subclades should be recognised as a distinct taxon.

#### ﻿26. Rhiostoma gnomus

Taxon classificationAnimaliaArchitaenioglossaCyclophoridae

Tongkerd & Panha sp. nov.

984CE46B-31C6-567D-AD46-BBBB30C83240

https://zoobank.org/FEE72641-8C04-4BF7-A1F7-D7204FD45C29

Figs 44 , 51 , 53C

##### Type material.

**Figure 51.**
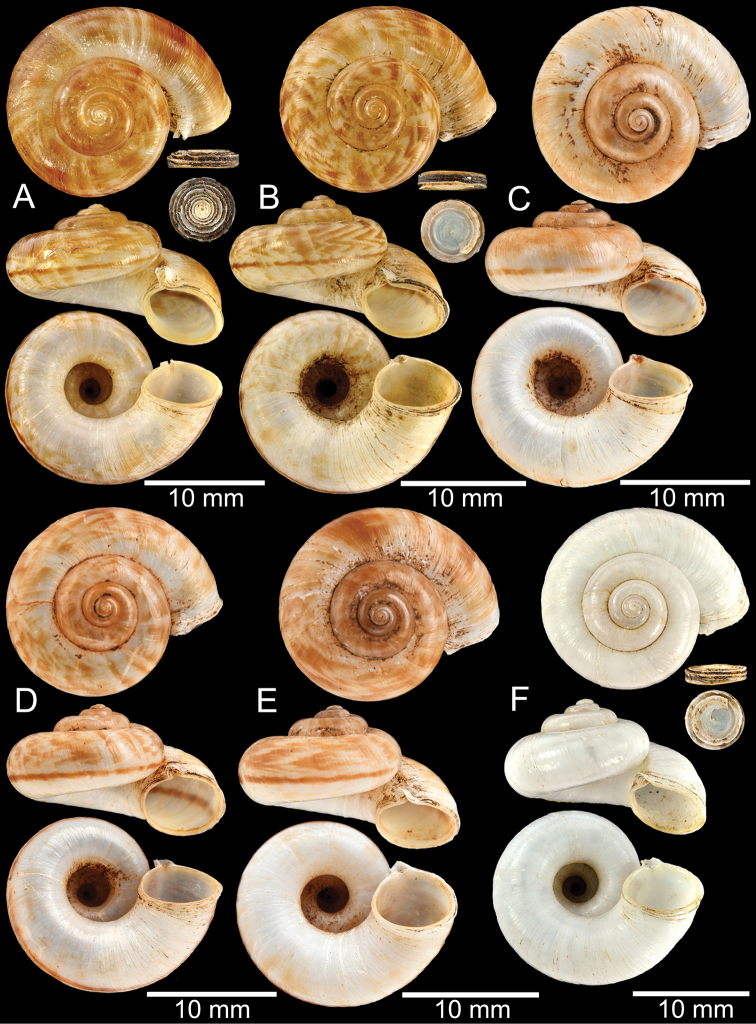
Shell of *Rhiostomagnomus* sp. nov. **A** holotype CUMZ 4716 from Pha Chu, Na Noi, Nan **B–D** paratypes CUMZ 4717 and **E** paratype CUMZ 4849 from type locality **F** paratype CUMZ 4718 from type locality shows albinistic shell form.

***Holotype***CUMZ 4716 (W 16.4 mm, H 10.7; Fig. 51A). ***Paratypes***CUMZ 3922 (55 adults + 31 juveniles), CUMZ 4717 (3 shells; Figs 51B–D, 53C), CUMZ 4718 (3 shells; Fig. 51F), CUMZ 4849 (9 shells; Fig. 51E), CUMZ 4872 (16 adults + 2 juveniles), NHMUK 20220447 (5 shells), and SMF 368682 (5 shells). All paratypes are from the type locality.

##### Type locality.

Limestone hill with dry deciduous forest at Pha Chu (Srinan), Na Noi District, Nan Province, Thailand (18°22'4.6"N, 100°50'23.4"E).

##### Other material examined.

**Thailand**: Sri Nan, Na Noi, Nan: CUMZ 10035.

##### Diagnosis.

Shell small and heliciform; detached whorl shorter than apertural width. Shell colour usually with brownish zigzag patterns. Breathing device notch-shaped. Operculum calcareous, low cup-shaped.

##### Differential diagnosis.

This new species differs from all other members in this species group by having a small and heliciform shell, sometimes with a very short detached whorl and notch-shaped breathing device. Except for *R.cambodjense*, all other species have a flattened or sub-discoidal shell and a canal-shaped breathing device. *Rhiostomagnomus* sp. nov. differs from *R.cambodjense* by having a relatively smaller shell size, lighter shell colour, and apex without dark colour. In contrast, *R.cambodjense* tends to have a larger shell and darker shell colour.

##### Description.

***Shell*.** Shell small, width 14.6–18.2 mm, height 8.9–11.1 mm, thin, and heliciform. Apex acute; spire elevated. Whorls 4 to 5, convex, increasing regularly; suture wide and deep; last whorl rounded. Shell surface smooth or with fine growth lines. Shell colour usually with brownish zigzag patterns or uniformly white to brown; narrow and brownish spiral band usually present (or absent in albinistic forms). Periostracum thin, corneous, and brownish colour. Detached whorl very short and slightly descending. Peristome circular and double; lip slightly thickened and rarely expanded. Aperture opened sub-laterally. Breathing device notch-shaped; outer lip protruding, with shallow groove; inner lip with shallow incision. Umbilicus widely opened and deep. Operculum calcareous, low cup-shaped, and multispiral (Fig. 51).

***Radula*.** Teeth arrangement and shape are similar to those of *R.asiphon*. Central tooth with large central cusp and two lateral cusps on each side. Lateral teeth composed of four cusps; central cusp triangular; two small inner cusps with pointed tips and tapering in size, and one small outer cusp. Marginal teeth each composed of three cusps (Fig. 53C).

##### Etymology.

The species name is derived from the Latin word *gnomus*, meaning dwarf. It refers to the small shell size of the new species.

##### Distribution.

This new species is known only from the type locality at the base of a limestone cliff in Nan Province, Thailand, near the Thai-Laos border (Fig. 44).

##### Remarks.

Although this species is superficially similar to the genus *Cyclotus*, its calcareous, cup-shaped and multispiral operculum with elevated lamellae and its incomplete tube as breathing device on the apertural lip suggests it is a member of genus *Rhiostoma*. Shell colour variation is observed in the type population varied from a brownish zigzag pattern, pale brown blotches to monochrome whitish, or the albinistic shell form found sympatrically within the type population and here recognised as colour variation.

#### ﻿27. Rhiostoma laosense

Taxon classificationAnimaliaArchitaenioglossaCyclophoridae

Tongkerd & Inkhavilay sp. nov.

774527B9-0C86-5E81-8E17-247D28CCB98F

https://zoobank.org/9944EEE3-46DD-42F7-AB77-AF9000C45D57

Figs 44 , 52 , 53D

##### Type material.

**Figure 52.**
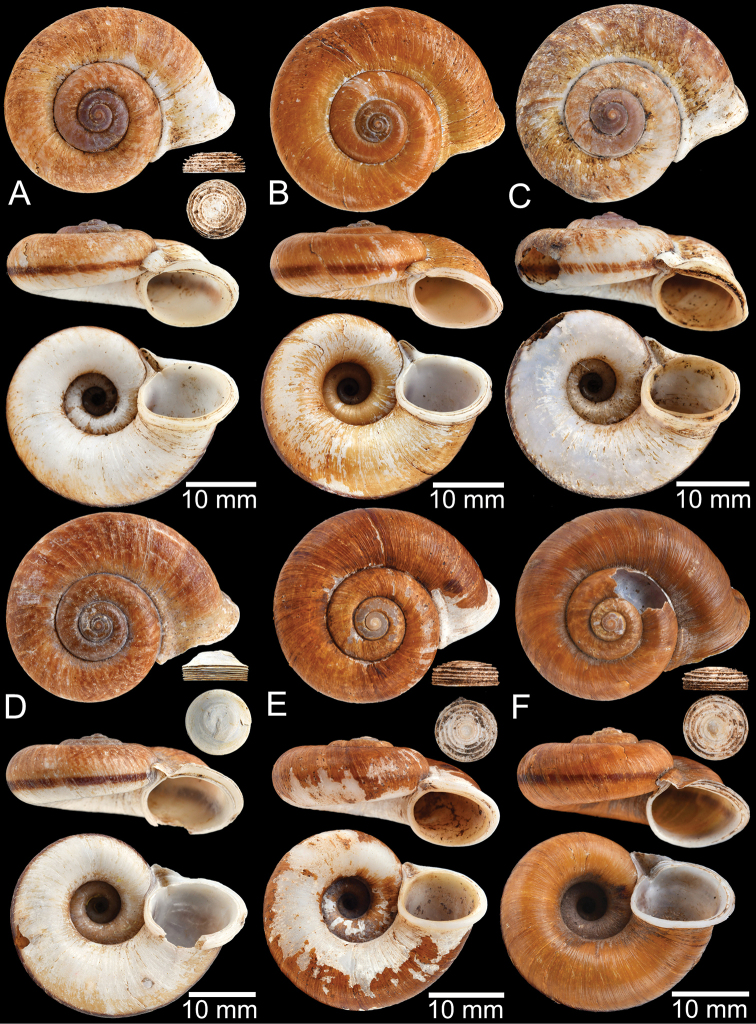
Shell of *Rhiostomalaosense* sp. nov. **A** holotype CUMZ 10001/2 from Wat Pa Pha, Khamkeut, Borikhamxay, Laos **B, C** paratypes CUMZ 10001/3 from type locality **D** specimen CUMZ 4753 from Tam Mangkorn Cave, Lak Sao, Borikhamxay, Laos **E, F** specimens CUMZ 10205 from Tad Muang, Khamkeut, Borikhamxay, Laos.

***Holotype***CUMZ 10001/2 (W 28.7 mm, H 14.2; Fig. 52A). ***Paratypes***CUMZ 10001/1 (23 adults + 2 juveniles), CUMZ 10001/3 (2 shells; Fig. 52B, C), NHMUK 20220448 (5 shells), and SMF 368683 (5 shells). All paratypes are from the type locality.

##### Type locality.

Wat Pa Pha, Khamkeut District, Borikhamxay Province, Laos (18°11'17.3"N, 104°56'26.2"E).

##### Other material examined.

**Figure 53.**
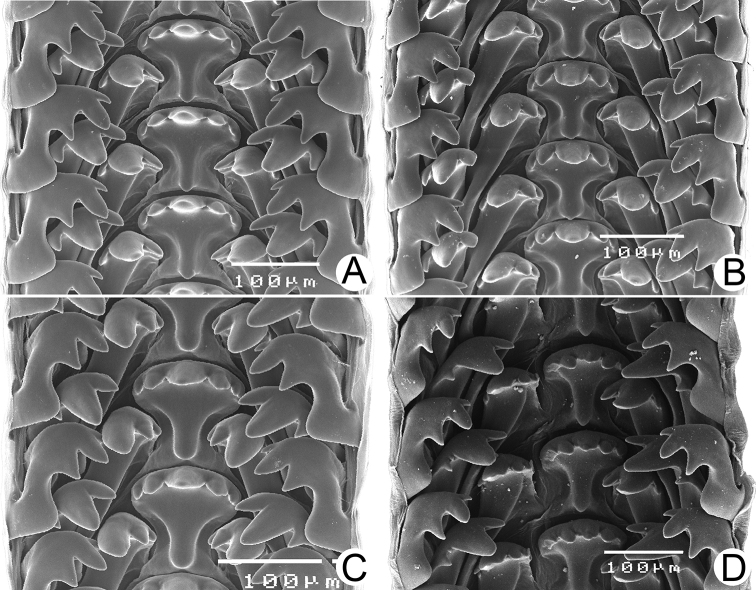
Radula morphology **A***Rhiostomacambodjense*, specimens CUMZ 4714 from Khao Chakan, Srakeo **B***Rhiostomacheliopegma* sp. nov., paratype CUMZCUMZ 3985/2 Tham Takien, Khao Chamao, Rayong **C***Rhiostomagnomus* sp. nov. paratype CUMZ 4717 from Pha Chu, Na Noi, Nan **D***Rhiostomalaosense* sp. nov. specimen CUMZ 10205 from Tad Muang, Khammouane, Laos.

**Laos**: Tam Mangkorn (cave), Khamkeut District, Borikhamxay Province: CUMZ 4753 (2 shells; Fig. 52D), 10005 (17 shells). Tad Muang, Hineboun District, Khammouane Province: CUMZ 10045 (33 shells), 10205 (2 shells; Figs 52E, F, 53D).

##### Diagnosis.

Shell large, depressed, and without detached whorl. Breathing device canal-shaped, pointed anteriorly, and attached to preceding whorl. Peristome double; outer lip with wide canal; inner lip continuous without incision. Operculum calcareous, cup-shaped, and densely lamellate.

##### Differential diagnosis.

This new species is superficially similar to *R.morleti* and *R.prestoni*; the distinguishing characters are the canal-shaped breathing device with outer lip expanded at the base and inner lip continuous without incision, and the operculum with dense lamellae. In contrast, *R.morleti* has an incomplete tube or canal-shaped breathing device with outer lip forming a canal or nearly closed tube, inner lip with shallow incision or a small hole, and operculum with loose lamellae. In addition, *R.prestoni* has a canal-shaped breathing device with a wide and shallow incision on the inner lip.

##### Description.

***Shell*.** Shell large, width 26.7–31.0 mm, height 12.3–15.9 mm, thickened, and flattened to sub-discoidal shape. Apex acute with dark colouration; spire flat. Whorls 4 to 5, convex, increasing regularly; suture wide and deep; last whorl rounded and stout. Shell surface with fine growth lines. Periostracum thin or thick, corneous, dark brown. Shell colour uniformly reddish brown to dark brownish irregular patterns; wide and dark spiral band on periphery. Detached whorl absent. Peristome circular and double; lip thickened, expanded, and multi-layered. Aperture opened sub-laterally. Breathing device canal-shaped, attached to preceding whorl and protruding anteriorly; outer lip protruding, with expanded base and wide canal; inner lip continuous without incision at base of breathing device. Umbilicus widely opened and deep. Operculum calcareous, cup-shaped, and multispiral with dense lamellae (Fig. 52).

***Radula*.** Teeth arrangement and shape are very similar to those of *R.cambodjense*. Central tooth with five cusps; central cusp with similar shape and nearly equal size with two lateral cusps on each side. Lateral teeth are composed of three dull cusps. Marginal teeth each composed of three cusps (Fig. 53D).

##### Etymology.

The species name *laosense* is derived from the type locality Laos, where the type specimen was collected.

##### Distribution.

This new species is known from multiple localities on the massive limestone karsts in Borikhamxay and Khammouane provinces, southern Laos (Fig. 44).

##### Remarks.

No live specimens of this species were found during our surveys. The record from Laos in Inkhavilay et al. (2019) as “*Rhiostomamorleti* Dautzenberg & Fischer, 1906” should be referred to as this new species based on a unique canal-shaped breathing device protruding anteriorly.

### ﻿Taxa with uncertain genus, species group, or undescribed

The three following species are assigned to the genus *Rhiostoma* following literature or the current revision based on their shell characters. They have a detached whorl, distinct breathing device, and calcareous cup-shaped operculum. However, complete or living specimens are still necessary for examination of their systematic relationship and to confirm their generic position.

#### ﻿ Rhiostoma ? americana

Taxon classificationAnimaliaArchitaenioglossaCyclophoridae

Hanna, 1920

25CF2D69-A93E-5606-9390-41BFAE442053

Fig. 55A

Rhiostoma americana Hanna, 1920: 5, 6, pl. 1, fig. 4a, b. Type locality: John Day Basin (Oligocene), Oregon. Roth 1986: 261.

##### Type material.

***Holotype*** UO26890 by monotypy. The author states, “Only the type specimen has been found”, implying that the original description was based on the illustrated specimen. This specimen is accepted as the holotype by monotypy (ICZN 1999: Art. 73.1.2).

##### Remarks.

The shell size is relatively small, with a major shell diameter of 8.5 mm and shell height of 7 mm. The author also stated the strange character of the palatal and columellar teeth; however, these teeth have never been described in recent species. Hanna (1920) also stated that the unique shell shape resembles the Southeast Asian genus *Rhiostoma* at first glance, but that complete specimens, when available, may show that these species belong to another genus. We totally agree with Hanna’s (1920) point of view, as the detached whorl (or detached last whorl), whether it is turning upward or downward, has evolved multiple times in terrestrial snails and it should not constitute a shared derived character (i.e., Páll-Gergely and Neubauer 2020; Chen et al. 2022). Nonetheless, we refrain from formally reclassifying this species, pending further paleontological research. However, the record of *Rhiostoma* in the Nearctic Realm (John Day Formation in Oregon (middle Eocene to late Miocene; William et al. 2015)) is far outside of the known range of the genus. Thus, further study is needed to relocate this species into its correct genus, which probably is the genus *Aperostoma* Troschel, 1847 (Neocyclotidae; see the revision of the genus in Torre et al. 1942).

#### ﻿ Rhiostoma ? amarapuraense

Taxon classificationAnimaliaArchitaenioglossaCyclophoridae

Tongkerd & Panha sp. nov.

A98D72A1-8EAE-5BA8-AED8-8BDD12CB4C8C

https://zoobank.org/2EB032E0-9B39-43C0-BB47-3D113D5C2875

Figs 54 , 55B, C

##### Type material.

***Holotype***SMF 130536/1 (Fig. 55B), collection O. Boettger ex. O. Stoll. Ex. R. Martin. ***Paratype***SMF 130536/2‒8 (7 shells; Fig. 55C), same data as holotype.

##### Type locality.

Amarapura, Burma [Amarapura Township, Mandalay District, Mandalay Region, Myanmar].

##### Diagnosis.

Shell small, thin, and depressed; detached whorl shorter than apertural width. Shell colour with brownish zigzag pattern, without peripheral band. Breathing device with short tubular shape.

##### Differential diagnosis.

Rhiostoma ? amarapuraense sp. nov. can be distinguished from *R.strubelli* by having a depressed shell, outer lip expanded, without a peripheral band, and with a short tubular-shaped breathing device. In contrast, *R.strubelli* from Shan State, Myanmar, displays an elevated spire, with a dark brown peripheral band, thickened multi-layered apertural lip, and breathing device as a short and incomplete tube.

##### Description.

***Shell*.** Shell small, width 20.0–21.5 mm, height 10.0–10.1 mm, thin, and nearly flattened shape. Apex acute and with dark colouration; spire slightly elevated. Whorls 4 to 5, convex, increasing regularly; suture wide and shallow; last whorl rounded. Shell surface with fine growth lines. Periostracum thin, corneous, and transparent. Shell with brown zigzag pattern, without peripheral band. Detached whorl shorter than apertural width. Peristome circular and double; lip slightly thickened and expanded. Aperture opened sub-laterally. Breathing device with short tubular shape; outer lip protruding, with short closed tube and expanded; inner lip with small hole inside aperture. Umbilicus widely opened and deep. Operculum unknown (Fig. 55B, C).

##### Etymology.

The species name *amarapuraense* refers to the collection locality of this new species. In addition, Amarapura is a former capital of Myanmar during the late 18^th^ to early 19^th^ century, but now is a township in Mandalay District, Mandalay Region.

##### Distribution.

**Figure 54.**
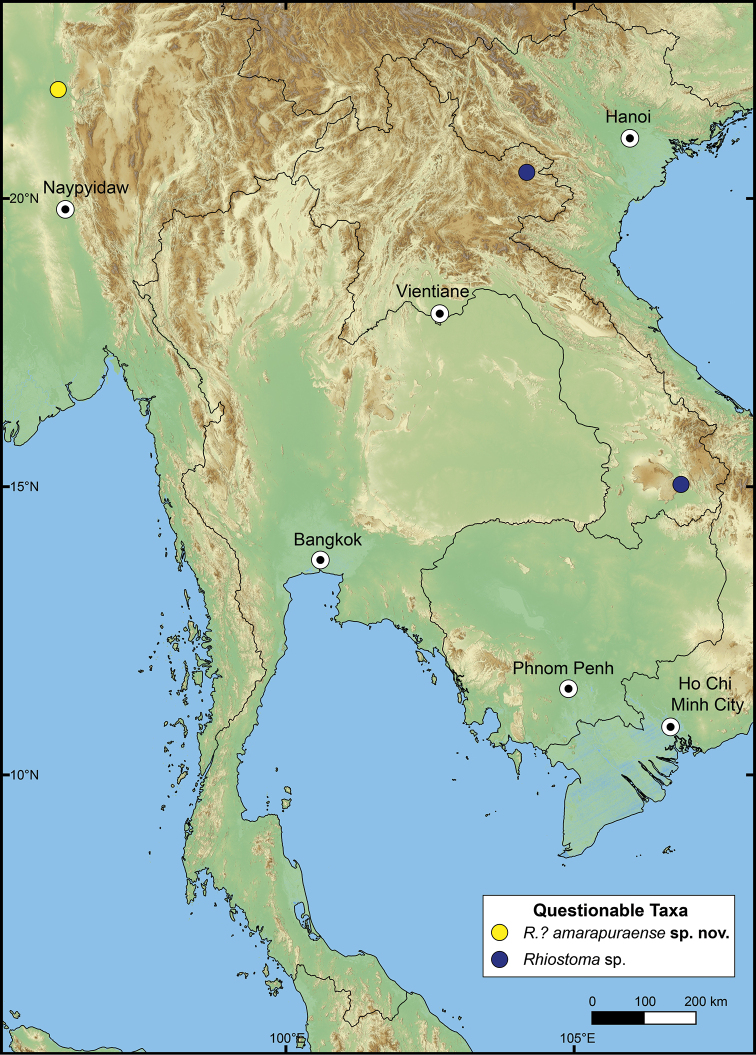
Distribution map of Rhiostoma ? amarapuraense sp. nov. and *Rhiostoma* sp.

This new species is currently known only from the type locality (Fig. 54).

**Figure 55.**
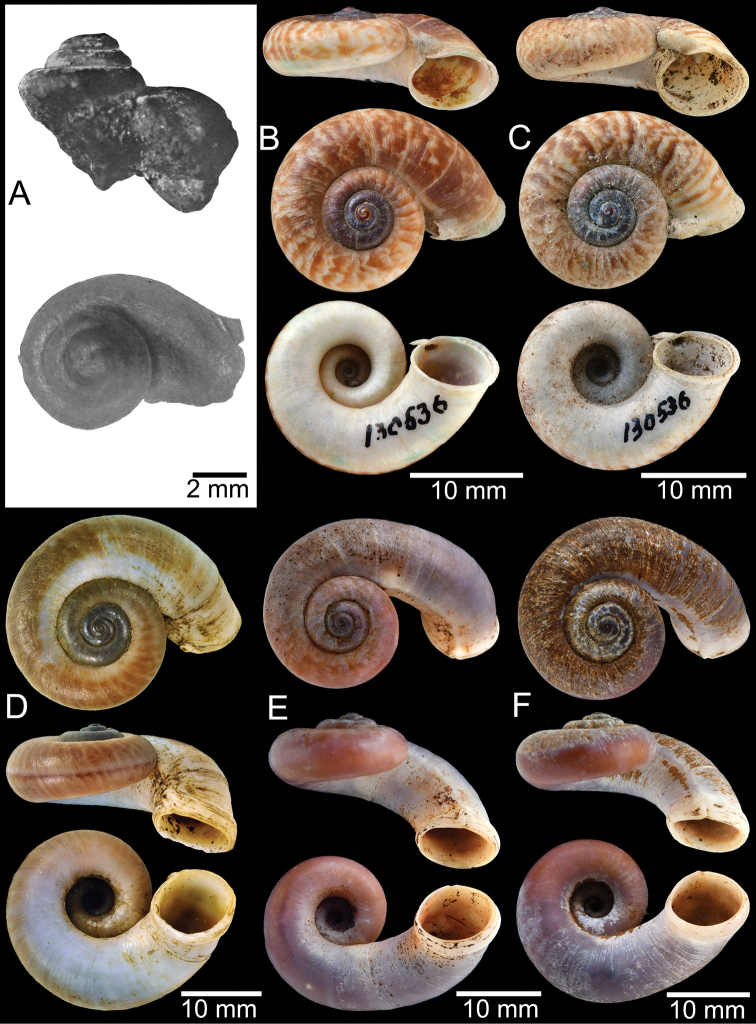
**A**Rhiostoma ? americana, original figure (after Hanna 1920: pl. 1, fig. 4a, b) **B, C**Rhiostoma ? amarapuraense sp. nov. **B** holotype SMF 130536/1 from Amarapura, Burma and **C** paratype SMF 130536/2‒8 from type locality **D–F***Rhiostoma* sp. **D** specimens CUMZ 10050 from Khau Nam Nua, Vieng Xai, Houaphanh and **E, F** specimens CUMZ 10051 from Ban Zox, Xaysetha, Attapeu.

##### Remarks.

This specimen lot SMF 130536 was originally identified as “Pterocycloscf.pullatus Bens.” and was described from Akaouktong, near the Ayeyarwady River, Myanmar. This new species differs from *Pterocyclospullatus* Benson, 1856 in having a depressed shell, short detached whorl, tubular-shaped breathing device, outer lip protruding, with a closed tube, inner lip with a small hole inside the aperture, apertural lip expanded, and widely opened umbilicus. For comparison, the syntype of “*Pterocyclospullatus*” has a helicoid shell, without detached whorl, breathing device notch-shaped, outer lip protruding, with wide groove, inner lip with wide and deep incision, apertural lip blunt (slightly thickened but not expanded), and umbilicus narrow and deep (Preece et al. 2022: fig. 29c).

Although R. ? amarapuraense sp. nov. is nominated based solely on shell morphology without its operculum, all the type series have the unique characters of the *Rhiostoma*, including the peristomal breathing device and the detached whorl. However, the generic placement is still provisional and awaits further collection of living specimens with operculum to confirm generic placement. This species is clearly distinct from other known *Rhiostoma* and *Pterocyclos* species from eastern India and Myanmar.

#### ﻿ Rhiostoma

Taxon classificationAnimaliaArchitaenioglossaCyclophoridae

sp.

E7CB21E8-ACF4-54E2-BFAF-F4A23EDC9185

Figs 54 , 55D–F

##### Material examined.

**Laos**: Khau Nam Nua, Vieng Xai District, Houaphanh Province: CUMZ 10050 (4 shells; Fig. 55D). Ban Zox (village), Xaysetha District, Attapeu Province: CUMZ 10051 (7 shells; Fig. 55E, F).

##### Remarks.

There are two lots of empty shells collected from northern and southern Laos; these specimens have a depressed conical shape and a thickened shell, and the spire is nearly flat and dark in colour. Shell colour is uniformly purplish, with a narrow dark brown peripheral band and thin brownish periostracum. The detached whorl is approximately the same length or longer than aperture width, curved and descending. The aperture is rounded; the lip is thickened and expanded; the breathing device is a knob shape. These characters are nearly identical to the unique characters of *R.samuiense* from southern Thailand and peninsular Malaysia. As no obvious apomorphic characters of these disjunct populations (more than 1500 km apart) are available, future study (especially DNA phylogeny) will be needed to elucidate their systematic position. However, the populations from Laos were found from karst areas with primary forests and without human inhabitants, and therefore are not likely due to recent introduction by humans.

### ﻿Key to the species groups and species of the *Rhiostoma*

**Table:** 

1	Detached whorl with nearly same length or longer than aperture width	**2**
–	Detached whorl absent or shorter than aperture width	**3**
2	Breathing device knob- or notch-shaped. **Group I**	**4**
–	Breathing device incomplete tube or tubular. **Group II**	**6**
3	Detached whorl short; breathing device incomplete tube or notch-shaped. **Group III**	**15**
–	Detached whorl absent; breathing device notch-shaped or canal-shaped. **Group IV**	**21**
4	Detached whorl longer than aperture width	**5**
–	Detached whorl nearly same length to shorter than aperture width	**1. *R.haughtoni***
5	Breathing device knob-shaped; shell colour uniformly purplish to brownish	**2. *R.samuiense***
–	Breathing device notch-shaped; shell colour whitish with brownish zigzag pattern	**3. *R.rhothonotaphrosa* sp. nov.**
6	Detached whorl ≥ 2× apertural width (Fig. 8)	**7**
–	Detached-whorl length < 2× apertural width (Fig. 8)	**11**
7	Periostracum thickened and dark brown to brownish colour; apertural lip expanded on palatal side; breathing device usually tubular (incomplete tube- and notch- shaped also present)	**5. *R.hainesi***
–	Periostracum thin, corneous, transparent; apertural lip evenly expanded; breathing device tubular	**8**
8	Detached-whorl length < 2.5× apertural width (Fig. 8)	**9**
–	Detached-whorl length > 2.5× apertural width (Fig. 8)	**10**
9	Shell uniformly brownish or with irregular blotches; peripheral band present	**7. *R.marioni***
–	Shell uniformly reddish brown to dark brown; peripheral band absent (sometimes present with narrow and faded band)	**9. *R.jalorensis***
10	Sutural ridge indistinct; shell uniformly brown to dark brown; peripheral band absent	**10. *R.thachi***
–	Sutural ridge prominent; shell with dark brown zigzag pattern on dorsal side and some on ventral side; peripheral band present	**12. *R.lannaense* sp. nov.**
11	Peripheral band absent or narrow with brownish colour	**12**
–	Peripheral band very broad and dark colour	**11. *R.ebenozostera* sp. nov.**
12	Periostracum thin corneous	**13**
–	Periostracum thick corneous and brownish colour	**6. *R.simplicilabre***
13	Dark brown zigzag pattern present only on dorsal side of shell	**14**
–	Dark brown zigzag pattern present on both sides of shell	**13. *R.trigrina* sp. nov.**
14	Ventral shell surface whitish, usually without pattern; narrow dark brown peripheral band present	**8. *R.dalyi***
–	Ventral shell surface with paler colour pattern than dorsal surface; peripheral band absent, only narrow brownish dashed line present in some shells	**4. *R.housei***
15	Periostracum thick corneous, translucent to opaque	**16**
–	Periostracum thin corneous, transparent to translucent	**17**
16	Periostracum reddish brown; breathing device short tubular and perpendicular to detached whorl	**17. *R.anceyi* sp. nov.**
–	Periostracum thick corneous and translucent; breathing device short incomplete tube-shaped and nearly perpendicular to detached whorl	**16. *R.abletti***
17	Shell colour generally uniform	**14. *R.asiphon***
–	Shell colour generally with zigzag pattern	**18**
18	Breathing device a short incomplete tube; brownish zigzag pattern is paler or absent on ventral side of shell	**19**
–	Breathing device incomplete tube or short tubular; brownish zigzag pattern present on both sides of shell	**20. *R.platymorpha* sp. nov.**
19	Shell thick, depressed to discoid; lip expanded and multi-layered	**20**
–	Shell thin and sub-discoidal; lip slightly expanded	**15. *R.strubelli***
20	Shell colour whitish with brownish zigzag pattern; breathing device incomplete tube	**18. *R.breviocollar* sp. nov.**
–	Shell colour brownish with dark brown zigzag pattern; breathing device incomplete tube, usually perpendicular to detached whorl	**19. *R.furfurosum* sp. nov.**
21	Shell flattened	**22**
–	Shell sub-discoidal or heliciform	**25**
22	Peripheral band absent; breathing device incomplete tube or short tubular; apertural lip thickened and slightly expanded	**23**
–	Peripheral band present; breathing device incomplete tube or canal shape; apertural lip, outer lip with canal, inner lip with incision or small hole	**24**
23	Shell colour uniformly whitish; breathing device incomplete tube-shaped	**21. *R.cochinchinensis***
–	Shell colour with brownish zigzag pattern; breathing device short and stout tubular	**28. *R. ? amarapuraense* sp. nov.**
24	Breathing device stout incomplete tube or canal-shaped; outer lip with narrow canal; inner lip with narrow incision	**23. *R.morleti***
–	Breathing device canal-shaped; outer lip with wide canal; inner lip with wide incision	**24. *R.prestoni***
25	Shell sub-discoidal	**26**
–	Shell heliciform	**27**
26	Breathing device canal-shaped; peristome double; periostracum thick and dark brown	**27. *R.laosense* sp. nov.**
–	Breathing device notch-shaped; peristome multi-layered and lip thickened; periostracum thin corneous	**25. *R.cheliopegma* sp. nov.**
27	Shell usually uniformly brownish or black and with dark zigzag pattern; early whorls darker colour	**22. *R.cambodjense***
–	Shell usually with brownish zigzag pattern (rarely uniformly white to brown); early whorls without darker colour	**26. *R.gnomus* sp. nov.**

### ﻿Species excluded by this revision

The following species had initially or subsequently been placed under the genus *Rhiostoma*. However, during this revision, we examined the primary type specimens and/or the topotypic specimens and suggest the appropriate generic classification of these species below.

#### ﻿Genus Pterocyclos

Taxon classificationAnimaliaArchitaenioglossaCyclophoridae

Benson, 1832

1C73CAC1-D135-5CD3-862B-41F4174E2DD7

Pterocyclos Benson, 1832: 11. Wenz, 1938: 461. Egorov 2009: 35. Sutcharit et al. 2014: 331–333.
Pterocyclus Agassiz, 1848: 908 [unjustified emendation]. Kobelt 1902: 160, 161. Kobelt 1911: 719. Gude 1921: 97. Egorov 2009: 26, 27.

##### Types species.

*Pterocyclosrupestris* Benson, 1832 by monotypy.

##### Diagnosis.

Shell medium to large, discoidal, conical to nearly flattened. Shell colour uniformly yellowish to with brownish stripes; periostracum thin or thickened and hairy. Terminal part of last whorl usually attached to penultimate whorl or sometimes separated with short detached whorl. Peristome circular and double or sometime boundary hardly visible; lip thickened and expanded. Breathing device on upper junction of peristome; outer peristome slightly to broadly expanded; inner peristome inconspicuous or with a shallow incision. Umbilicus widely opened. Operculum calcareous, low cup-shaped to concave, inside covered with corneous layer, outside with calcareous anticlockwise multispiral with elevated lamella, and lateral straight.

##### Remarks.

The genus comprises a hundred nominal species (MolluscaBase 2022) and is widely distributed from South to Southeast Asia. The comprehensive revision of the genus has never been implemented other than in the classical work by Kobelt (1902, 1911). Seven species have been reported so far from the Malay Peninsula (see Sutcharit et al. 2014). The genus *Pterocyclos* can be distinguished from the *Rhiostoma* by having an expanded outer lip with wing shape and forming an incomplete tube, inner lip with deep and wide incision, inside of operculum with corneous layer and outside of operculum calcified with wide to close lamellae. In contrast, the *Rhiostoma* tend to have a detached whorl, breathing device of various types, outer lip not expanded, and thick calcareous cup-shaped operculum without corneous layer inside and with wide and elevated lamella outside (Table 2, Fig. 5).

#### ﻿ Pterocyclos jousseaumei

Taxon classificationAnimaliaArchitaenioglossaCyclophoridae

(Morgan, 1885)

7279C3DC-A271-5E42-97DD-D938D43BAF25

Fig. 56

Rhiostoma jousseaumei Morgan, 1885: 400, pl. 8, fig. 2. Type locality: la haute vallée de Kinta (Pérak) [Kinta valley, Perak, Malaysia]. Möllendorff 1891: 340. Kobelt 1902: 178. Kobelt 1911: 758, pl. 110, figs 11–13. Laidlaw 1928: 31. Chang 1995: 5 with text figure. Foon et al. 2017: 24, fig. 8f.
Rhiostoma macalpine–woodsi Laidlaw, 1939: 166, with text figure. Type locality: Sungei Siput, Perak. Sutcharit et al. 2019: 37, fig. 8g.

##### Type material.

**Figure 56.**
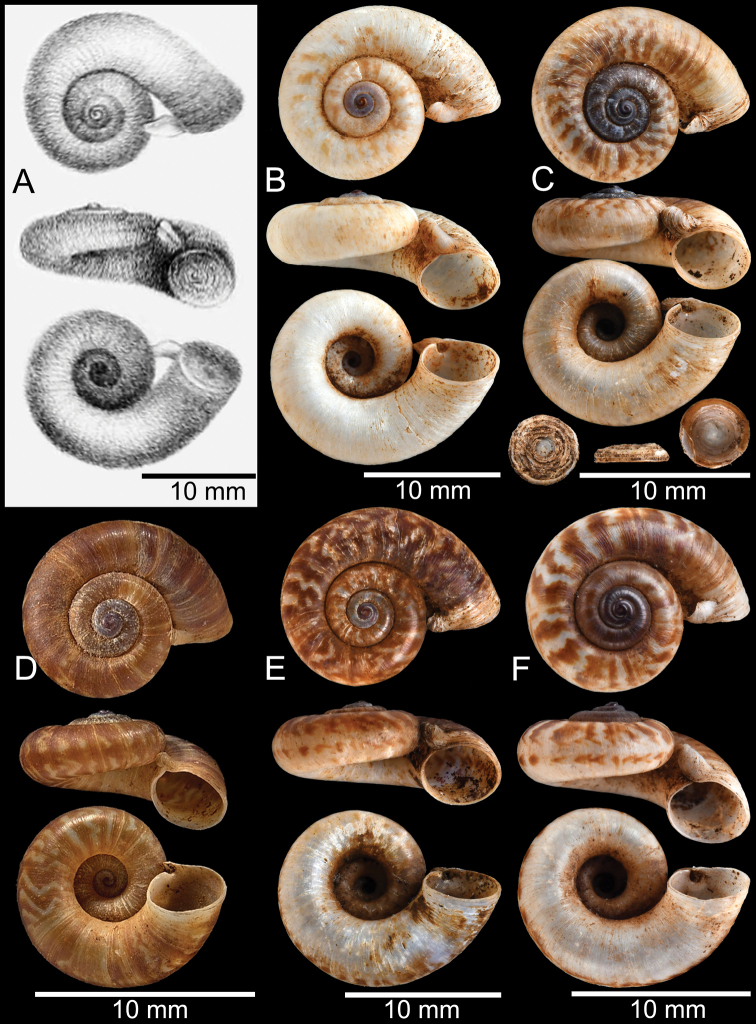
Shell of *Opisthoporusjousseaumei***A** original figure (after Morgan 1885: pl. 8, fig. 2) **B** specimen CUMZ 4752 from Ipoh, Perak, Malaysia **C** specimen CUMZ 7457 from Kinta, Perak, Malaysia **D** lectotype of “*Rhiostomamacalpinewoodsi* Laidlaw, 1939” NHMUK 1939.4.13.23 from Sungei Siput, Perak **E, F** specimens CUMZ 10213 from Gunung Dato, Ipoh, Perak, Malaysia.

The type specimens *Rhiostomajousseaumei* Morgan, 1885 could not be located in the MNHN collection. ***Lectotype*** (designation in Sutcharit et al. (2019)) NHMUK 1939.4.13.23 (Fig. 56D) of *Rhiostomamacalpinewoodsi* Laidlaw, 1939, from Sungei Siput, Perak.

##### Other material examined.

**Malaysia**: Specimen CUMZ 4752 (2 shells; Fig. 56B) from Ipoh, Perak State. CUMZ 7457 (15 shells; Fig. 56C) from Tanjung Rambutan, Kinta, Perak State. CUMZ 10213 (5 shells; Fig. 56E, F) from Gunung Dato, Ipoh, Perak State.

##### Remarks.

Periostracum thin, corneous, shell varying from uniform colour to dark brown zigzag pattern; periphery with a thin spiral band. Detached-whorl length similar to apertural width. Peristome circular and double or boundary hardly visible; lip thick or thin, and not expanded to slightly expanded. Breathing device with incomplete tube or tubular shape, curved posteriorly and its tip usually attached to preceding whorl. Operculum calcareous, multispiral, and slightly concave on both surfaces.

**Figure 57.**
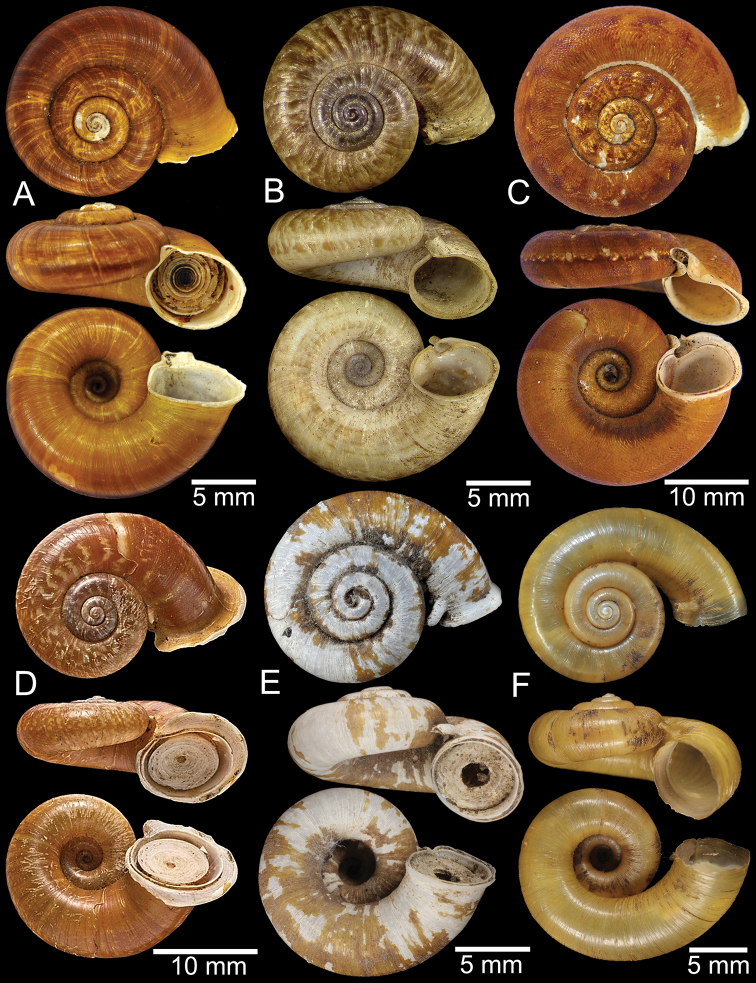
**A***Pterocyclossubalatus* Sykes, 1903, holotype UMZC 1032 from Gunong Inas, Perak, Malay Peninsula **B***Pterocyclosspelaeotes* (Tomlin, 1931), lectotype NMW 1955.158.01107 from Batu Cave, Kula Lumpur, Selangor **C***Pterocyclosumbraticus* (Benthem Jutting, 1949), holotype ZMA 135622 from Maxwell’s Hill, Perak, Malaysia **D***Cyclotusboxalli* syntype NHMUK 1894.5.23.1 from Kina Balu **E***Opisthoporusbattambangensis* holotype MNHN-IM-2000-35506 from Battambang, Cambodia **F***Opisthoporusgrohi* holotype MNHN-IM-2000-35507 from Dak Nong, Vietnam. Photograph: H Wood, NMW (B), M Caballer, A Lardeur and V Heros, MNHN (**E, F**).

An operculum accompanied the examined specimen and was also figured in Morgan (1885): it was multispiral and plate-shaped (Fig. 56A, C). With these observations, we suggest the re-assignment of this species to the *Pterocyclos*. This species can be separated from *Pterocyclossubalatus* Sykes, 1903, *Pterocyclosspelaeotes* (Tomlin, 1931), and *Pterocyclosumbraticus* (Benthem Jutting, 1949) from peninsular Malaysia in having the apertural lip thickened and usually not expanded (rarely slightly expanded), while the three species (Fig. 57A–C) tend to have a widely expanded lip (see also Sutcharit et al. 2014). Furthermore, *P.subalatus* has no detached whorl but has a wide canal-shaped breathing device attached to the preceding whorl; *P.umbraticus* has a larger shell (width 27 mm), without a detached whorl, and an undulated shell surface.

Laidlaw (1939) stated that *R.macalpinewoodsi* differed from *R.jousseaumei* in having a relatively small shell (height 6 mm, width 12 mm), with slightly thickened but not expanded apertural lip, a breathing device channel-like to short tubular [= incomplete tube] shape. Later, Foon et al. (2017) examined several specimens and found an intermediate shell form; the type locality of both nominal species is situated in the Kinta Valley, Perak State, peninsular Malaysia. In addition, the specimens examined herein show a mix between smaller shell (with incomplete tube) and larger shell (with tubular) forms in a single collection locality, with the intermediates as reported in Foon et al. (2017). This distinctive shell size and breathing device could be attributed to intraspecific variation, and examination of more specimens and systematic study will help resolve this issue.

#### ﻿Genus Cyclotus

Taxon classificationAnimaliaArchitaenioglossaCyclophoridae

Swainson, 1840

FB7C1438-22D1-5D3A-B18A-575862342483

Cyclostoma (Cyclotus) Swainson, 1840: 186, 336.
Cyclotus —Kobelt 1902: 188, 189. Kobelt 1911: 773, 793. Egorov 2009: 26.

##### Type species.

*Cyclotusvariegatus* Swainson, 1840 by subsequent designation by Pfeiffer (1852: 16).

##### Diagnosis.

Shell small to large, low conical to flattened. Shell colour uniformly brownish or with irregular brownish stripes; periostracum thin or thick corneous. Terminal part of last whorl usually attached to penultimate whorl, and rarely with short separation. Peristome circular and double or multi-layered; lip thick or thin and expanded and reflexed. Breathing device sometimes absent; if present at upper junction of peristome with wing-like projections, canal or completely tubular structure located in suture behind apertural lip. Umbilicus wide. Operculum calcareous, somewhat flattened, or plate-shaped, lateral fringe deeply grooved, slightly concave externally, anticlockwise multispiral, nucleus central.

##### Remarks.

This is one of the most speciose genera among the tribe Cyclotini. The authenticated specimen of the type species (Fig. 5, said to be “Type” ex. Cuming coll. from Palawan) is figured herein for detailed comparison. Traditionally, the genus was divided into six subgenera but has been subject to revision since Kobelt (1902, 1911). The genus comprises more than 100 nominal species (MolluscaBase 2022), which have wide distribution ranging from southern China to Japan, Southeast Asia, and New Guinea (Kobelt 1902). Since the last compilation in Kobelt (1902, 1911–1914), no comprehensive revision has been carried out, only the descriptions of new species. Therefore, systematic research based on morphology and molecular data from its range is essential and urgent to reveal its systematics.

#### ﻿ Cyclotus boxalli

Taxon classificationAnimaliaArchitaenioglossaCyclophoridae

(Godwin-Austen, 1893)

29B7AC8F-4D02-5CEE-B106-C8C5680E67F2

Fig. 57D

Rhiostoma boxalli Godwin-Austen, 1893: 32, 33, fig. 1a–c. Type locality: Near Kina Balu; Palawan. Kobelt 1902: 538, 539. Sutcharit et al. 2019: 13, 14, fig. 3b, c.
Pterocyclos boxalli —Vermeulen and Liew 2022: 65, 66, fig. 34a–d.

##### Type material.

***Syntype***NHMUK 1894.5.23.1 (1 shell; Fig. 56A) from near Kina Balu [Mount Kinabalu, Sabah, Malaysia] and ***syntype***NHMUK 1895.12.5.34 (1 shell) from Palawan [Palawan Islands, Province of Palawan, Mimaropa Region, The Philippines].

##### Remarks.

Originally this species was thought to belong to *Rhiostoma*, and this classification was followed by Kobelt (1902) until the recent type catalogue (Sutcharit et al. 2019). The type specimens (Fig. 57D; see also Sutcharit et al. 2019: 3b, c) appear clearly distinct from the generic diagnosis by having a calcareous operculum with slightly concave outer and inner surfaces, being multispiral, and lamella not elevated. The outer peristome of the breathing device is expanded as a wing at the upper junction and has a short tubular shape, located in the suture and away from the apertural lip; the inner peristome has a shallow incision. In addition, a short, detached whorl and a broadly expanded lip are also present in this species.

Recently, this species was relocated to the *Pterocyclos* (Vermeulen and Liew 2022); however, it has a shallow incision on the inner peristome, the outer lip expanded with a wing, it has a short tubular breathing device, and the operculum is plate-shaped, without elevated lamellae or deep grooves on the lateral fringe. These unique characters suggest that this species is more similar to *Cyclotus* than *Petrocyclos* (see also Table 2, Fig. 5). Because of all these differences, we suggest the re-assignment of this species to the genus *Cyclotus*.

#### ﻿Genus Opisthoporus

Taxon classificationAnimaliaArchitaenioglossaCyclophoridae

Benson in Pfeiffer, 1851

EEBF4694-25E5-57DA-AB6A-83B34EF4B068

Cyclostoma sect. Opisthoporus Benson in Pfeiffer, 1851: 8.
Cyclotus (Opisthoporus) —Kobelt 1902: 213. Kobelt 1912: 834, 835. Egorov 2009: 26, 27.
Opisthoporus —Low and Tan 2017: 15, 16. Do et al. 2020a: 104.

##### Type species.

*Cyclostomataylorianum* Pfeiffer, 1851. Type species by subsequent designation by Kobelt (1912: 834).

##### Diagnosis.

Shell small to medium, discoidal, spire varies from conical to nearly flattened. Shell colour uniformly yellowish to with brownish stripes; periostracum thin or thickened and hairy. Terminal part of last whorl attached to penultimate whorl or separated with short to long detached whorl. Peristome circular and double or sometimes with boundary inconspicuous; lip thick or thin and slightly to greatly expanded. Breathing device with tubular shape and located behind apertural lip; outer peristome slightly to broadly expanded; inner peristome inconspicuous or with a shallow incision. Umbilicus widely opened. Operculum calcareous, usually concave on both sides or plate-shaped, multispiral, and lateral fringe grooved.

##### Remarks.

This genus was nominated based on *Cyclostomataylorianum* Pfeiffer, 1851 (which is a synonym of *Opisthoporusbiciliatus* (Mousson, 1849)) as the type species. Recently, Do et al. (2020a) took *Opisthoporus* out of synonymy and raised it to full generic rank, provided quite comprehensive and clear diagnostic characters, and added new species to the genus, the first since Kobelt (1912). Nevertheless, the reliability and relationship of the *Opisthoporus* based on their conchology has not been thoroughly confirmed by other evidence, especially molecular analyses. Further research will undoubtedly be needed to clarify these systematic positions and affinities with other genera.

#### ﻿ Opisthoporus battambangensis

Taxon classificationAnimaliaArchitaenioglossaCyclophoridae

(Thach & Huber, 2020)

2CB2612F-1288-5FC0-BB61-101462F92B7F

Fig. 57E

Rhiostoma battambangensis Thach & Huber in Thach, 2020: 18, figs 153, 154. Type locality: Battambang City, Battambang Province, Cambodia.

##### Type material.

***Holotype***MNHN-IM-2000-35506 (Fig. 57E) from Battambang City, Battambang Province.

##### Remarks.

This species was described based on the holotype, and by three paratypes housed in the authors’ collections. The operculum is attached with the holotype and has a calcareous, multispiral, and plate-shaped operculum; the shell has a short detached whorl and yellowish brown periostracum. The outer peristome is slightly expanded with short tubular breathing device located on the suture area of the detached whorl, and the inner peristome has a shallow incision. These are the distinguishing characters of *Opisthoporus*; therefore, we have relocated this species into the genus *Opisthoporus*.

#### ﻿ Opisthoporus grohi

Taxon classificationAnimaliaArchitaenioglossaCyclophoridae

(Thach, 2020)

817FB1AD-02F4-53D9-88DA-7D77D5409829

Fig. 57F

Rhiostoma grohi Thach, 2020: 18, 19, figs 155, 156. Type locality: Krong No District, Dak Nong Province, Central Vietnam.

##### Type material.

***Holotype***MNHN-IM-2000-35507 (Fig. 57F) from Krông Nô District, Dàk Nông Province.

##### Remarks.

This species description was based on the holotype and two paratypes housed in the Thatch’s collection. The holotype is probably a young or not fully-grown specimen because of the slightly thin and sharp apertural lip. A short detached whorl and short breathing tube located away from the apertural lip are observed in the holotype. The operculum accompanying the type series (see Thach 2020: fig. 156) and the statement in the original description “exteriorly whitish with many concentric layers” seem to indicate a plate-shaped operculum. These are the diagnostic characters, and therefore, we have relocated this species into the genus *Opisthoporus*.

#### ﻿ Opisthoporus herosae

Taxon classificationAnimaliaArchitaenioglossaCyclophoridae

(Thach & Huber, 2017)

B7C2460A-F61F-5892-AEB8-805F08AB074F

Fig. 58A, B

Rhiostoma herosae Thach & Huber in Thach 2017: 17, figs 87–89. Type locality: Ninh Binh Province, North Vietnam. Páll-Gergely et al. 2020a: 40. Do et al. 2020b: 166, fig. 1a.
Rhiostoma ninhbinhensis Thach & Huber in Thach 2018: 17, figs 81a, 82a, 83a, b. Type locality: Ninh Binh Province, Central Vietnam.

##### Type material.

**Figure 58.**
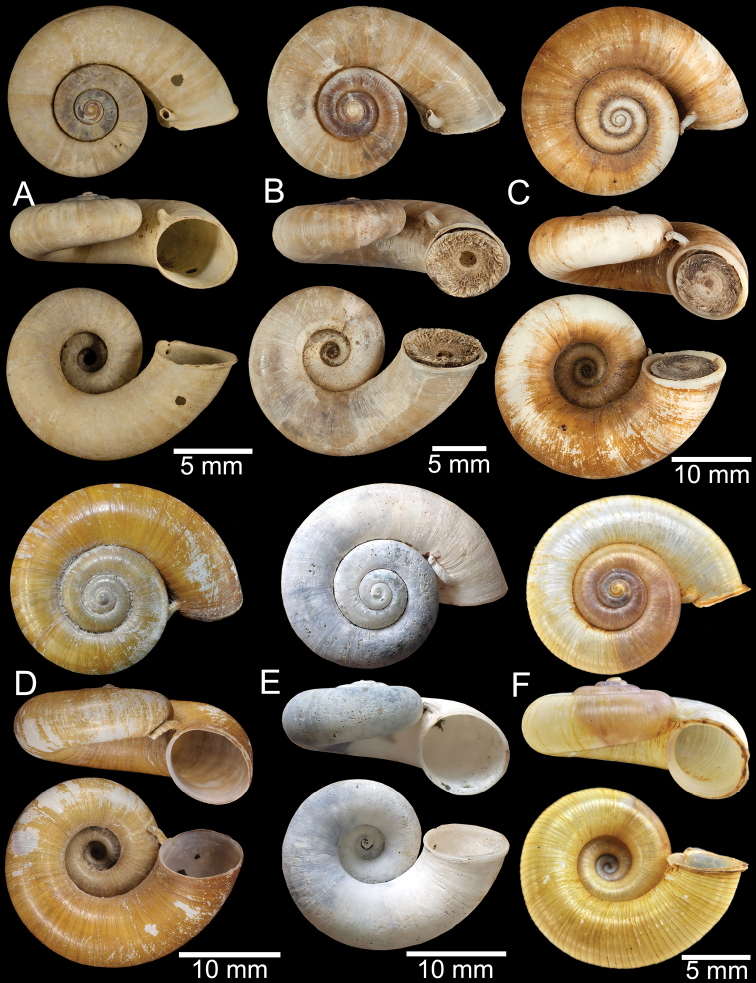
**A, B***Opisthoporusherosae***A** holotype MNHN-IM-2000-33198 from Ninh Binh, Vietnam and **B** holotype FMNH 386285 of *Rhiostomaninhbinhensis* Thach & Huber, 201, from Ninh Binh, Vietnam **C***Opisthoporusngocngai* holotype FMNH 386291 from Ninh Hao, Vietnam **D***Opisthoporusngocthachi* holotype MNHN-IM-2000-35508 from Khanh Hoa, Vietnam **E***Opisthoporusthorsengi* holotype BOR/MOL 14587 from Dak Nong, Vietnam **F***Opisthoporustener* specimen NHMUK ex. Cuming collection. Photographs: M Caballer, A Lardeur and V Heros, MNHN (**A, D, F**), J Gerber, FMNH (**B, C**), T-S Liew, BORNEENSIS (**E**).

***Holotype***MNHN-IM-2000-33198 (Fig. 58A) from Ninh Binh Province. ***Holotype***FMNH 386285 (Fig. 58B) of *Rhiostomaninhbinhensis* Thach & Huber, 2018, from Ninh Bình, Vietnam.

##### Remarks.

This species description was based on the holotype and four paratypes. Páll-Gergely et al. (2020a) stated that the holotypes of *R.herosae* and *R.ninhbinhensis* are identical in all shell size characters and they share the same type locality in Ninh Binh Province; therefore, they treated the latter as a junior synonym of the former species. Do et al. (2020b) agreed with this treatment and have made several attempts to collect *R.herosae* from Ninh Binh Province (type locality) and neighbouring provinces, but have not found any specimens identifiable to this species.

This species differs from other *Rhiostoma* species in having a multispiral, slightly concave, and plate-shaped operculum, which are the distinguishing characters of the *Opisthoporus* (see Do et al. 2020a). We agree with the previous synonymisation and here have also placed this species in the genus *Opisthoporus*.

#### ﻿ Opisthoporus ngocngai

Taxon classificationAnimaliaArchitaenioglossaCyclophoridae

(Thach & Huber, 2018)

921C53BA-74BE-5F51-B2BA-E4B334D8EBBE

Fig. 58C

Rhiostoma ngocngai Thach & Huber in Thach, 2018: 17, figs 81b, 82b, 83c. Type locality: Ninh Tan Commune, Ninh Hao District, Central Vietnam [Khanh Hao Province].

##### Type material.

***Holotype***FMNH 386291 (Fig. 58C) from Ninh Hòa, Khánh Hòa, Vietnam.

##### Remarks.

This species was described based on a single specimen. Although Do et al. (2020a) treated this as a junior synonym of *O.herosae*, it differs by having larger shells (width 30 mm), tubular-shaped breathing device located perpendicular to the last whorl, detached whorl short, and lip slightly thickened but not expanded. These minor differences have been used as diagnostic characters of *Opisthoporus* species in Vietnam (see Do et al. 2020a). In addition, this species differs from *Rhiostoma* in having a multispiral, slightly concave, plate-shaped operculum (Do et al. 2020a). Therefore, we have retained this species as valid, but transferred it to the genus *Opisthoporus*. Further systematic work and more specimens of the species will verify the species status and relationship with other taxa.

#### ﻿ Opisthoporus ngocthachi

Taxon classificationAnimaliaArchitaenioglossaCyclophoridae

(Huber, 2020)

73426121-DAAE-58AB-88B4-57958F54E367

Fig. 58D

Rhiostoma ngocthachi Huber in Thach, 2020: 19, figs 159, 160. Type locality: Ninh Hoa District, Khanh Hoa Province, Central Vietnam.

##### Type material.

***Holotype***MNHN-IM-2000-35508 (Fig. 58D) from Ninh Hoa District, Khanh Hoa Province.

##### Remarks.

This species was described based on a single specimen. The original description states “operculum exteriorly white with many concentric layers and interiorly glossy, dark brown with a central nucleus and extremely thick margin”. In addition, the shell bears a nearly flattened spire, a monochrome yellowish brown periostracum, the lip slightly thickened but not expanded, and tubular breathing device located away from the apertural lip, which are the diagnostic characters of the genus *Opisthoporus*. Therefore, we have reclassified this species to the genus *Opisthoporus*.

#### ﻿ Opisthoporus thorsengi

Taxon classificationAnimaliaArchitaenioglossaCyclophoridae

(Thach, 2020)

4DC223C4-E310-50C7-A640-5D5CECC18000

Fig. 58E

Rhiostoma thorsengi Thach, 2020: 19, 20, figs 157, 158. Type locality: Krong No District, Dak Nong Province, Central Vietnam.

##### Type material.

***Holotype***BOR/MOL 14587 (Fig. 58E) from Krong No District, Dak Nong Province.

##### Remarks.

This species was described based on a single specimen with a worn shell, eroded protoconch, and without a periostracum. The unique characters are a circular peristome, slightly thickened but not expanded lip, a tubular breathing device curved and pointed posteriorly, and a short detached whorl. These characteristics have led us to classify this species as a member of the *Opisthoporus* (see Do et al. 2020a).

#### ﻿ Opisthoporus tener

Taxon classificationAnimaliaArchitaenioglossaCyclophoridae

(Menke, 1856)

B0C10E8A-B3F4-5845-B2DA-30081E442D68

Fig. 58F

Pterocyclos tener Menke, 1856: 69. Type locality: Touranne, Cochinchinae [Da Nang Province, Vietnam]. Pfeiffer 1856: 90, 91, pl. 25, figs 13–15. Pfeiffer 1858: 32. Reeve 1863: Pterocyclos pl. 5, species 28.
Rhiostoma tener —Benson 1860: 97.
Rhiostoma tenerum [sic]—Pfeiffer 1865: 40.
Opisthoporus tener —Kobelt and Möllendorff 1897: 119.
Cyclotus (Siphonocyclus) tener —Möllendorff 1900: 135, 136. Kobelt 1902: 212

##### Other material examined.

Specimen NHMUK ex. Cuming collection (3 shells; Fig. 58F) from Cochin China.

##### Remarks.

The type specimens of this species could not be located. However, the NHM collection contains a lot of three shells from the Cuming collection with an original label, probably in Pfeiffer’s handwriting, stating the species name and collection locality, but this label was subsequently overwritten. One of these three shells matches well with the illustration in Pfeiffer (1856: pl. 25, figs 13–15). These specimens exhibit unique characteristics, including a short detached whorl and a breathing device with a short tubular shape pointed anteriorly. These characteristics suggest that it be classified as a member of the *Opisthoporus*.

## ﻿Discussion

This article presents the first revision on the diversification of the snorkel snail genus *Rhiostoma* based on morphological and molecular perspectives. This integrative approach used the complementary information from COI barcoding sequences and morphological traits to define the species. This work encompasses all species originally or subsequently attributed to the *Rhiostoma*, which comprise 30 species including the 12 new species that are recognised and described herein. Among these, only one species with an existing name was known from the type material with a vague type locality. The previous references to the species attributed to the *Rhiostoma* listed in the “Species excluded” must be regarded with caution. Among the historical species, the topotype specimen of *Rhiostomajousseaumei* Morgan, 1885 from peninsular Malaysia (Fig. 57B, C), has an operculum more similar to that of *Pterocyclos*, while *Rhiostomaboxalli* Godwin-Austen, 1893 from Palawan Islands, Philippines, has an operculum and expanded outer lip more similar to those of *Cyclotus*. Recently, Thach (2017, 2018, 2020) identified six cyclophorid snails using the name *Rhiostoma*. Although the specimens examined for these identifications are limited, examination of the type specimens is sufficient to conclude that all six species are not closely related to the *Rhiostoma* s. s. Their opercular structures more resembles those of *Opisthoporus*, and they are clearly misidentified, suggesting reallocation to another genus. Additional materials for these nine excluded species are necessary for further comprehensive systematic revision.

This snorkel snail genus *Rhiostoma* has a distribution centred in mainland Southeast Asia. It closely resembles the Pterocyclini genera *Pterocyclos* and *Spiraculum*, but there are several noticeable morphological differences between them. First, the breathing devices in the *Rhiostoma*, with shapes ranging from notched to tubular, are located on the upper junction of the peristome near the suture, and without wing shape at the outer lip. In contrast, *Pterocyclos* has a narrow to wide slit with an outer lip expansion (wing shaped) at the upper junction of the peristome, while *Spiraculum* shows both a wing-shaped outer lip and a tubular breathing device located in the suture, away from the apertural lip. Second, a heavily calcified and cup-shaped operculum is present in *Rhiostoma*, whereas the operculum is partially calcified with a dome to plate shape in the other two genera.

It is widely accepted that the operculum in gastropods plays a primary role as a passive defensive structure for avoiding various predators attacking through the shell aperture (Kelly and Cory 1987; Norton 1988; Vermeij 2015; Sato 2019). In addition, it further protects the terrestrial snail soft body from arid environments and desiccation (Páll-Gergely et al. 2016b). Moreover, operculum structure is another character commonly used for higher-level taxonomic purposes and has proved to carry a phylogenetic signal (Ponder 1998; Kaim and Sztajner 2005; Wilmsmeier and Neubert 2012; Golding et al. 2014; Simone 2020). For instance, in the terbinid marine vertigastropods, the primitive state is a corneous operculum, while a calcareous operculum occurs in more derived members (Checa and Jiménez-Jiménez 1998; Williams and Ozawa 2006; Vermeij and Williams 2007). In the Cyclophoridae, terrestrial caenogastropods, opercula have tended to evolve in various degrees of calcification, ranging from non-calcified (corneous, i.e., *Cyclophorus* and *Leptopoma*) to partially calcified (i.e., *Pterocyclos*) and heavily calcified (i.e., *Opisthoporus*, *Rhiostoma*). Although the opercular morphology of the *Rhiostoma* is generally conserved, it can provide relatively high resolving power among the Pterocyclini genera (Fig. 5; Table 2) and some minor opercular characters have been identified as diagnostic, i.e., *Rhiostomalannaense* sp. nov. Unfortunately, an examination of the overall cyclophorid phylogeny has never been undertaken, and it remains to be seen whether the basal cyclophorid lineages have thinner opercula (corneous) and calcareous opercula occur in the later-emerging lineages.

The terrestrial caenogastropod operculum has at least two main functions: sealing the shell aperture against predators and protecting the soft body from dry weather. In the *Rhiostoma*, the operculum is made of thick calcium carbonate, which is impermeable or difficult for any chemical substance to penetrate, either in liquid or gas phase. The snails, therefore, have evolved significant shell apparatus to assist in gas exchange while the operculum seals the shell aperture. Within the genus *Rhiostoma*, the breathing device is well developed, distinct, and placed on the upper junction of the peristome near the suture. It is diverse in shape, from knob or notch (formed by protrusion of the outer lip and with or without groove) to short or long tubular (a snorkel-like structure). Regardless of phylogeny, breathing device character stages are relatively consistent, showing less variation within all recognised species, except in the case of *Rhiostomahainesi*. The different notch and tubular shapes have been used to distinguish *Rhiostomasmithi* from *Rhiostomatomlini*. Although the COI barcoding does not reflect the actual evolutionary relationship, it seemed to provide valuable traits for species-level classification; thus, those two species are synonymised with *Rhiostomahainesi*. The complex gas-exchange structures have been detailed in studies of some alycaeid and vermitid caenogastropods, and the authors suggest that they can provide useful characteristics for higher-level systematics (Páll-Gergely et al. 2016b, 2017, 2020b; Bieler et al. 2019; Jirapatrasilp et al. 2022).

Another morphological character that has been used extensively to distinguish *Rhiostoma* species is the terminal part of the last whorl, which can be detached from the preceding whorl. For example, *R.samuiense* from peninsular Malaysia (“*chupingense*” morphotype) and specimens from Khao Luang Mountains (“proboscis” morphotype) have distinctly longer detached whorls than the typical *R.samuiense* (Fig. 14). The COI barcoding of those topotypes and Khao Luang specimens are clustered together and show very low genetic divergence. A similar situation also occurs between *R.jalorensis* and “*R.huberi* Thach, 2018”. Recognition of species-level diversity in the *Rhiostoma* by this character alone is inadequate due to extreme variability. A combined dataset of multiple morphological characters and molecular phylogeny will enhance the species recognition for this genus.

This revisionary work provides a significant contribution to the taxonomic knowledge of the pterocyclinid snails in Indochina in particular, especially by the incorporation of COI barcoding for species delineation. In addition, fairly high intra-specific genetic divergences were observed between populations of *R.jalorensis* (average 6.69% p-distance) and *R.housei* (3.67%), suggesting lower dispersal rates and more isolated populations, although the populations are in close proximity.

The clade composed of *R.hainesi*, *R.samuiense*, *R.asiphon*, and *R.rhothonotaphrosa* sp. nov. are externally diverse with clear external features that can be used for species identification. However, these four taxa show relatively lower inter-specific genetic divergence (3.30–3.81%) than the average among *Rhiostoma* species (15.33%). This may be due to recent diversification and local adaptations to limestone and non-limestone habitats, factors which are obviously reflected by the diverse morphology. Geographic and habitat isolation may have played an essential role in the diversification of the *Rhiostoma*; phylogenetic studies with more markers are needed to understand the evolutionary history of these snorkel snails. Further study of systematics and phylogeny that encompasses the full range of morphological diversity in the pterocyclinid genera will elucidate the evolutionary trends and reveal the true diversity of this tribe.

## Supplementary Material

XML Treatment for Rhiostoma

XML Treatment for Rhiostoma haughtoni

XML Treatment for Rhiostoma samuiense

XML Treatment for Rhiostoma rhothonotaphrosa

XML Treatment for Rhiostoma housei

XML Treatment for Rhiostoma hainesi

XML Treatment for Rhiostoma simplicilabre

XML Treatment for Rhiostoma marioni

XML Treatment for Rhiostoma dalyi

XML Treatment for Rhiostoma jalorensis

XML Treatment for Rhiostoma thachi

XML Treatment for Rhiostoma ebenozostera

XML Treatment for Rhiostoma lannaense

XML Treatment for Rhiostoma tigrina

XML Treatment for Rhiostoma asiphon

XML Treatment for Rhiostoma strubelli

XML Treatment for Rhiostoma abletti

XML Treatment for Rhiostoma anceyi

XML Treatment for Rhiostoma breviocollar

XML Treatment for Rhiostoma furfurosum

XML Treatment for Rhiostoma platymorpha

XML Treatment for Rhiostoma cochinchinensis

XML Treatment for Rhiostoma cambodjense

XML Treatment for Rhiostoma morleti

XML Treatment for Rhiostoma prestoni

XML Treatment for Rhiostoma cheliopegma

XML Treatment for Rhiostoma gnomus

XML Treatment for Rhiostoma laosense

XML Treatment for Rhiostoma ? americana

XML Treatment for Rhiostoma ? amarapuraense

XML Treatment for Rhiostoma

XML Treatment for Pterocyclos

XML Treatment for Pterocyclos jousseaumei

XML Treatment for Cyclotus

XML Treatment for Cyclotus boxalli

XML Treatment for Opisthoporus

XML Treatment for Opisthoporus battambangensis

XML Treatment for Opisthoporus grohi

XML Treatment for Opisthoporus herosae

XML Treatment for Opisthoporus ngocngai

XML Treatment for Opisthoporus ngocthachi

XML Treatment for Opisthoporus thorsengi

XML Treatment for Opisthoporus tener

## ﻿Appendix 1

**Table A1.** Average interspecific genetic divergence (uncorrected p-distance: % ± S.E.) between *Rhiostoma* species (below diagonal) calculated from 660-bp *COI* gene fragment sequences. Average intraspecific distances within each species are shown in bold.

Taxa	1.	2.	3.	4.	5.	6.	7.	8.	9.	10.	11.	12.	13.	14.	15.	16.	17.	18.
1. *R.cheliopegma* sp. nov.	**3.17** ± **0.46**																	
2. *R.housei*	11.51 ± 1.01	**3.67** ± **0.47**																
3. *R.breviocollar* sp. nov.	12.61 ± 1.15	8.58 ± 0.92	**0.15** ± **0.15**															
4. *R.furfurosum* sp. nov.	13.67 ± 1.15	13.26 ± 1.16	14.03 ± 1.29	**1.25** ± **0.27**														
5. *R.samuiense*	16.21 ± 1.27	17.82 ± 1.28	18.56 ± 1.38	16.85 ± 1.30	**1.78** ± **0.32**													
6. *R.asiphon*	17.28 ± 1.31	18.02 ± 1.36	17.54 ± 1.36	16.95 ± 1.34	3.81 ± 0.63	**0.72** ± **0.25**												
7. *R.rhothonotaphrosa* sp. nov.	17.20 ± 1.30	18.03 ± 1.31	17.85 ± 1.35	17.56 ± 1.37	3.39 ± 0.57	3.30 ± 0.61	**0.93** ± **0.30**											
8. *R.hainesi*	15.95 ± 1.30	17.84 ± 1.35	17.39 ± 1.36	16.46 ± 1.34	3.58 ± 0.62	3.50 ± 0.67	3.32 ± 0.65	**0.62** ± **0.30**										
9. *R.jalorensis*	15.02 ± 1.12	14.81 ± 1.14	15.27 ± 1.22	15.19 ± 1.12	13.67 ± 1.04	13.67 ± 1.06	14.22 ± 1.07	13.45 ± 1.04	**6.69** ± **0.55**									
10. *R.platymorpha* sp. nov.	17.27 ± 1.30	17.94 ± 1.31	16.06 ± 1.34	16.18 ± 1.32	17.17 ± 1.40	17.48 ± 1.43	17.17 ± 1.38	16.75 ± 1.41	14.74 ± 1.17	**0.19** ± **0.10**								
11. *R.tigrina* sp. nov.	16.90 ± 1.29	17.64 ± 1.29	16.87 ± 1.35	16.83 ± 1.29	17.24 ± 1.34	17.41 ± 1.34	17.00 ± 1.31	16.77 ± 1.35	16.26 ± 1.21	6.02 ± 0.87	**1.03** ± **0.31**							
12. *R.haughtoni*	15.92 ± 1.30	15.81 ± 1.29	16.46 ± 1.35	17.35 ± 1.33	19.32 ± 1.31	19.06 ± 1.33	19.01 ± 1.35	18.01 ± 1.35	17.07 ± 1.24	13.97 ± 1.30	13.96 ± 1.26	**n/c**						
13. *R.dalyi*	14.64 ± 1.22	15.43 ± 1.25	16.00 ± 1.35	15.88 ± 1.28	18.13 ± 1.30	18.70 ± 1.38	18.70 ± 1.32	18.01 ± 1.36	15.79 ± 1.17	14.93 ± 1.25	16.13 ± 1.27	15.30 ± 1.31	**0.00** ± **0.00**					
14. *R.lannaense* sp. nov.	16.14 ± 1.26	16.45 ± 1.28	15.79 ± 1.35	17.26 ± 1.30	16.70 ± 1.26	16.88 ± 1.28	16.54 ± 1.28	16.20 ± 1.27	16.14 ± 1.14	16.17 ± 1.31	15.99 ± 1.32	14.35 ± 1.30	14.63 ± 1.23	**1.59** ± **0.30**				
15. *R.abletti*	14.36 ± 1.15	16.59 ± 1.31	17.18 ± 1.38	15.89 ± 1.25	16.82 ± 1.28	17.29 ± 1.32	17.33 ± 1.31	15.46 ± 1.26	15.77 ± 1.15	15.55 ± 1.38	15.52 ± 1.30	15.97 ± 1.38	14.73 ± 1.25	15.42 ± 1.25	**1.85** ± **0.42**			
16. *R.simplicilabre*	14.59 ± 1.20	16.69 ± 1.31	16.15 ± 1.36	17.43 ± 1.36	16.78 ± 1.32	17.36 ± 1.35	17.16 ± 1.32	15.77 ± 1.33	16.07 ± 1.17	16.48 ± 1.37	17.00 ± 1.31	16.07 ± 1.38	14.99 ± 1.29	16.31 ± 1.30	4.84 ± 0.73	**0.00** ± **0.00**		
17. *R.morleti*	14.84 ± 1.16	16.50 ± 1.23	16.38 ± 1.34	17.00 ± 1.31	15.97 ± 1.29	17.18 ± 1.34	16.77 ± 1.32	15.84 ± 1.31	17.10 ± 1.23	16.86 ± 1.38	16.82 ± 1.32	16.77 ± 1.36	13.68 ± 1.18	14.91 ± 1.26	6.77 ± 0.90	7.03 ± 0.92	**0.46** ± **0.26**	
18. *R.cambodjense*	13.63 ± 1.20	15.19 ± 1.27	14.49 ± 1.29	14.99 ± 1.28	16.16 ± 1.31	15.96 ± 1.33	15.80 ± 1.27	15.57 ± 1.30	14.37 ± 1.15	15.09 ± 1.35	15.65 ± 1.34	14.72 ± 1.27	14.26 ± 1.24	15.42 ± 1.26	15.60 ± 1.28	16.58 ± 1.30	15.42 ± 1.28	**0.15** ± **0.10**
